# The Natufian Epipalaeolithic and Pre-Pottery Neolithic in the desert of northern Arabia

**DOI:** 10.1038/s41598-026-40541-3

**Published:** 2026-03-02

**Authors:** Ceri Shipton, Maria Guagnin, Faisal Al-Jibreen, Finn Stileman, Mathew Stewart, Simon J. Armitage, Nick Drake, Christian Reepmeyer, Paul S. Breeze, Frans van Buchem, Fahad Al-Tamimi, Muhammed Al-Shamry, Ahmed Al-Shammari, Jaber Al-Wadani, James Blinkhorn, Abdullah M. Alsharekh, Michael Petraglia

**Affiliations:** 1 College of Asia and the Pacific, Australian National University, Canberra, Australia; 2 Institute of Archaeology, University College London, London, UK; 3 Department of Archaeology, Max Planck Institute of Geoanthropology, Jena, Germany; 4 School of Humanities, The University of Sydney, Sydney, Australia; 5 Heritage Commission, Saudi Ministry of Culture, Riyadh, Saudi Arabia; 6 Department of Archaeology, University of Cambridge, Cambridge, UK; 7 Australian Research Centre for Human Evolution, Griffith University, Brisbane, Australia; 8 Department of Geography, Royal Holloway University of London, Egham, UK; 9 SFF Centre for Early Sapiens Behaviour (SapienCE), University of Bergen, Post Box 7805, 5020 Bergen, Norway; 10 Department of Geography, King’s College London, London, UK; 11 Commission for Archaeology of Non-European Cultures, German Archaeological Institute, Dürenstrasse 35-37, 53173 Bonn-Bad Godesberg, Germany; 12 Physical Science and Engineering Division, King Abdullah University of Science and Technology (KAUST), Thuwal, Saudi Arabia; 13 Department of Archaeology, Classics and Egyptology, University of Liverpool, Liverpool, UK; 14 Human Palaeosystems Group, Max Planck Institute of Geoanthropology, Jena, Germany; 15 Department of Archaeology, College of Tourism and Archaeology, King Saud University, Riyadh, Saudi Arabia; 16 Human Origins Program, Smithsonian Institution, Washington, D.C. USA; 17 School of Social Science, University of Queensland, Brisbane, Australia

**Keywords:** PPNA, PPNB, Sahout, Helwan bladelets, Helwan points, Obsidian sourcing, Camel rock art, Ecology, Ecology, Evolution

## Abstract

The Fertile Crescent region, spanning from the upper Euphrates to the head of the Gulf of Aqaba, witnessed the earliest transition in the world from hunting and gathering wild foods to farming domesticates. Natufian Epipalaeolithic and Pre-Pottery Neolithic (PPN) communities in this region created distinctive stone tools across the Pleistocene-Holocene transition. Here we present archaeological evidence from the site of Sahout, demonstrating that communities using material culture characteristic of the Natufian and PPN were also present hundreds of kilometres south of the Fertile Crescent, in the much more arid interior of the Arabian Peninsula. Repeated occurrence of distinctive stone tools from 13.5 to 8.7 thousand years ago indicates intimate links to the Levant; showing both the far greater scale of these cultural connections than previously known, and the capacity of Natufian and PPN tool makers to subsist in marginal environments. Obsidian sourcing shows long-distance movement further southward into Arabia. At Sahout, PPN tools are associated with a regional rock art tradition of naturalistic life-sized camel engravings, which overlie representations of curvaceous women. The material culture of these communities suggests that long-term survival in relatively arid environments was based on a combination of local adaptation and a network of long-distance connections.

**Supplementary Information:**

The online version contains supplementary material available at 10.1038/s41598-026-40541-3.

## Introduction

**Fig. 1:**
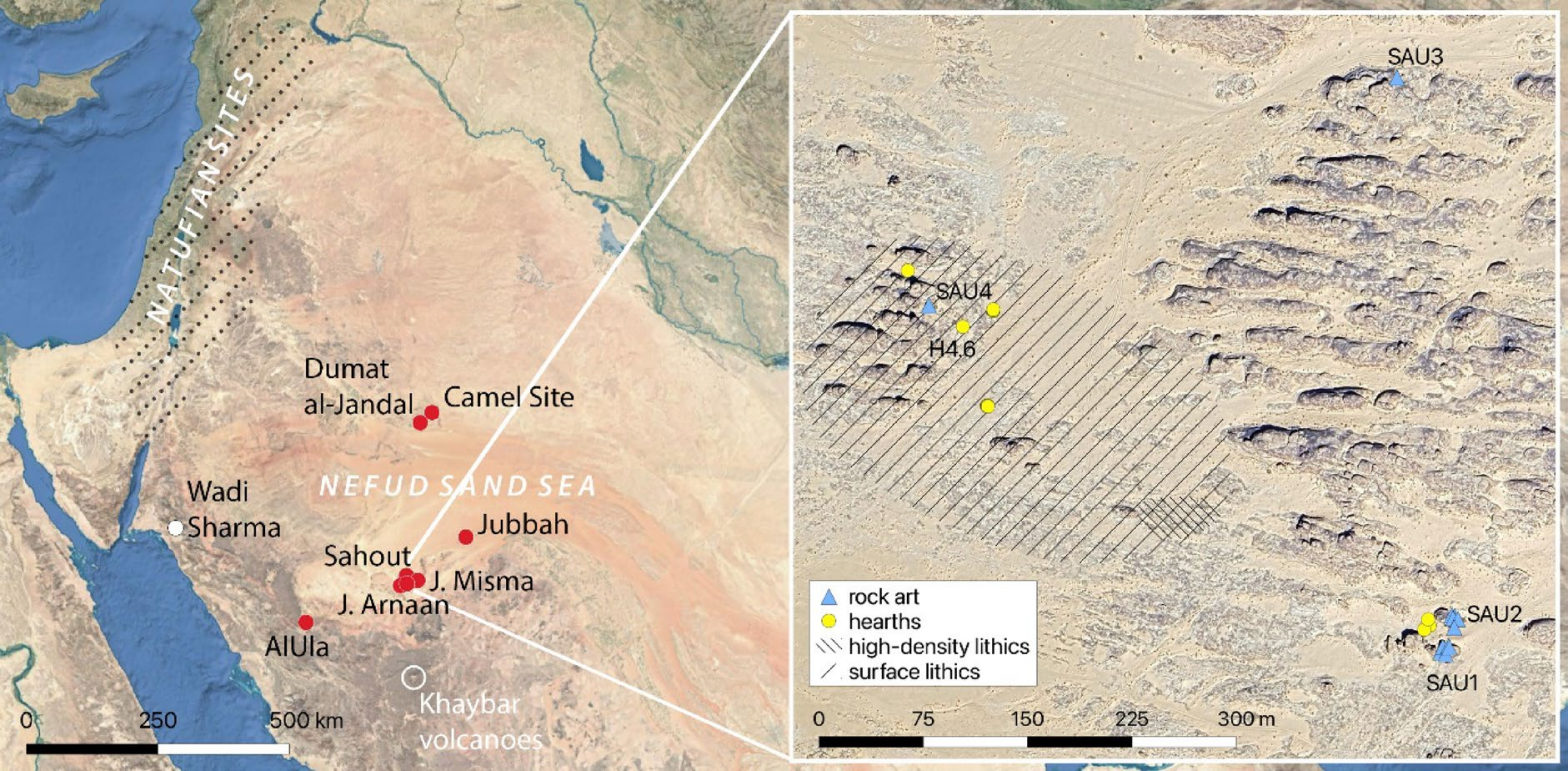
Map of Sahout and sites mentioned in the text. Red: Sites with large naturalistic camel engravings extending north, south, and west of the Nefud desert: Dumat al-Jandal, Jubbah, Sahout, Jebel Misma, and Jebel Arnaan also have PPN stone artefacts. Note the Jubbah Oasis includes both the sites of Al-Rabyah and Jebel Qattar. White: site with PPN artefacts but no rock art. Inset shows the localities within the Sahout site. The area with high-density lithics is interpreted as a silcrete workshop. Map generated using QGIS version 3.34.15-Prizren. Main map: Natural Earth base map. Inset: Google, Maxar Technologies, produced using Google Maps satellite imagery (Basemap layer in QGIS, https://qgis.org/,retrieved 06 October 2025).

In the Fertile Crescent, the Terminal Pleistocene to the early Holocene heralded the earliest transition in the world from hunting and gathering wild species to farming domesticated plants and animals. Three successive material culture groups have been associated with this transition: the Epipalaeolithic Natufian (~ 14.6–11.5 thousand years ago (ka)), the Pre-Pottery Neolithic A (PPNA ~ 11.7–10.5 ka), and the Pre-Pottery Neolithic B (PPNB, ~ 10.5–8.25 ka)^1–6^. These cultural entities have been mainly identified in the Levant and the upper reaches of the Euphrates and Tigris valleys, with Natufian sites apparently absent south of Jordan^7–9^ (Fig. 1). The moist environmental conditions and ‘mountain soils’ of the Fertile Crescent during the Pleistocene-Holocene transition are considered key for the emergence of farming^10^. Neighbouring arid regions including the Arabian Peninsula, were thought to have been inhospitable prior to the Holocene humid period ~ 10 − 6 ka, with widespread human occupation only emerging ~ 7.8–6.2 ka^11–13^.

Recent fieldwork in northern Arabia now suggests human presence in the early Holocene. On the northern side of the Nefud Desert, naviform blade production characteristic of the PPNB, was identified at several surface localities near Dumat al-Jandal^14^ (Fig. 1). In the Jubbah Oasis, in the central Nefud (Fig. 1), an Epipalaeolithic assemblage with affinities to the Geometric Kebaran of the Levant was documented at Al-Rabyah. Artefacts including backed and obliquely truncated bladelets as well as trapezoidal geometric microliths^15^, occurred on a deposit dated to 13.3 ka and buried by a deposit dating to 13.3 ka^16^. At the eastern end of the Jubbah Oasis, arrowheads of both PPNA and PPNB types, El-Khiam and Abu Salem (Helwan) points respectively, were found on the surface at Jebel Qatar, adjacent to a palaeolake dated to 8.7-8 ka^17^. To the southwest of the Jubbah Oasis, Abu Salem points were identified on the surface at Jebel Oraf, overlooking a palaeolake dating to 8.5 ka^18^. West of the Nefud, the southernmost early Holocene site known today is Wadi Sharma 1, near the northern end of the Red Sea where PPNB habitation in stone dwellings occurred at ~ 9.5-9 ka^19^.

South of the Nefud Desert, at Jebel Arnaan (Fig. 1), excavations recovered three El-Khiam points and occupations dating to 12.2 and 11.3 ka, as well as a Helwan bladelet and naviform core in non-stratified contexts^20^. East of Jebel Arnaan, an Abu Salem point was recovered on the surface at Jebel Misma (Fig. 1), with a naviform core in an undated stratified context^20^. However, the comparatively small quantities of diagnostic pieces, have limited our understanding of the populations that produced these artefacts.

These early occupations are reflected in the rock art record. In the Jubbah Oasis a widespread petroglyph tradition, known as the Jubbah Style with its elongate male human figures and bovids with exaggerated horns, corresponds with the later Neolithic of the Holocene humid period^21–23^. However, an earlier phase of petroglyphs, featuring women with curvaceous features, was identified at a cluster of boulders near the site of Al-Rabyah (Fig. 1), leading to the suggestion that these images relate to Epipalaeolithic occupation^23^. Images of curvaceous women were also documented at Jebel Arnaan where they were superimposed with engravings of life-sized camels in two cases. Excavations directly under a petroglyph of life-sized camels at Jebel Arnaan, revealed a stone tool used for pecking in a layer dated to 12.2 ka^20^. Dozens of life-sized camel engravings were documented on a cliff face at Jebel Misma, below which a pecking tool was found on the surface; excavations at this locality showed human occupation from ~ 12 ka. The age of earlier engravings of curvaceous women, and thus the origins of this petroglyph tradition, remain unknown.

Between Jebel Arnaan and Jebel Misma, test excavations at the site of Sahout recovered a single Helwan bladelet, an artefact diagnostic of the Natufian, and a radiocarbon date of 13.4 ka^24^. These tantalizingly suggest the Terminal Pleistocene occupation of northern Arabia by people with connections to the Fertile Crescent. Further non-diagnostic lithics and a date of 8.7 ka suggested a more extensive occupation sequence^24^. Here we report stratigraphically controlled excavations at Sahout to characterise these northern Arabian communities and assess their links to the Fertile Crescent.

Two trenches were excavated below a boulder (Supplementary Note 1), where the previous test trenches had yielded occupation deposits (Fig. 1). The archaeology of the wider site was explored through systematic surface collection at a lithic workshop and excavation of a hearth feature (Supplementary Note 1). Ages of excavated deposits were determined through luminescence (Supplementary Note 2) and radiocarbon dating (Supplementary Note 3). Faunal remains (Supplementary Note 4), grinding stones (Supplementary Note 5), and knapped lithics (Supplementary Note 6) are described. Geochemical fingerprinting is used to determine the source of occasional obsidian artefacts (Supplementary Note 7). Two rock art panels in the area were systematically documented to add to the corpus of rock art previously documented at the main excavation locality^24^ (Fig. 1) (Supplementary Note 8). We correlate the sequence of human occupation in the archaeological deposits with the sequence of rock art, both at the site of Sahout itself and in the broader region.

## Results

### The Sahout sequence

**Fig. 2:**
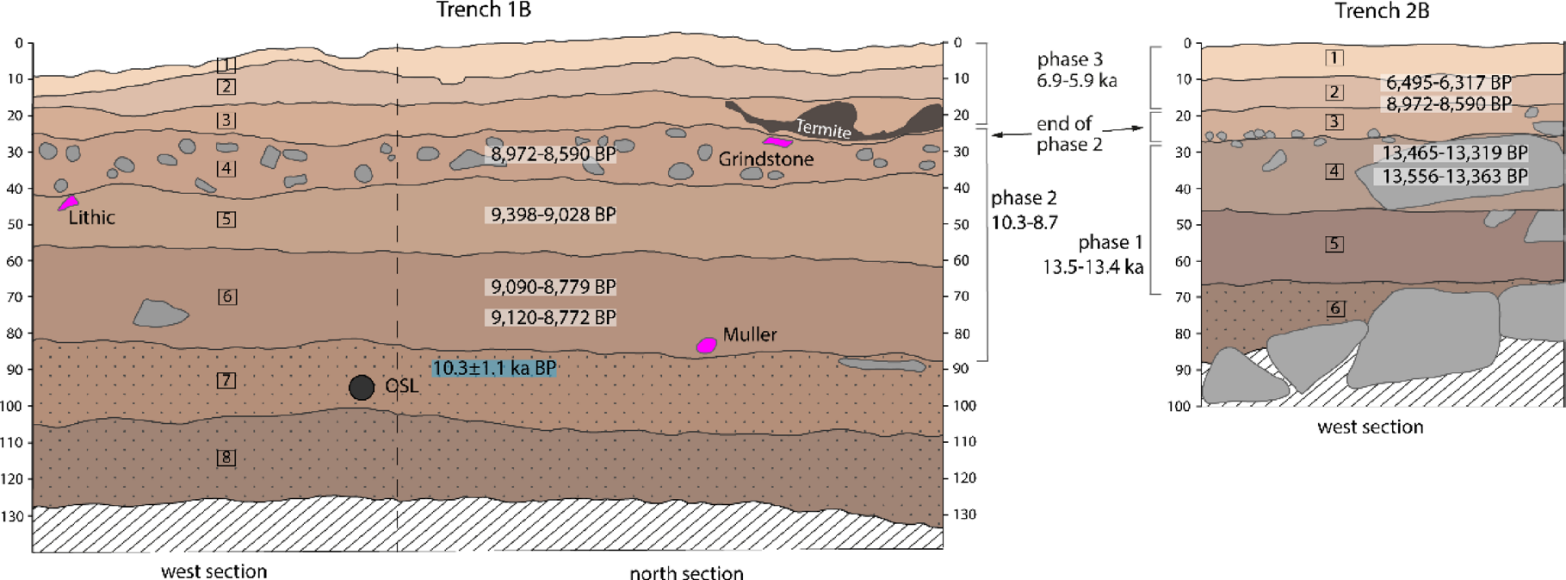
Section drawing of T1B (west and north) and T2B (west) showing the layers and corresponding phases. Radiocarbon (white background) and OSL ages (blue background) are listed according to layer. Key artefacts are shown in pink. Termite gallery shown in dark brown. Hatching indicates unexcavated natural deposit and dotted layers had no occupation (with exception of the top of layer 7). Vertical scale in cm.

The two new trenches were labelled T1B and T2B to distinguish them from the test trenches (T1A and T2A) they encompass. The trenches were located 1.5 m apart in the SAU2 rock shelter, with T1B measuring 1 × 1.5 m and T2B measuring 1 × 1 m (Supplementary Fig. 8). Eight horizontally bedded layers were differentiated in T1B (Fig. 2) (Supplementary Note 1). An optically stimulated luminescence (OSL) sample from layer 7 yielded an age of 10.3 ± 1.1 ka (Supplementary Note 2) and probably dates the onset of human occupation in T1B at the top of layer 7. Two radiocarbon ages showed layers 6 − 5 date to 9.2–8.9 ka (Fig. 2; Supplementary Fig. 10). Test excavation of T1A, carried out the previous year^24^, produced a date from the top of the clast-rich layer 4 of 8.7 ka (see Fig. 7 in^24^).

Five occupation layers were differentiated in SAU2 Trench 2B (T2B) underlain by the sterile poorly sorted sand layer 6, which sat on southward sloping sandstone slabs (Fig. 2). A radiocarbon date of 13.5 ka was obtained for layer 4. This was also the maximum depth for the test excavation T2A, from which a bone was similarly dated to 13.4 ka^24^ (Fig. 2). Layer 2 produced a bone dated to 8.9 ka and a bovid tooth dated to 6.4 ka, suggesting one of these was intrusive or that deflation has created a palimpsest layer. Previous excavation of two nearby hearths also showed there was occupation at the locality 6.9 and 5.9 ka^24^ (Supplementary Fig. 10).

Combining the absolute age estimates and stratigraphic information for T1B and T2B we distinguish three prehistoric occupation phases at Sahout: An early occupation phase (phase 1) in the lower part of T2B, layers 5 and 4, with dates of 13.5–13.4 ka; a middle phase (phase 2) beginning in the lower part of T1B (layers 6 − 5) and ending with a rubble layer in both trenches (layer 4 in T1B and Layer 3 in T2B) with dates spanning 10.3–8.7 ka; and a later phase (phase 3) in the upper layer of both trenches with ages of 6.9–5.9 ka. Phase 2 has the densest occupation across all main finds categories (Table 1).

**Table 1:** Major finds category counts by phase in the sahout (SAU2) excavations.

Find category	Phase 1	Phase 2	Phase 3	Total
Bone and teeth	64 (32%)	85 (42%)	54 (26%)	203 (100%)
Lithics	219 (12%)	1229 (70%)	327 (18%)	1775 (100%)
Grinding stones	2 (7%)	21 (78%)	4 (15%)	27 (100%)

Considering the 13.5 ka age for SAU2 phase 1 in T2B and the onset of occupation at 10.3 ka in T1B, we surmise that the main part of the site was scoured out by aeolian deflation (Sahout means place of the winds) during an arid phase in Arabia that peaked ~ 12 ka^25–27^. However, the sediment in T2B was protected from this deflation by the rock formation in front of the main boulder at SAU2 (Supplementary Fig. 2). The shelter provided by this nook might also have been more attractive during more arid periods of occupation when wind-blown sand would have been a hazard. This enclosed nook was perhaps not used during the early phase 2 occupation which coincides with the onset of the Holocene humid period resulting in a lack of sedimentation from that period. By contrast, a peak in lithic density in T1B layer 4, as well as most of the grinding stones from the excavation (Supplementary Note 5), suggests more intensive use of the locality extending across both trenches in the later part of phase 2.

### Fauna

An assemblage of 203 fragmentary animal bone specimens was recovered from the SAU2 T1B and T2B excavations. Calcium carbonate precipitate had cemented sand on to the surface of the bones while some specimens also showed manganese staining (*n* = 10), suggesting that wetter conditions than at present prevailed at the time of, or since, the deposition of the bones. The bones showed charring (*n* = 33) and calcination (*n* = 4) from burning, as well as green fractures suggesting breakage for marrow (Supplementary Note 4). Of the identifiable bones and teeth, 76% were ungulates. Gazelle occurred in all phases, with two *Bos* and two *Ovis/Capra* in phase 3.

### Lithics

**Fig. 3:**
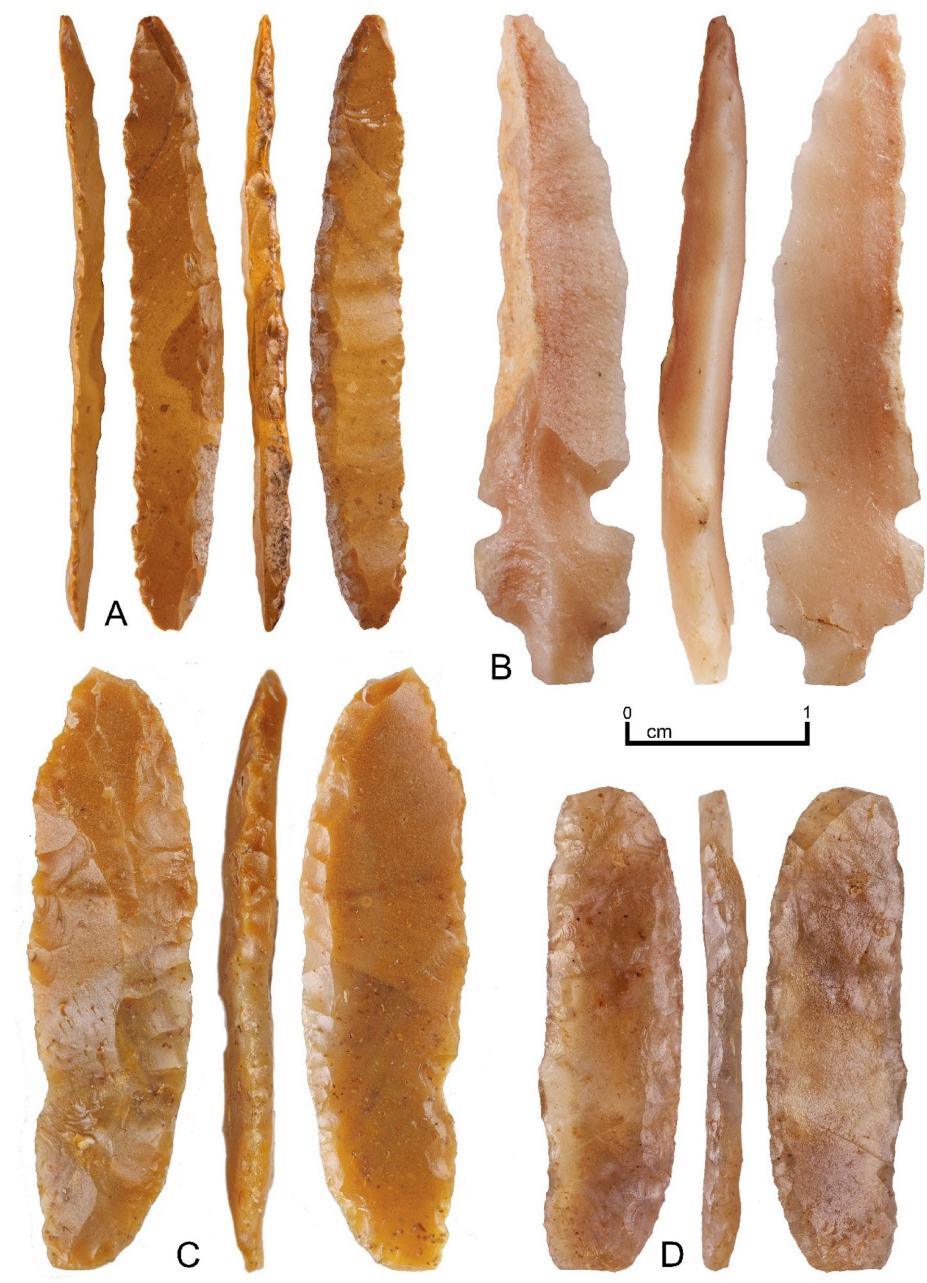
Helwan bladelets and Abu Salem point from the SAU2 excavations. (**A**) Chert Helwan bladelet from T1B layer 5; (**B**) Silcrete Abu Salem point from T1B layer 5; (**C**) Chert Helwan bladelet from T2B layer 4; (**D**) Chalcedony Helwan bladelet from T2B layer 2.

Phase 1 had the lowest density occupation in terms of lithic numbers (Table 1). Retouched artefacts occurred disproportionately in this phase, where they represented 6.9% of the assemblage, whereas in the rest of the sequence ~ 2% were retouched pieces (Supplementary Table 10). Helwan bladelets, with their distinctive invasive bifacial backing, were the main type of retouched artefact in T2B layer 4 (phase 1), with 5 specimens (Fig. 3, Supplementary Fig. 19). These artefacts are the principal diagnostic type of the Natufian in the Levant^28,29^. Such fine flake-removals on narrow blades could only be achieved through pressure flaking so these Helwan bladelets foreshadow the pressure flaked bifacial points of the PPN. T1B layers 4 and 5 (phase 2) featured PPN arrowhead types including a complete example of a silcrete Abu Salem point, a distinctive type made on a blade with both a tang and lateral notches at the base (Fig. 3). Systematic surface collection from the high density silcrete workshop locality produced two additional Abu Salem points, one unfinished, and the other apparently broken during the creation of one of the lateral notches (Supplementary Fig. 22). Silcrete naviform blade cores were found both in the excavated sequence in T1B layer 5 (phase 2), and on the surface at the workshop (Supplementary Figs. 14 & 15). Cresting blades found at the latter indicate the initiation of the blade production sequence (Supplementary Fig. 16). Nearby, the broken butt of a chert El Khiam point (Supplementary Fig. 25) was found on the surface, an artefact type also known from Jebel Arnaan^20^. Two end-scrapers with rounding wear were found in layers 4 and 5 in T2B (phase 1) (Supplementary Fig. 23), with two similar examples recovered from the Jebel Arnaan excavations. There were four blades with marginal retouch in the SAU2 sequence, with one from T1B layer 4 (phase 2) exhibiting sickle gloss (Supplementary Fig. 24). A later Neolithic Ha-Parsa arrowhead was found in T2B layer 2 (phase 3) (Supplementary Fig. 21).

Materials used for knapping vary markedly throughout the SAU2 sequence. In phase 1 red-yellow chert is the most common material (37%), with a dark blueish chert also in its highest proportions (8%), and silcrete constituting less than a quarter of artefacts (23%) (Supplementary Table 9). In phases 2 and 3, silcrete is the dominant material constituting at least half of the assemblage throughout T1B layers 6 − 1 and T2B layers 3 − 1 (Supplementary Note 6). There is also a reduction in diversity of materials, with Shannon’s Diversity Index showing that phase 2 is significantly less diverse than phase 1 (Supplementary Note 6). Fourteen obsidian artefacts were recovered from across both sequences.

### Obsidian sourcing

Geochemical characterisation was performed on 8 obsidian lithics from Sahout, 13 from Jebel Arnaan, 3 from Jebel Misma locality 8, and 9 experimental flakes of a geological sample from the putative source volcano in Khaybar, Jebel Al-Abyad (Supplementary Table 12). The Discriminant Functions analysis of Ti, Mn, Fe, Rb, Y, Zr, and Nb was successful in excluding all reference sources outside of the Khaybar region in the comparative dataset as a possible origin. The results plotted unambiguously with the Jebel Al-Abyad source ca. 190 km south of Sahout (Supplementary Fig. 27). In addition to the lithics observed at obsidian outcrops around the northern base of Jabal Al-Abyad^20^, we noted a large outcrop with obsidian debitage and a large hammerstone (Supplementary Fig. 28).

### Ground and decorative artefacts

The SAU2 T1B and T2B excavations produced 27 grinding stones, comprising 5 mullers and 22 bottom stones (Supplementary Note 5), with over three quarters of these from the phase 2 occupation (Table 1). A total of 287.1 g of red iron-rich shale ochre was recovered from T1B, and 173.6 g from T2B. Ochre was found in every occupation layer, including a crayon in T1B layer 5 (phase 2; Supplementary Fig. 13).

**Fig. 4:**
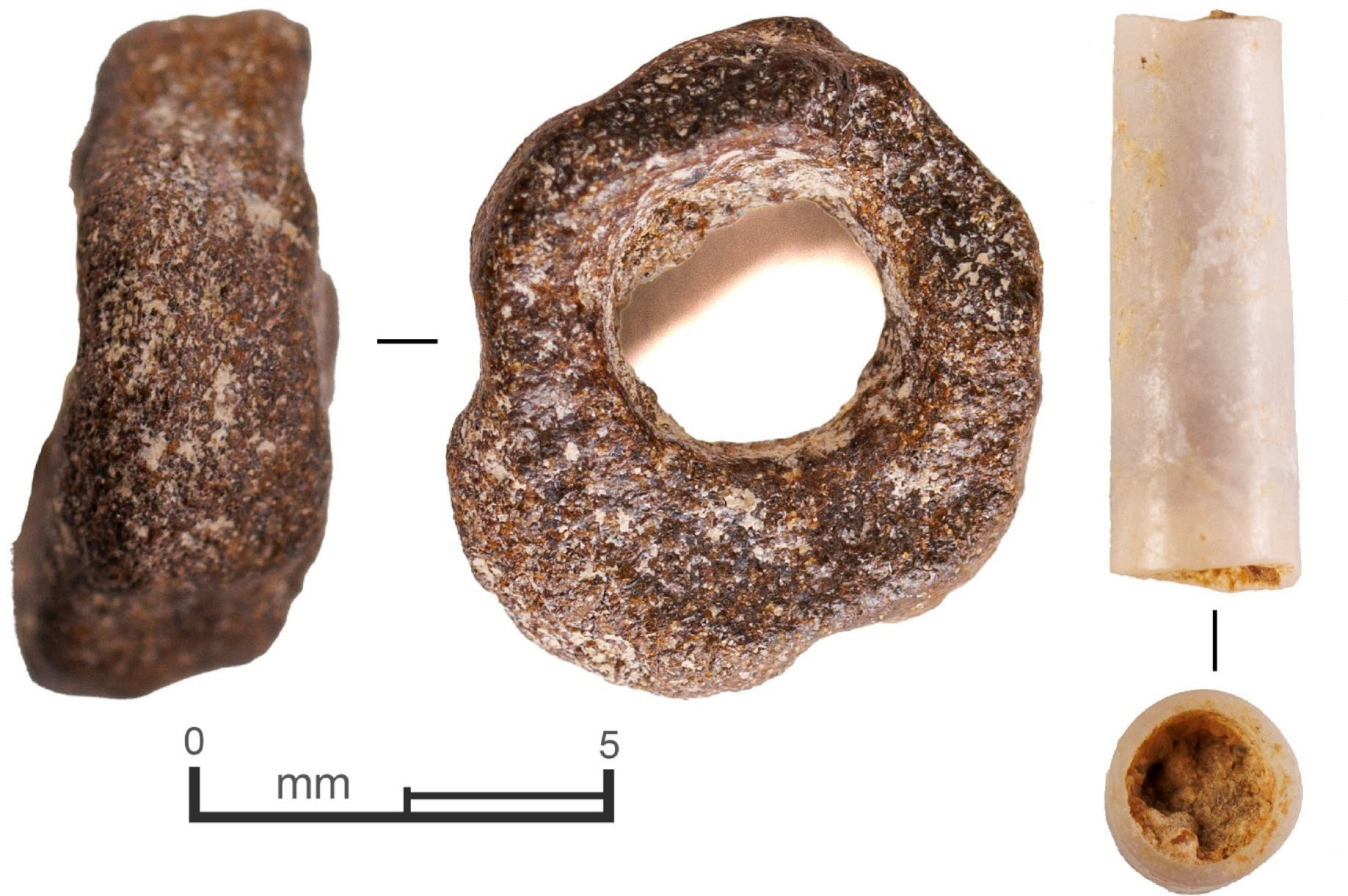
Small beads from phase 2, SAU2 T1B. Left: stone bead, layer 4; Right: *Dentalium* shell bead, layer 7.

Three beads were recovered from the SAU2 excavations: A large, broken stone bead from layer 4 in T2B (phase 1; Supplementary Fig. 13), a small stone bead from layer 4 in T1B (phase 2), and a small *Dentalium* bead from layer 7 in T1B (phase 2; Fig. 4). As it is a marine shell, the nearest source for this latter bead would be the Red Sea coast, at least 345 km away, with the Mediterranean coast the next nearest, being at least 700 km away.

### Rock art

**Fig. 5:**
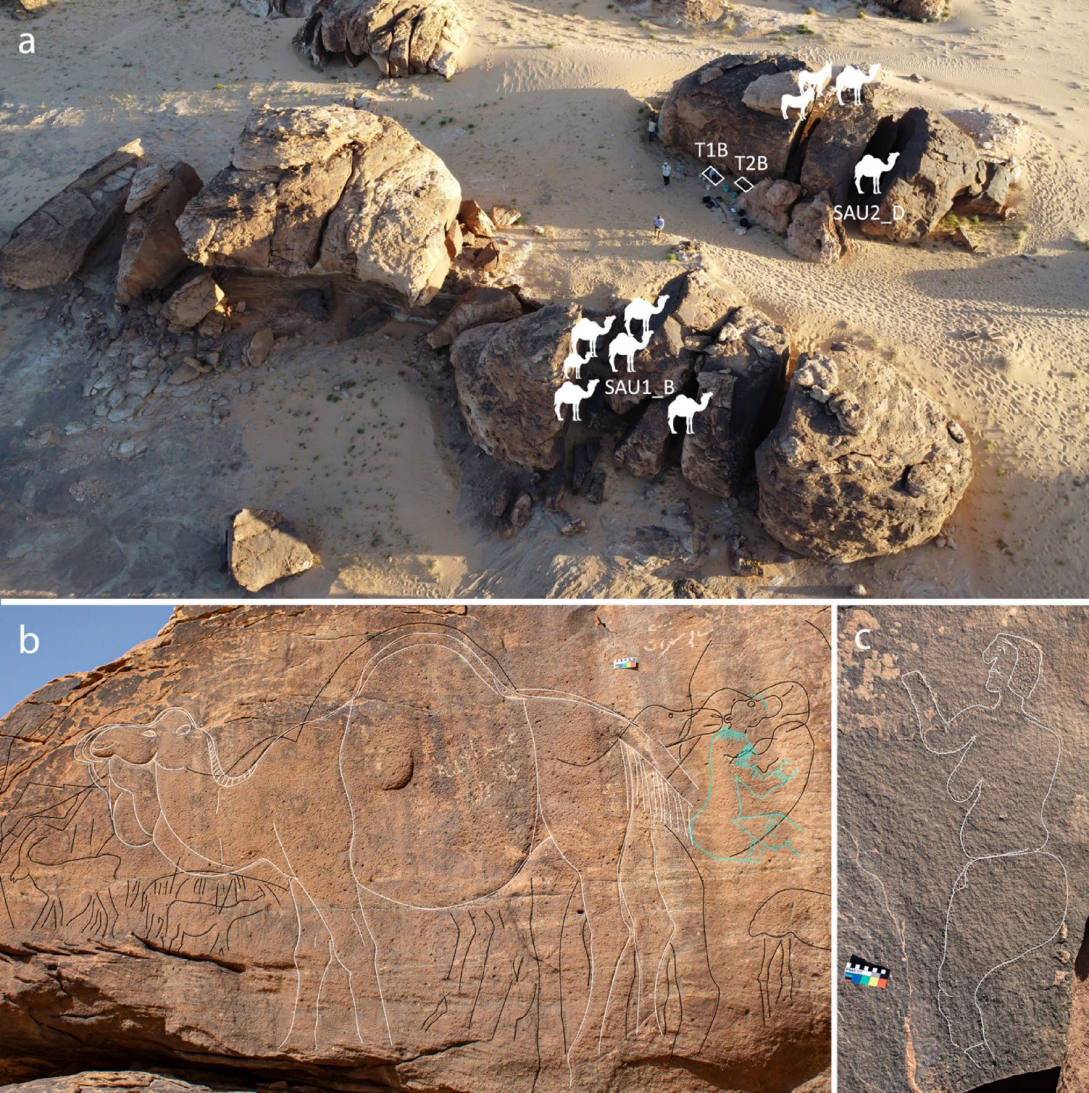
Sahout rock art. (**A**) Location of life-sized naturalistic petroglyphs of camels and equids at SAU1 and SAU2; Panels mentioned in the text are labelled. (**B**) Tracing of panel SAU3 showing a life-sized camel (traced in white), and numerous partial camel engravings and faded lines (traced in black), with several camel engravings superimposed over an engraving of a woman (traced in blue); (**C**) Tracing of panel SAU4. Scales in B and C are 10 cm. For original images see (Supplementary Figs. 30 and 31).

Life-sized naturalistic images dominate the rock art at the Sahout site^24^. The life-sized naturalistic petroglyphs from SAU1 and SAU2 feature eight images of camels and two wild equids (Fig. 5a). In addition, two previously unknown engravings of curvaceous women were documented. One image of a curvaceous woman in profile was found on a horizontal sandstone exposure at SAU4 (Figs. 1 and 5c; Supplementary Fig. 31). A further female figure was noted on panel SAU3 (Fig. 5b). This latter engraving is superimposed with multiple later camel engravings, and can today only be seen in optimal light conditions. It shows a sitting female figure with delicately engraved jewellery and decorations around the neck, chest and arm, possibly showing thin, elongated beads.

**Fig. 6:**
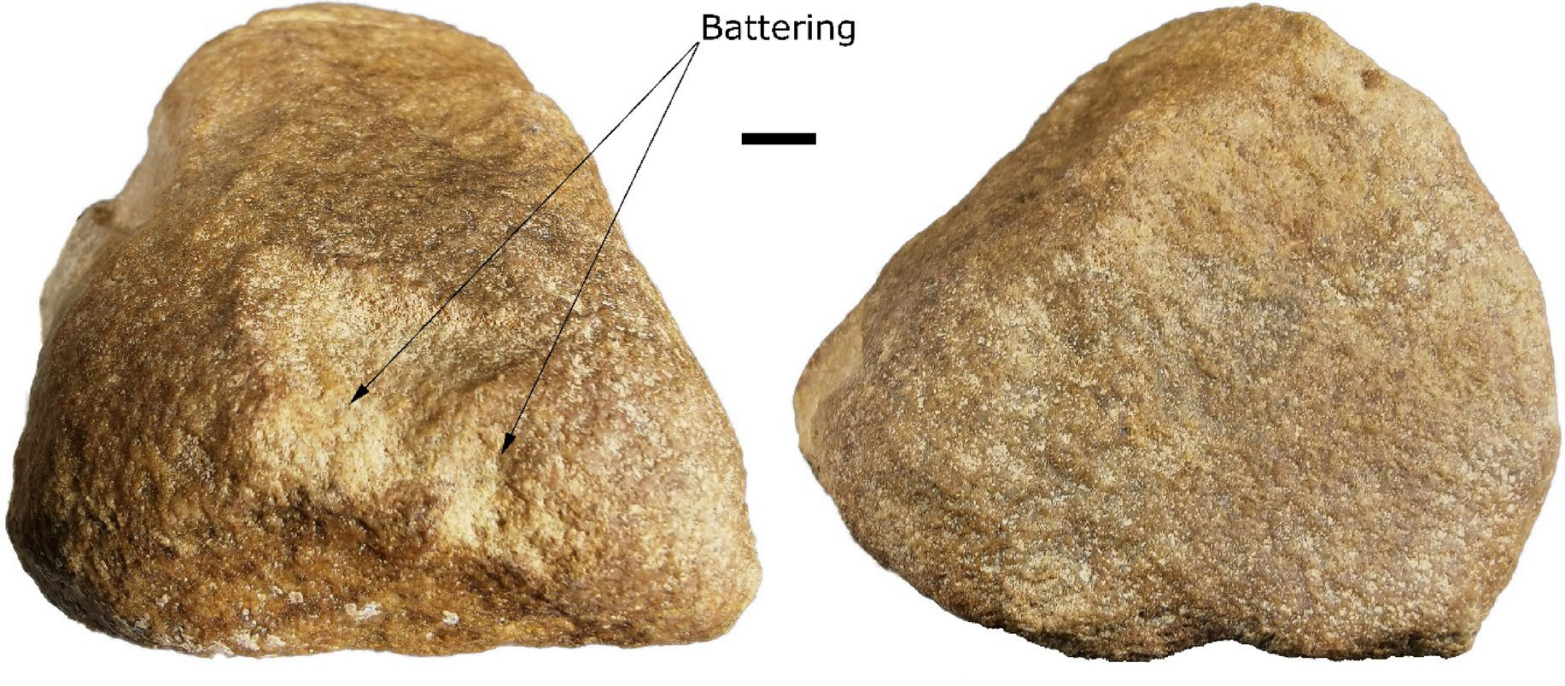
Quartzite pecking stone from SAU2 T1B layer 6. Note the wedge-shaped form of the piece and the battering damage at the narrow end. Scale is 1 cm.

Two artefacts interpreted as pecking stones for the creation of engraved rock art were found at Sahout. One was a ferruginous piece found on the surface at SAU1, directly below panel SAU1_B (Supplementary Fig. 29). The other was a quartzite piece found in layer 6 of the T1B excavation at SAU2 (Fig. 6). Both pieces are non-local tough material, with both having a wedge-shaped morphology and battering on their narrow ends, consistent with artefacts interpreted as pecking stones from Jebel Arnaan and Jebel Misma^20^. These artefacts fit comfortably in the hand in a four-jaw chuck grip, with the narrow end-protruding well beyond the fingers, and the broad end braced against the palm (Supplementary Fig. 30).

The recovery of the pecking stone from T1B layer 6 suggests some of the petroglyphs are contemporary with the deposition of that layer which was dated to 9100 − 8700 BP. The closest petroglyph to T1B is camel engraving SAU2_D, which is located in a narrow gap in the boulder (Fig. 5).

## Discussion

Here we synthesize the rock art and various lines of archaeological data to construct a regional prehistory for Sahout and the neighbouring sites of Jebel Arnaan and Jebel Misma. We then situate this within the broader Southwest Asian Terminal Pleistocene and early Holocene context.

**Fig. 7:**
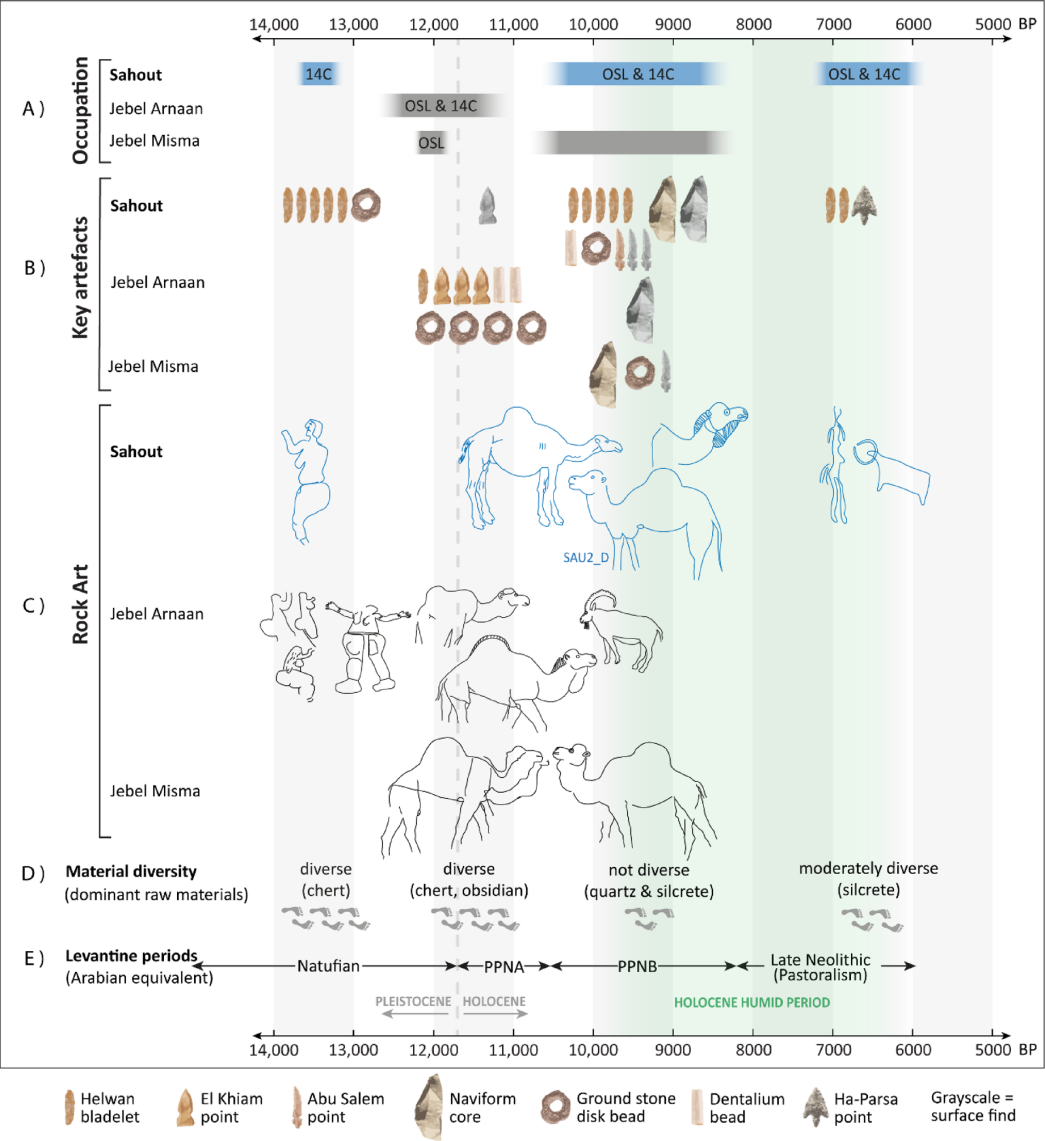
Synthesis of the archaeological and rock art record of the Sahout region and correlation with Levantine cultural periods. Sahout dating and rock art presented in this paper shown in blue. (**A**) Occupation phases based on 14 C and OSL dating, and artefact assemblages; (**B**) Key artefact types showing number of pieces from stratified contexts in colour and surface finds in greyscale; (**C**) Rock art sequence based on superimpositions and dated via engraving tools at ARN^20^and SAU, date ranges are approximate; (**D**) Footprints indicate high (6), medium (4), and low mobility (3) as inferred by diversity and sourcing of lithic materials; (**E**) corresponding periods in the Levant.

The transition to the Neolithic at Sahout and neighbouring sites appears to involve four distinct stages in human mobility strategies (Fig. 7) that are broadly contemporary with the Natufian, the PPNA, the PPNB, and the later Neolithic, spanning the Terminal Pleistocene through to the Holocene humid period. The presence of gazelle bones along with projectile points suggest the site was used as a hunting camp for most of its occupation. In the later PPNB the predominance of grinding stones and a sickle blade suggest cereal processing, while the presence of domesticated animals in later Neolithic layers and in the later rock art, suggests pastoralism. Throughout the occupation, petroglyph creation seems to have occurred alongside these subsistence activities.

The 13.5 ka date for phase 1 at Sahout coupled with the Helwan bladelets from those layers indicates this was an occupation contemporary to, and in contact with, Natufian communities in the Levant. Recent research at nearby palaeolakes has shown that this occupation dates to a period of comparatively arid conditions, but coincides with the earliest evidence of surface water availability in the region, following the hyper arid conditions of the Last Glacial Maximum^20^. Lithic materials in phase 1 show high diversity and the frequent use of non-local chert and chalcedony, indicating high day-to-day foraging mobility. In high mobility conditions the proportion of retouch will tend to be higher because tools need resharpening and/or to maintain compound tools replaceable inserts of standardized shape, such as Helwan bladelets, are created through retouch^30^. Levels of retouch in phase 1 are three times greater than either subsequent phase. Use-wear chips on the edges opposite the backing are evident on all Helwan bladelets, while the end-scrapers from phase 1 show edge rounding, consistent with a long use-life for both these tool types in high mobility conditions (Fig. 3 & Supplementary Figs. 19 & 23). The Khaybar obsidian source, ca. 190 km to the south, appears to have been used from this early stage, but this material was only occasionally transported to Sahout.

The engraved motifs included several curvaceous women in profile. At both Sahout and Jebel Arnaan these female figures are overlain by life-sized naturalistic camels^20^ (Fig. 7). This suggests engravings of curvaceous women belong to an earlier phase of rock art creation than the life-sized naturalistic camels of the PPN. Given the circumstantial evidence of the phase 1 Terminal Pleistocene occupation preceding the PPN at Sahout, we hypothesize that the female figures belong to this Natufian phase (Fig. 7).

The discovery of a broken El Khiam point (Supplementary Fig. 25) suggests that the Sahout site was probably also used during the PPNA. PPNA occupation was documented at Jebel Arnaan, ca. 20 km south-west, dated to around 12 ka^20^. At Jebel Arnaan a high diversity of materials suggests high day-to-day mobility. Although the site is approximately the same distance away from Jebel Al-Abyad as Sahout (Fig. 1), obsidian was a mainstay of lithic provisioning at Jebel Arnaan but it is rare at Sahout. Together with the two *Dentalium* shell beads recovered from the Jebel Arnaan excavations this suggests recurrent long-distance connectivity in the PPNA. At Jebel Arnaan the production of naturalistic camel engravings can be dated to this period^20^.

The ~ 9 ka dates for layers 6 and 5 in T1B (phase 2), coupled with the Abu Salem point and naviform blade core, indicate that the main occupation documented in the Sahout excavations relates to the PPNB. There is a close link between the use of local silcrete and PPNB lithic types at Sahout, with the Abu Salem points and the naviform cores from the excavation and surface all made of silcrete. This is echoed by finds of silcrete naviform cores from Jebel Arnaan and Jebel Misma, as well as a silcrete Abu Salem point from Jebel Misma^20^ (Fig. 7). A dominance of local silcrete in the lithics, alongside low overall material diversity, low proportions of retouch, and a high frequency of cumbersome grindstones suggest relatively low day-to-day mobility. However, during this PPNB occupation the Jebel Al Abyad obsidian source was still used, with pene-contemporaneous human occupation in the Umm Jirsan lava tube cave^31^which is just 22 km west of the volcano. At Sahout, long-distance connections (> 100 km) in the PPNB are confirmed by the *Dentalium* shell bead from the phase 2 occupation. *Dentalium* beads are known from the Upper Palaeolithic to the early Bronze Age in the Levant, but are only found in PPNB sites among more mobile groups in desert regions^32^.

The find of an in situ pecking stone in phase 2, of the same large wedge-shaped morphology as has been documented beneath a panel of life-sized naturalistic camels at Jebel Arnaan, confirms that petroglyph creation in Sahout took place during the PPNB. Life-sized naturalistic camels and wild asses dominate the panels at Sahout^24^(Fig. 5). We attribute this style of rock art to the phase 2 main occupation at SAU2, which also accounts for 64% of the lithic assemblage. These new results show a longevity of this tradition from the PPNA occupation at Jebel Arnaan, to the PPNB sequence at Sahout (Fig. 7). The long timespan of these monumental engravings had previously been supposed based on the frequency and extent of re-engraving events observed at Jebel Misma, at the Camel Site, and in Al Ula^20,33,34^. Moreover, there is a repeated sequence at Jebel Arnaan and Jebel Misma of naturalistic camel engravings, superimposed by camel engravings with round eyes and standardized body shape (Supplementary Figs. 33–34), with both these styles also represented at Sahout. This stylistic evolution can now be linked with dated archaeological deposits at Sahout, Jebel Arnaan, and Jebel Misma, providing a framework for this phase of petroglyph creation from the PPNA to PPNB, spanning ~ 3000 years (Fig. 7).

The stratigraphic marker linking both trenches in the SAU2 excavations was the rubble layer (layer 4 in T1B and layer 3 in T2B). Clast-rich layers were also encountered sealing the main occupation in trench 1 at Jebel Arnaan and contemporary with the main occupation at Jebel Misma locality 7^20^, suggesting they are part of a broader regional phenomenon. The 8.7 ka date for the rubble layer from the T1A excavation suggests this event may be contemporary with the peak of the Holocene humid period, dated 8.8 to 7.9 ka in north-western Arabia^35^, with the change in humidity causing enhanced sandstone weathering. Broken tangs consistent with Byblos points from the rubble layers at SAU2 accord with the late PPNB date indicated by associated radiocarbon ages (Figs. 2 and 7; Supplementary Fig. 19).

Dates of 6.4 ka from SAU2 T2B, and 6.9, 6.1, and 5.9 ka from hearth features at SAU2 and SAU4, indicate occupation during the later Holocene humid period. A Ha-Parsa point from phase 3 accords with a later Neolithic attribution (Fig. 7), with Ha-Parsa points found in contemporary sites on the Harrat ‘Uwayrid to the west of the Nefud^13^and around Jubbah within the Nefud^17,21^. The *Bos* remains from this phase testify to wetter conditions at the time. Together with remains of *Ovis/Capra* sp., these may reflect the introduction of livestock. This change in fauna is also visible in the rock art at Sahout. Motifs such as elongated human figures and bovids with exaggerated horns are well known from the Neolithic rock art sites of Jubbah and Shuwaymis^23,36 ^and were also documented at SAU1 and SAU2^24^. These images likely correspond to the phase 3 later Neolithic occupation at SAU2, and the presence of cattle in this rock art accords with the *Bos* remains from this phase. In the later Neolithic, higher material diversity suggests higher day-to-day mobility than the PPNB, perhaps with the adoption of pastoralism.

The stratigraphic sequence excavated at Sahout, and the substantial lithic assemblage, now allow a more detailed reconstruction of the population history of the Terminal Pleistocene and early Holocene in Southwest Asia. The earliest occupation at Sahout shares stone tool types and is contemporary with the Natufian; yet Sahout is over 480 km farther south-east than the most southerly known Natufian site at Wadi Judayid in southern Jordan^37^. That the next oldest diagnostic lithics at Sahout and elsewhere in northern Arabia are Middle rather than Upper Palaeolithic, suggests that Natufian populations contributed to the initial repopulation of northern Arabia after the hyper-aridity of the Last Glacial Maximum. Ancient DNA evidence from Medieval Soqotra also indicates that Natufians were a founding population of recent Arabian peoples^38^. This hitherto unappreciated geographic scale of Natufian stone tools supports the hypothesis that population expansion was an important precursor of the transition to farming^39^, while overturning the conception of these communities as exclusively sedentary and absent from marginal landscapes^40,41^.

Repeated occurrence of lithic types from the Fertile Crescent at Sahout indicates sustained long-distance connectivity. Connections over hundreds of kilometres are a notable trend elsewhere in the Epipalaeolithic of southwest Asia^42,43^. Isotopic analyses from the southern Levant and southern Anatolia indicate frequent long-distance movements of people in the Natufian and early PPN, then a decline in the late PPNB^44,45^. Regular long-distance connectivity has been suggested to be an important component of cultural transformation and intensification^46^, including specifically in the PPN^47^, through the exchange and combination of disparate people and ideas. At Sahout, Jebel Arnaan, and Jebel Misma, long distance connectivity is evident in the exotic obsidian, *Dentalium* beads, and Levantine lithic types. These Levantine connections are coupled with high day-to-day mobility during the arid periods contemporary with the Natufian and the PPNA. However, when day-to-day mobility decreases during the PPNB, perhaps with the greater availability of water and vegetation after the onset of the Holocene humid period (Fig. 7), the long-distance connectivity persists. Obsidian movements between southern Anatolia and the Levant similarly show that despite an increase in sedentism between the PPNA and the PPNB, long-distance material exchanges were enhanced^48^. The lithics indicate such connections were more than the exchange of exotic items and copying of end-products; the complete Abu Salem point production sequence is present at Sahout from the initial cresting blades, through the naviform cores, to the pressure flaking of the blades into points. The life-sized naturalistic camel rock art tradition evident at Sahout is also found on the other side of the Nefud, with a parallel stylistic evolution indicating connectivity across the desert, perhaps even including the same individual engravers^49^. While day-to-day mobility decreased in the PPNB, we hypothesize that seasonal movements across the Nefud were maintained.

The discovery of substantial Terminal Pleistocene and early Holocene communities at Sahout shows that the interaction spheres which facilitated the emergence of farming in the Fertile Crescent were much more extensive than previously thought, incorporating communities hundreds of kilometres to the south in the Arabian desert.

## Methods

### Fieldwork

Comprehensive surface survey was conducted on foot across the Sahout site to identify petroglyphs, surface exposures of knapping material and lithics, and hearth features. The lithic scatter at the silcrete exposure (SAU4) was sampled through collection of technologically diagnostic artefacts and comprehensive collection of all artefacts within one square metre in the centre of the scatter.

Trenches were excavated with trowels using the single context method^50^, with contexts recorded on standardized forms. Contexts depths were recorded with a line-level, with five points taken for each context. Intrusive sediment contexts (e.g. termite galleries and rodent burrows) were emptied and discarded though in cases of long infilled intrusions their identification was unreliable. Excavated sediment was sieved through a 3 mm mesh with all artefacts and faunal remains retained.

### Dating

Samples for luminescence dating were taken by hammering metal tubes into cleaned excavation sections, with opaque material inserted into the leading end upon extraction. These were exported to Royal Holloway University, UK, and analysed as described in Supplementary Note 2. Environmental dose rates were calculated using location and overburden density (cosmic rays), field gamma spectrometry (gamma), and thick-source beta counting (beta).

Radiocarbon samples of charcoal and bone were collected during excavation. Samples were sent to the Centre for Applied Isotope Studies (CAIS) at the University of Georgia, USA, and analysed as described in Supplementary Note 3.

### Fauna

All bone was exported and identified to the lowest taxonomic level possible with the help of comparative material housed at Griffith University, Australia, and online resources. All specimens were assessed for taphonomic features such as weathering, fracture patterns, and burning following established protocols^51–53^. Results are reported as number of specimens (NSP) and number of identified specimens (NISP)^54^.

### Artefacts

Artefacts were exported to University College London, UK. All excavated and surface collected lithics were classified by material, then counted and weighed. Retouched pieces and cores were assigned to types. Key technologically or culturally diagnostic types were photographed. Grinding stones were assigned to types, their ground surface form characterized, production techniques noted, and any complete dimensions measured. Pigment was weighed and pieces with striations visible at low magnifications designated as crayons. Beads were assigned to types and photographed. Statistical tests were performed in Microsoft Excel.

### Geochemical provenancing

Obsidian lithics > 1.5 cm in maximum dimension were analysed semi-quantitatively by portable X-Ray Fluorescence analysis (pXRF) in the Wolfson Laboratory at the UCL Institute of Archaeology. An Olympus Vanta was used to take the readings with a proprietary factory calibration model. We used geochemistry 3 beam mode and a 30 s livetime count, with beam 1 using a 2 mm Aluminium filter @ 40 kV, beam 2 open (no filter) @ 10 kV, and beam 3 using a 350 μm copper filter @50 kV.

Element concentrations of manganese (Mn), iron (Fe), zinc (Zn), gallium (Ga), rubidium (Rb), strontium (Sr), yttrium (Y), zirconium (Zr), and niobium (Nb) and thorium (Th) were calculated. Elemental concentrations were compared to four known obsidian sources in Central Anatolia (Bingöl A and B, Nemrut Dag, Mescrut Koavk)^55^, ten obsidian sources from the southern Arabian Peninsula (Jebel Lisi, Jebel Isbil 1 and 2, Al-Gharga, Jirab al Souf, Hayd al Halal 1 and 2, Yafa’ Ridge 1, 2 and 3 and Afar 1)^56^ and two identified sources close to the archaeological site (Jabal Al-Abyad 1 and 2)^57^. Additional source samples collected from the Jabal Al-Abyad volcano at 25°40’01.2”N 39°58’10.4”E were also included in the PXRF analysis.

Discriminant Analysis in the PAST4 software was used for comparison. Geochemical analysis of 24 obsidian artefacts was conducted with the Discriminant function analysis plotting the elemental composition of each artefact against the reference samples.

### Rock art

Rock art panels were documented using standardized forms, recording content, size, and GPS location. Distinct surfaces of boulders were defined as separate panels, with multiple surfaces on the same boulder labelled with the same number, distinguished by a letter suffix. Panels were recorded using high-resolution digital photographs as raw files and jpg. To enhance faded details on the rock art some photos were enhanced using Adobe Lightroom. On key panels, overlapping photographs from different angles were used to generate high-resolution 3D models and orthophotos using Metashape photogrammetry software.

## Supplementary Information

Below is the link to the electronic supplementary material.

Supplementary Material 1

## Data Availability

All data used in this study are available in the Supplementary Information file. All data used to support our results was generated in our research.
