# Supplementary material for: The Natufian Epipalaeolithic and Pre-Pottery Neolithic in the desert of northern Arabia
Source: Sci Rep. 2026 Mar 2;16:16842. doi: 10.1038/s41598-026-40541-3 (PMC13226711; doi:10.1038/s41598-026-40541-3)
Supplement: Supplementary file 1 — Supplementary Material 1 [file 41598_2026_40541_MOESM1_ESM.docx]

**Supplementary Notes for:** **The Natufian Epipalaeolithic and Pre-Pottery Neolithic in the desert of northern Arabia**

Ceri Shipton*^1,2^, Maria Guagnin*^3,4^, Faisal Al-Jibreen^5^, Finn Stileman^6^, Mathew Stewart^7^, Simon J. Armitage^8,9^, Nick Drake^10^, Christian Reepmeyer^11^, Paul S. Breeze^10,12^, Frans van Buchem^12^, Fahad Al-Tamimi^5^, Muhammed Al-Shamry^5^, Ahmed Al-Shammari^5^, Jaber Al-Wadani^5^, James Blinkhorn^13^, Abdullah M. Alsharekh^14^, Michael Petraglia^7,15,16^

^1^ Institute of Archaeology, University College London, London, UK

^2^College of Asia and the Pacific, Australian National University, Canberra, Australia

^3^ Department of Archaeology, Max Planck Institute of Geoanthropology, Jena, Germany

^4^ School of Humanities, The University of Sydney, Sydney, Australia

^5^ Heritage Commission, Saudi Ministry of Culture, Riyadh, Saudi Arabia

^6^ Department of Archaeology, University of Cambridge, Cambridge, UK

^7^ Australian Research Centre for Human Evolution, Griffith University, Brisbane, Australia

^8^ Department of Geography, Royal Holloway University of London, Egham, UK

^9^ SFF Centre for Early Sapiens Behaviour (SapienCE), University of Bergen, Post Box 7805, 5020, Bergen, Norway

^10^ Department of Geography, King’s College London, London, UK

^11^ Commission for Archaeology of Non-European Cultures, German Archaeological Institute, Dürenstrasse 35-37, 53173 Bonn-Bad Godesberg, Germany

^12^ Physical Science and Engineering Division, King Abdullah University of Science and Technology (KAUST), Thuwal, Saudi Arabia

^13^ Department of Archaeology, Classics and Egyptology, University of Liverpool, Liverpool, UK

^14^ Department of Archaeology, College of Tourism and Antiquities, King Saud University, Riyadh, Saudi Arabia

^15^ Human Origins Program, Smithsonian Institution, Washington, D.C., USA

^16^ School of Social Science, University of Queensland, Brisbane, Australia

*Corresponding authors

**Supplementary Note 1: Fieldwork**

The Sahout site occurs on the Palaeozoic Saq sandstone formation at an altitude of 936 m. Today this region is hyper arid^1^.

A dense concentration of lithics was noted on the surface around a silcrete outcrop at SAU4 (Fig. 1). The silcrete probably originally formed as precipitate on the sandstone, under endorheic lacustrine conditions^2^. A systematic surface collection of artefacts within 1 m^2^ was undertaken in the densest part of the silcrete lithic concentration (Supplementary Figure 1). A total of 429 lithics were in the scatter, all but two of which were silcrete. In keeping with this being a workshop locality, seven of the lithics were cores (1.6%), with many flakes produced and discarded from each core, and only 3 lithics (0.7%) being retouched, with the finished tools exported.


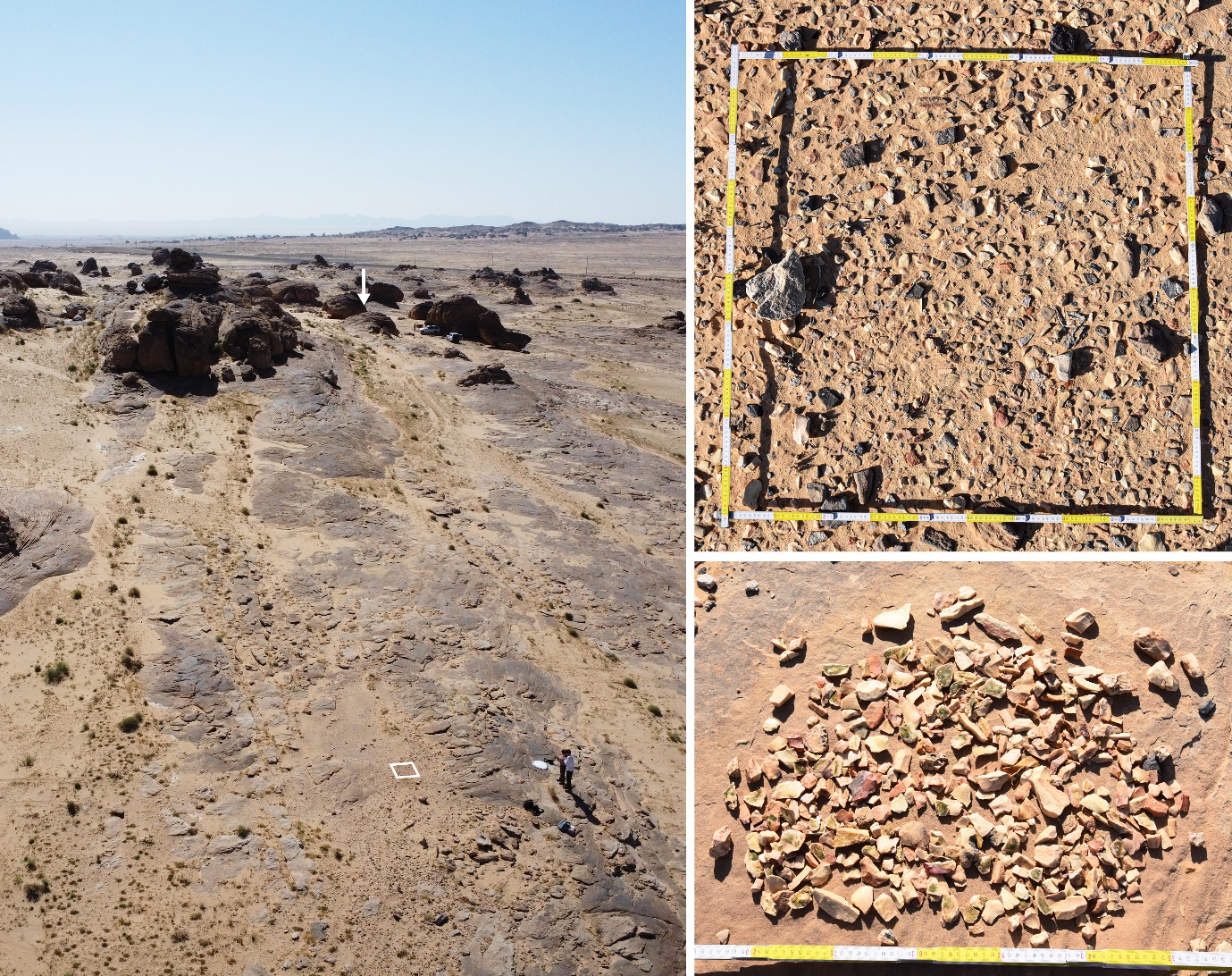


*Supplementary Figure 1. The 1 m^2^ systematic surface collection at SAU4_2. Left shows the location of the square looking east with the SAU2 locality indicated by the white arrow in the background. Team members in the foreground, and vehicles to the right of the arrow provide a scale. Top right shows the square prior to collection and bottom right shows all the lithics that were within the square.*

Two trenches were excavated at Sahout locality SAU2, laid out to encompass the previous two test excavations. SAU2 Trench 1B (T1B) was a 1x1.5 m trench aligned to the front of the middle of the boulder face, while Trench 2B (T2B) was a 1x1 m trench situated ~1.5m to the east but separated by a buried boulder, and tucked into a niche behind a small rock formation in front of the main boulder (Supplementary Figures 2 and 3). The test trenches (T1A and T2A) were emptied prior to the excavation of the new trenches (Supplementary Figure 3). During excavation, both recent and ancient termite burrows were encountered, these were emptied and the contents discarded when detected (Supplementary Figures 4 and 5). However, in some cases ancient burrows were difficult to identify as the fill had become similar to the surrounding contexts (Supplementary Figure 6).

While the T1B sequence sat on sterile sand, T2B was underlain by slabs of sandstone; a noteworthy difference between the two trenches given their proximity (Supplementary Figure 8). In contrast to the well-sorted loose sand from the upper layers, the rest of the sequence in both trenches showed greater compaction, increased moisture content, and greater variation in grain size.


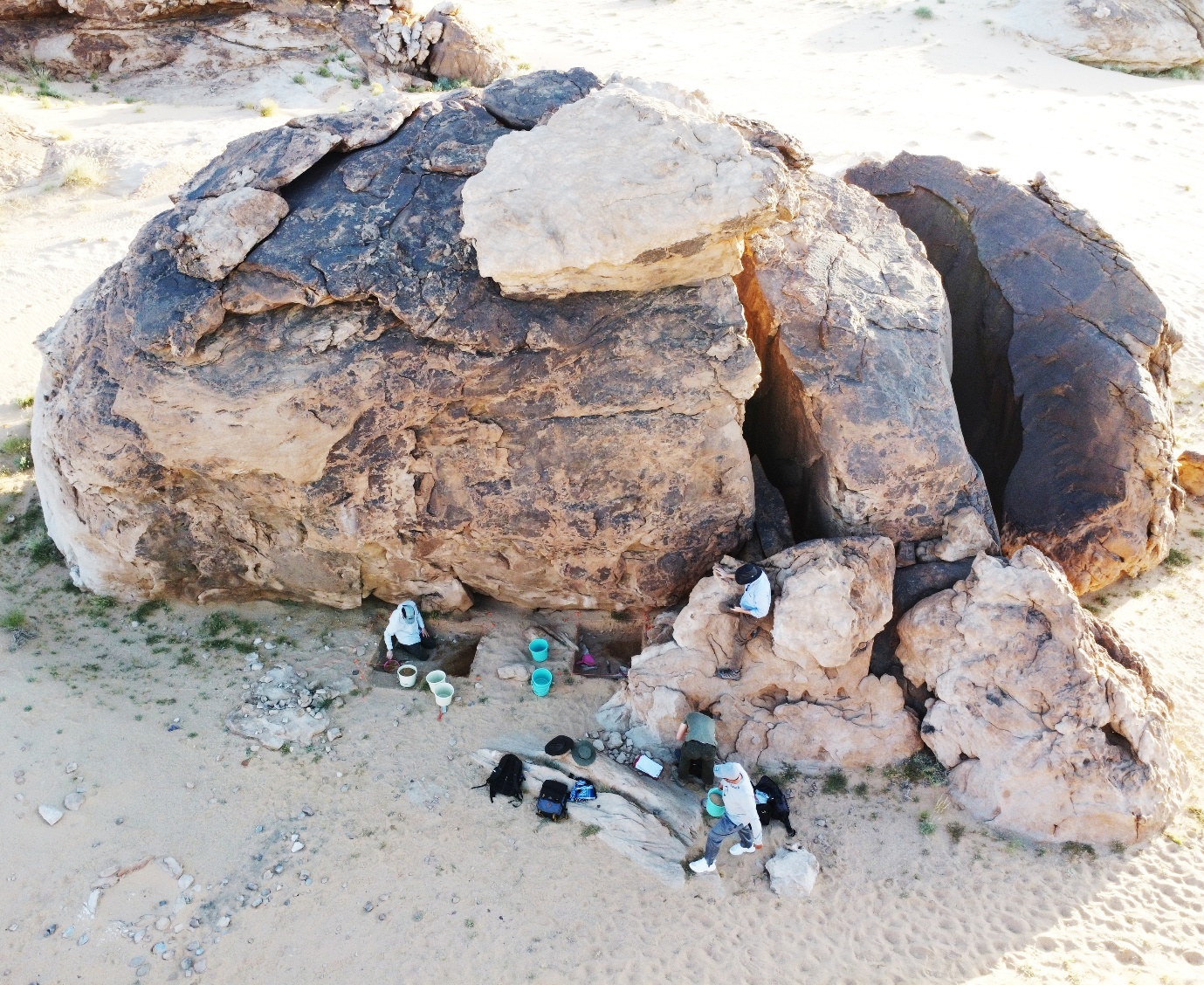


*Supplementary Figure 2. Overview of the SAU2 boulder and T1B (left) and T2B (right) excavations. Note the position of T2B protected by a rock formation in front of the main boulder.*


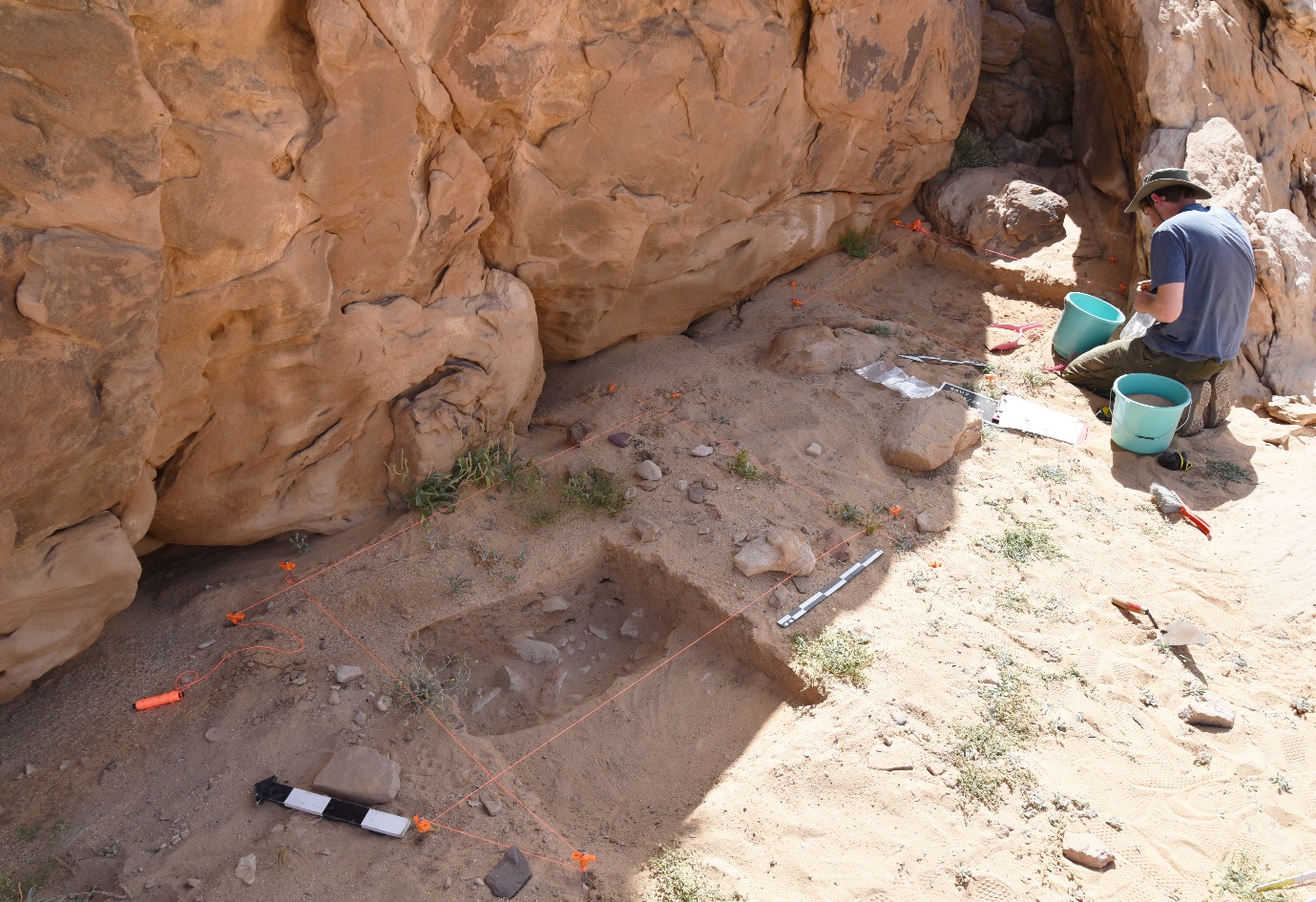


*Supplementary Figure 3. SAU2 T1B prior to excavation (left) and SAU2 T2B at the beginning of excavation (right). Note the previous test excavation emptied out with its deepest part within T1B. T2B sits in a small alcove between the main boulder and another rock formation, while a partially buried boulder lies between the two trenches on the western edge of T2B.*

*
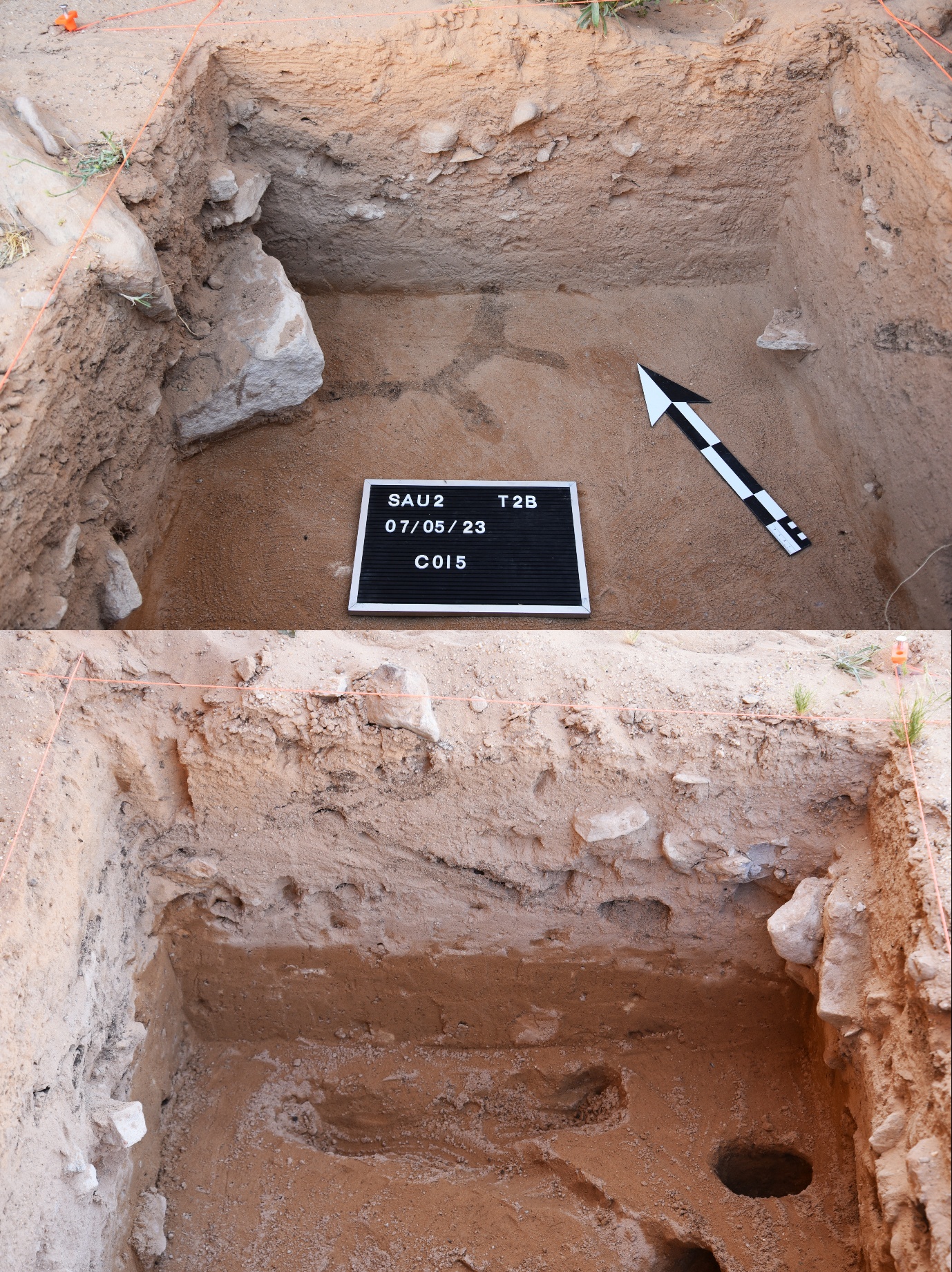
*

*Supplementary Figure 4. Termite galleries in the SAU2 excavations. Above: Gallery prior to being emptied during the excavation of T2B layer 5. Below: galleries after being emptied during the excavation of T1B layer 6. Note the moisture retention in the recently excavated layers, particularly in T1B. Scale is 40 cm.*

*SAU2 Trench 1B*

In T1B eight horizontally bedded layers were differentiated (Supplementary Figure 5), with knapped stone artefacts in all but the lowest. Layer 8 was a culturally sterile friable mid brownish orange sand, mottled with brownish red coarse poorly sorted sand with weathered sandstone inclusions. Both the sand and the sandstone clasts likely derive from the sandstone rockshelter wall which is constituted of poorly sorted grains. Layer 7 was a poorly sorted sand containing only 10 artefacts, with horizontally bedded weathered sandstone in the top of this layer (Supplementary Figures 5 & 6) suggesting it was a stable surface representing the transition between the sterile below and the archaeological sequence above. Layers 6 and 5 were coarse sands with much higher artefact concentrations of 115 and 376 lithics respectively. Layer 6 was a moist poorly sorted sand, with occasional pieces of degrading sandstone. A large lithic was found under the big rock in the west section of this layer (Supplementary Figure 5). Layer 5 was a moist firm coarse sand, with occasional small sandstone clasts up to 5 cm in maximum dimension. Layer 4 had very frequent sandstone clast inclusions up to 20 cm long in a matrix of medium sand (Supplementary Figure 5), with the highest artefact concentration of 685 lithics. Layers 3 and 2 were firm silty sands containing 132 and 83 lithics, respectively. Firmness and moisture retention of layer 2 and below was due to the increased finer sediment component (Supplementary Figure 4). The moisture content is probably also what attracted termites to these layers (Supplementary Figure 4). Layer 1 was a well-sorted coarse sand that appears to be of more aeolian origin than the layers below, and contained 64 lithics. This top layer included vegetation, sandstone clasts, and occasional charcoal inclusions, the latter the likely source for the historical contamination noted in some of the layers below.


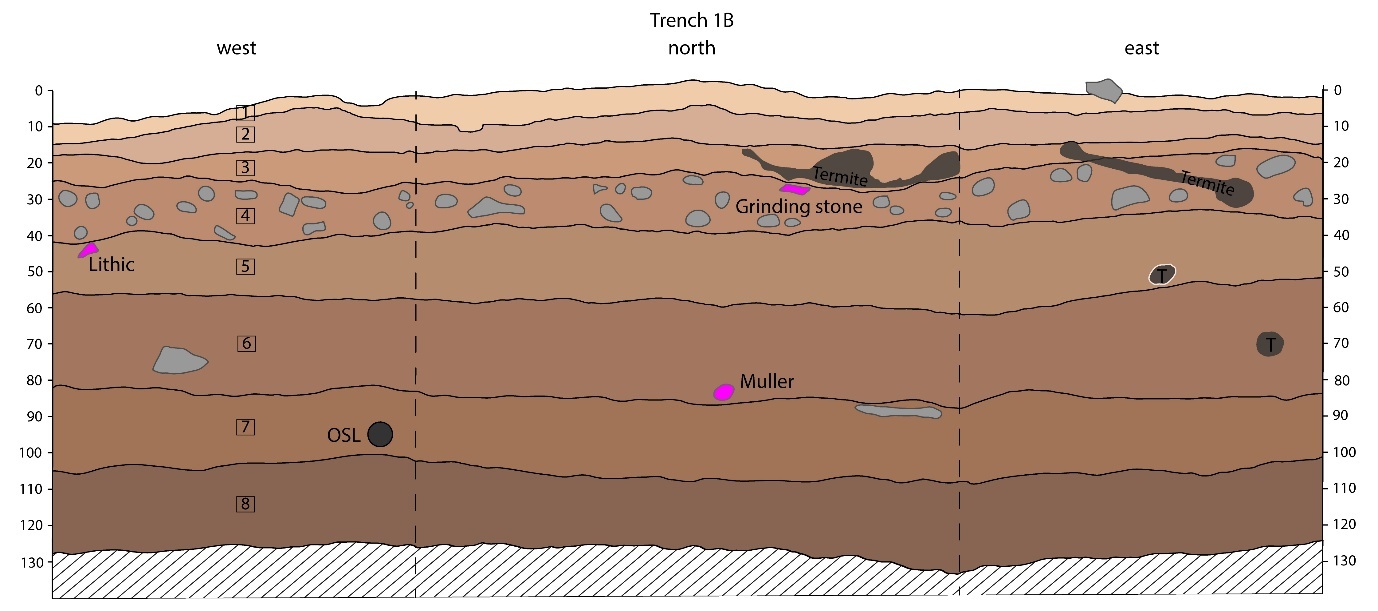


*Supplementary Figure 5. West, north, and east sections of SAU2 T1B, showing the 8 layers. The termite gallery outlined in white is an ancient burrow that was not identified during excavation. Large artefacts are shown in pink. Location of the OSL sample is shown with a black circle. Note the large horizontally bedded clast at the top of layer 7. Layer 8 did not contain artefacts. Hatching indicates unexcavated natural deposit. Vertical scale in cm.*

*
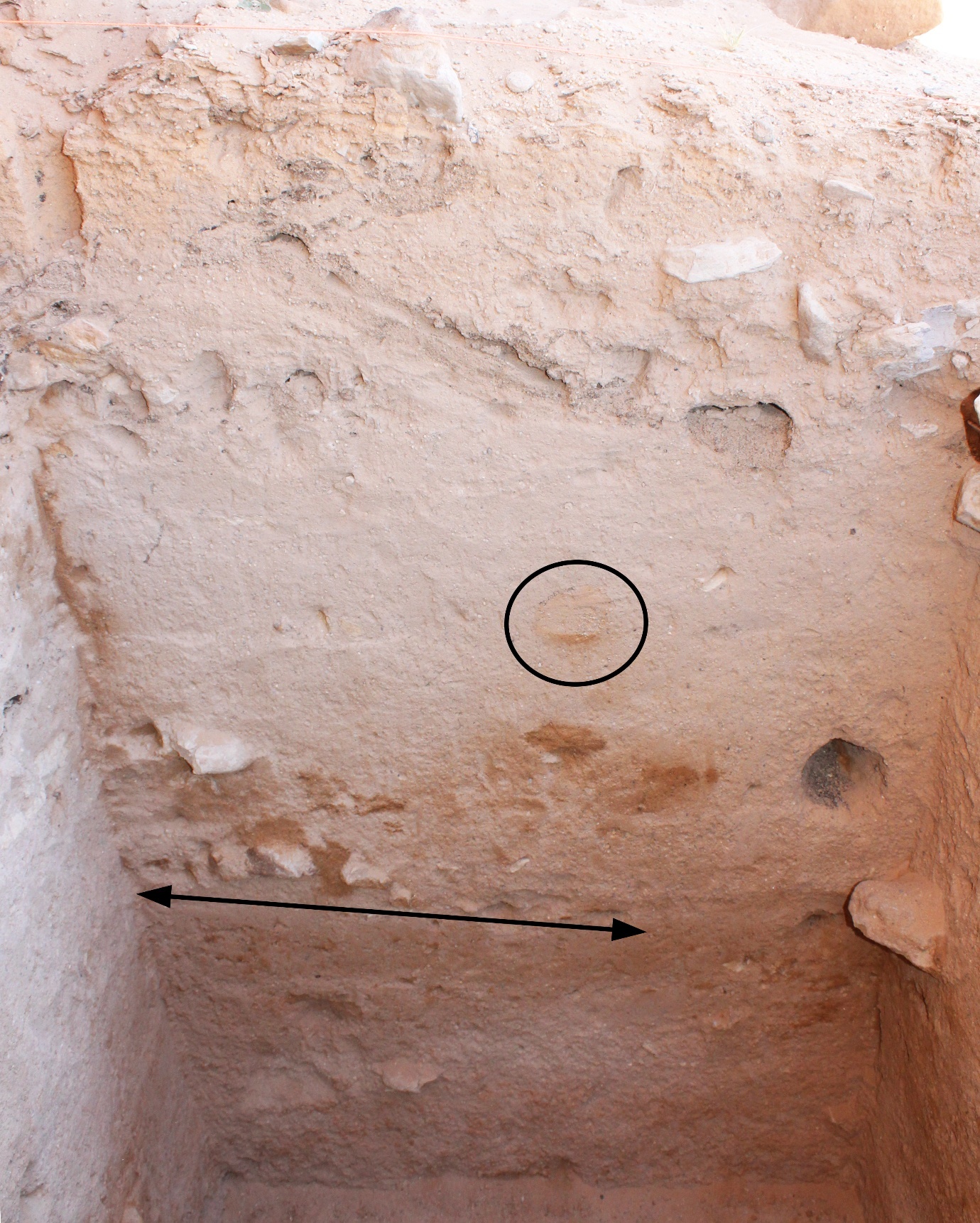
*

*Supplementary Figure 6. The east wall of T1B at the end of excavation. The ellipse denotes an old termite burrow in layer 5 and the double ended arrow denotes the horizontally bedded degrading sandstone at the top of layer 7.*

*SAU2 Trench 2B*

Six layers were differentiated in T2B (Supplementary Figure 7) with knapped stone artefacts in all but the lowest. The base of the excavation was large slabs of sandstone with bedding dipping sloping southwards, unlike the horizontally bedded sand at the base of T1B (Supplementary Figure 8). Layer 6 was a pale sand with moderate sandstone clasts and was archaeologically sterile. Layer 5 was a poorly sorted sand with occasional sandstone clasts and 28 lithics. Termite galleries were visible in this layer (Supplementary Figure 4). Layer 4 was a fine sand with occasional large sandstone clasts and the highest concentration for the trench of 180 lithics. The top of layer 4 represents the lower limit of the test excavation of T2A. Layer 3 consisted of dense sandstone clasts in a fine sand matrix with 43 lithics, and is equivalent to layer 4 in Trench 1 (Supplementary Figure 7). Layer 2 had a high density of 140 lithics in a poorly sorted firm sand matrix with sandstone clasts. Layer 1 was well-sorted sand likely of aeolian origin as in T1B, with 40 lithics.


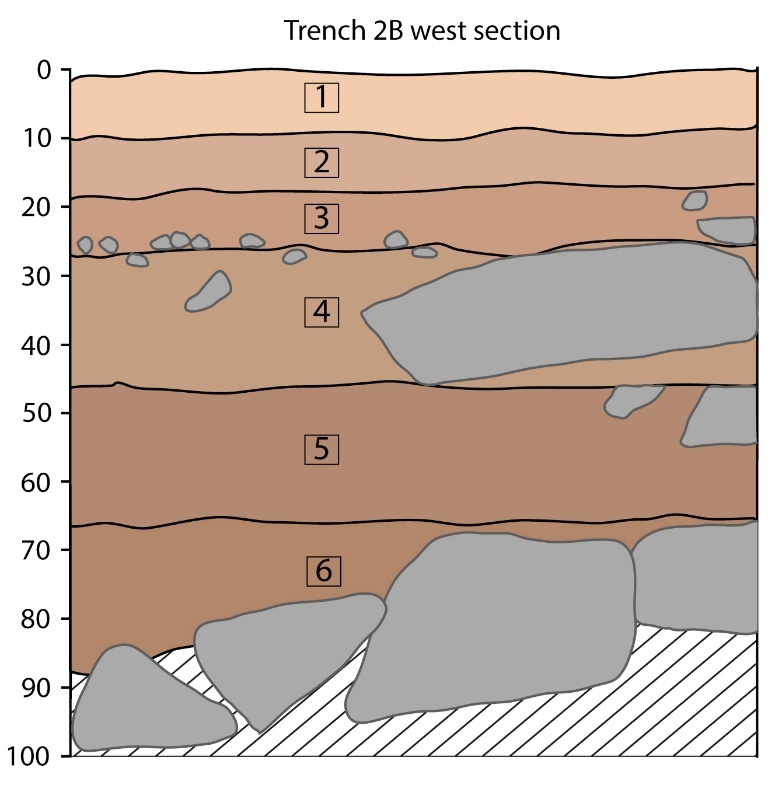


*Supplementary Figure 7. West section of SAU T2B showing the 6 layers. Hatching indicates unexcavated natural deposit. Layer 6 did not contain artefacts. Vertical scale in cm.*


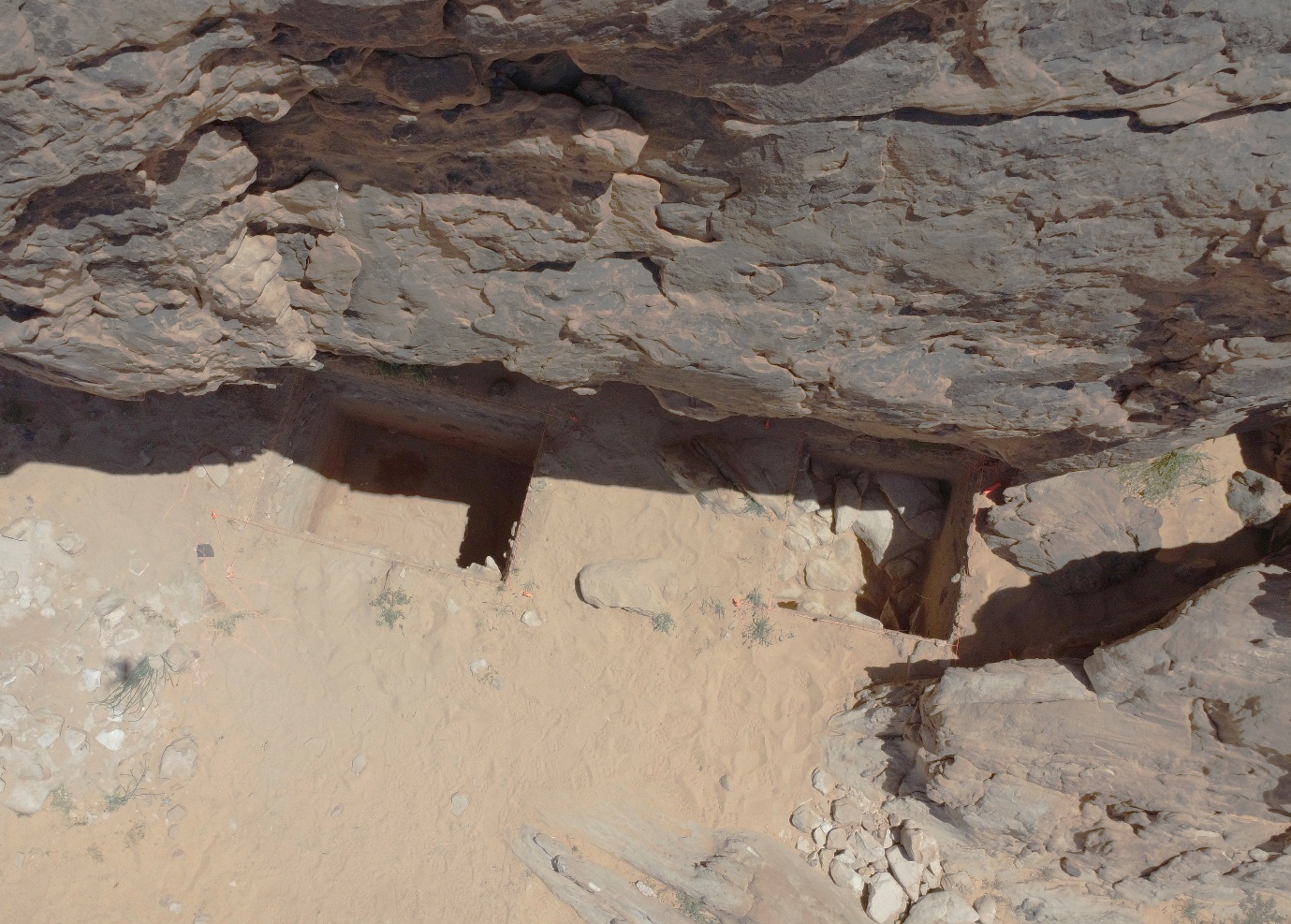


*Supplementary Figure 8. SAU2 T1B (left) and T2B (right) at the end of excavation. Note the sandy base of T1B and the rocky base of T2B.*

*SAU4_6 Hearth Excavation*

A stone lined hearth was half-sectioned at SAU4_6 (Supplementary Figure 9). Four fills were identified in the cut for the hearth. The top fill was a loose aeolian sand ~1 cm thick. The second fill was a compact brown sand ~10 cm thick with occasional sandstone pieces up to 15 cm in maximum dimension. The central fill was an ashy sand ~13 cm thick with frequent burnt sandstone clasts. Charcoal from this fill was sampled for radiocarbon dating. The lower fill was a fine dark grey ashy sand c. 10 cm thick with occasional small pieces of sandstone. The cut of the hearth had vertical sides and a flat bottom, measuring 34 cm deep. It was elongate oval in plan and lined with a double layer of sandstone slabs each ~4 cm thick.


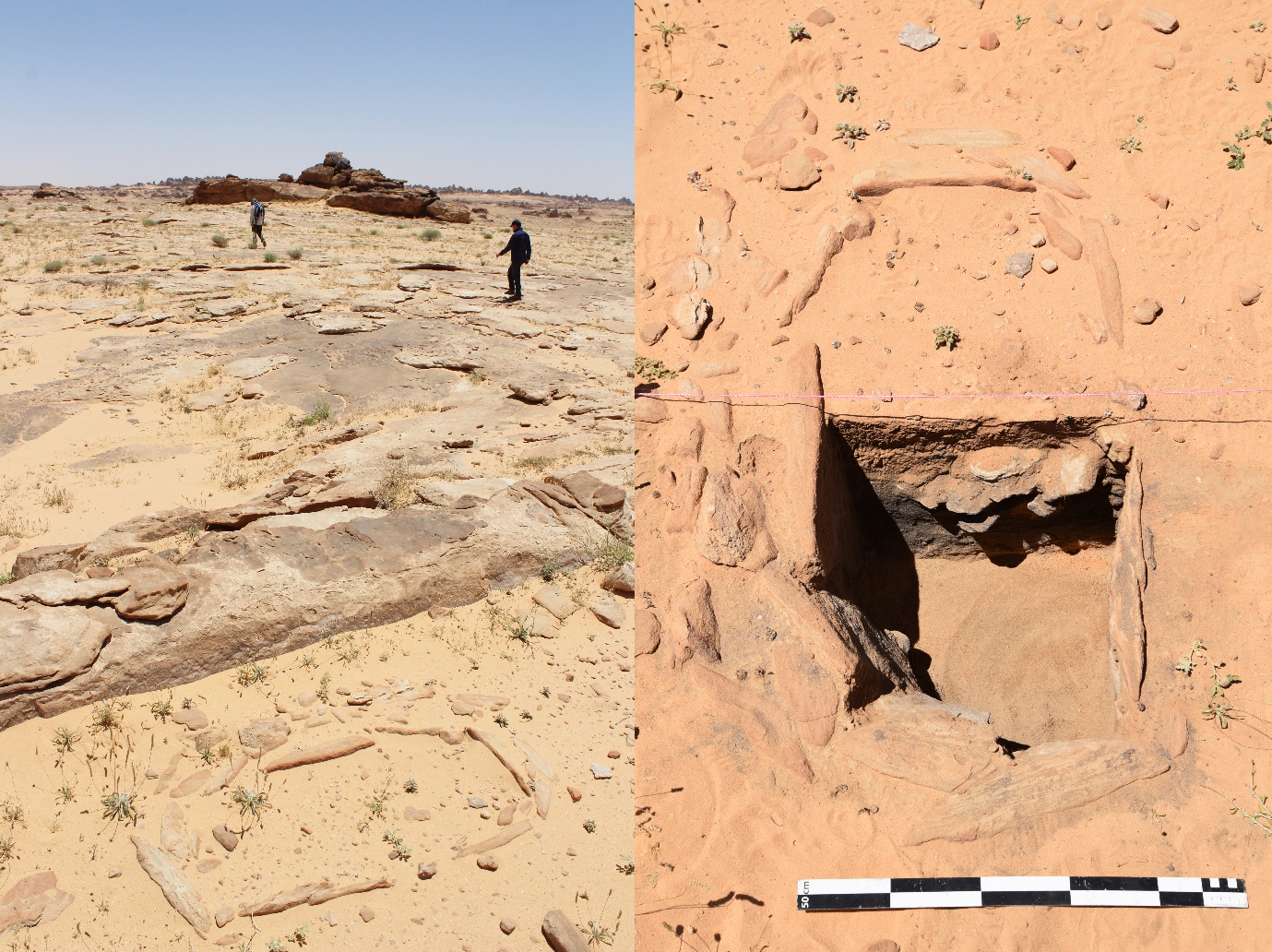


*Supplementary Figure 9. Stone-lined hearth SAU4_6 prior to (left) and after half-section excavation (right). Note the stones within the hearth as well as lining it.*

**Supplementary Note 2: Optically Stimulated Luminescence age estimate**

A sediment sample (T1B-OSL5) was taken by driving an opaque tube into the cleaned section face of T1B layer 7 (Supplementary Figure 5). The sample was prepared under subdued red-light conditions at Royal Holloway Luminescence Laboratory. The outer, light-exposed portions of the sample were removed and used for environmental dose rate measurements. The remaining sediment was treated with hydrochloric acid (1M HCl) and hydrogen peroxide (H_2_O_2_) to remove carbonate and organic matter respectively. The samples were wet sieved to yield 180-210 µm sand, and quartz was extracted via density separation at 2.62 and 2.70 g/cm^3^, and a subsequent HF acid etch (23M HF for 60 mins, followed by a 10M HCl rinse). Etched material was re-sieved to remove partially dissolved grains.

Luminescence measurements were performed using a Risø TL/OSL-DA-15 instrument, fitted with a single-grain luminescence attachment^3^. Stimulation of individual mineral grains was carried out using a 10 mW Nd: YVO_4_ solid-state diode-pumped green laser (532 nm) focussed to yield a nominal power density of 50 W/cm²^4^. Quartz luminescence emissions were detected using an Electron Tubes Ltd 9235QB photomultiplier tube via 7.5 mm of Hoya U-340. Irradiations were performed using a 1.48 GBq ^90^Sr/^90^Y beta source, calibrated relative to the National physical Laboratory, Teddington ^60^Co gamma-source (Hotspot 800)^5^. For single-grain measurements, the effects of source heterogeneity^6^ were circumvented by applying a grain position correction to each equivalent dose following^7^. Position corrected equivalent doses were used in all single-grain age calculations.

Equivalent doses were determined using the single-aliquot regenerative-dose protocol^8,9^ (Supplementary Table 1) applied to single-grains of quartz. Regeneration doses were chosen to bracket the expected palaeodose. A number of additional regeneration points were also included to monitor the quality of the data generated: (1) a zero-dose point to measure the recuperation; (2) a repeat measurement of the initial regeneration dose to calculate the recycling ratio^8^; (3) a second repeat of the initial regeneration dose followed by a room temperature IR bleach and subsequent OSL measurement to calculate the IR depletion ratio^10^. We adopted a combination of 260 °C for 10 s for preheat 1 (PH1, the preheat before measurement of the natural or regenerated luminescence intensity) and 220 °C for 10 s for PH2 (the preheat before measurement of the test dose luminescence intensity) for single-grain measurements (Supplementary Table 1). The dose response curves were fitted with a general order kinetic function. D_e_ values for individual grains were calculated by projecting the sensitivity-corrected natural luminescence intensity (L_n_/T_n_) onto the dose response curve, with an uncertainty term determined using a 1000 iteration Monte Carlo simulation. Curve fitting, D_e_ determination and Monte Carlo simulation were conducted on single-grain data using the function *calSARED()* provided in the R package *numOSL*^11,12^.

*Supplementary Table 1*. *The single-aliquot regenerative dose protocol used to measure individual grains of quartz in this study.*

| **Step** | **Quartz**  **Single-grain** |
| --- | --- |
| 1 | Give regenerative dose^1^ |
| 2 | Preheat 1  (260 ⁰C for 10 s) |
| 4 | Green laser stimulation  (125 °C for 2 s) |
| 5 | Give test dose  (~15 Gy) |
| 6 | Preheat 2  (220 °C for 10 s) |
| 8 | Green laser stimulation  (125 °C for 2 s) |
| 9 | Blue diode bleach  (280 ⁰C for 100 s) |
| 10 | Return to step 1 |

Grains were rejected where: (1) the natural signal from the grain could not be distinguished from the background signal (determined using a combination of “Tn below 3 sigma BG” and “RSE of Tn exceeds 10%” rejection criteria in *calSARED()*); (2) the recycling ratio differed from unity by >2 standard errors; (3) the sensitivity-corrected zero dose luminescence intensity was >5 % of the natural luminescence intensity; 4) the IR-depletion ratio was more than two standard errors below unity; (5) The L_x_/T_x_ ratios are too scattered to be reliably fitted with a curve (grains fail where the figure-of-merit value exceeds 10%, determined using *calSARED()*) and 6) the natural luminescence signal (L_n_/T_n_) intercepts the dose response curve at a point where growth has ceased. Very high grain rejection rates were noted for sample T1B-OSL5, with only 0.35% of grain positions measured yielding an acceptable D_e_ estimate (Supplementary Table 2). High rejection rates have been noted elsewhere in the Nefud^13^, but the yield of acceptable grains from T1B-OSL5 is anomalously low even by that standard. Using the instrument used in the present study, single-grain measurements on the Risø calibration sample yielded 78% grain acceptance versus a reported value of “~80%” in the literature^13^. We conclude that the instrument used in this study was set-up in an acceptable manner, and propose that the high rejection rate of grains resulted from a combination of low absorbed dose and low inherent sensitivity, exacerbated by the known poor luminescence properties of Nefud quartz.

Supplementary Table 2. The number of individual quartz grains which were measured, rejected and accepted during analysis of the single-grain data for sample T1B-OSL5.

|  | Rejection criterion (n) | | | | |  | Rejected | Accepted | Total |
| --- | --- | --- | --- | --- | --- | --- | --- | --- | --- |
|  | 1 | 2 | 3 | 4 | 5 | 6 | (n) | (n) | (n) |
| T1B-OSL5 | 11,722 | 28 | 2 | 120 | 80 | 0 | 11,958 | 42 | 12,000 |

The statistical models most frequently used to extract a single burial dose (D_b_) from complex distributions of measured D_e_ values is the Finite Mixture Model (FMM) ^14^. FMM was developed to identify separate, fully bleached populations of grains within a single-grain dataset. This model is used when the sample is a mixture of different grain populations, for example when mixing occurred after burial.

Because of the presence of low-dose (presumably intrusive) grains within the single-grain dataset, the FMM was used to identify the discrete grain populations. The FMM was fitted using overdispersion values of 10-26%, and an optimal fit at 26% overdispersion was determined from the maximum log-likelihood values and the Bayesian information criterion^15^. This yielded three populations, comprising 7%, 29%, and 64% of grains. The optimal number of components and resulting De values is stable between 21% and 26% overdispersion.

The total dose rate for an HF etched quartz grain is a combination of beta and gamma radiation from radioisotopes in the sediment combined with a cosmic ray component. Beta dose rates were measured using a Risø GM-25-5 low-level beta counting system^16^, using stainless steel and Volkagem loess standards^17^. Gamma dose rates were measured in the field using an EG&G Ortec digiDart-LF gamma-spectrometer, with the data being analysed using the threshold technique^18,19^. Dose rates were corrected for: (i) attenuation due to grain size; (ii) loss of beta dosed material due to HF etching^20^ and (iii) moisture content ^21^. A mean burial water content of 5±2 % was assumed. The 2σ uncertainty on water content encompasses very dry conditions (1%) and saturation for 20% of the burial period (8%), representing the full range of reasonable mean water content scenarios for these samples. Cosmic dose rate was calculated using site location (latitude, longitude and altitude) and present-day burial depth ^22^, assuming an overburden density of 1.85 g/cm^3^. Dose rate data are presented in Supplementary Table 3.

Supplementary Table 3. Sample depth, water content and dose rate for sample T1B-OSL5.

|  | Depth  (m) | Moisture  (%) | Dose rate (Gy/ka) | | | Total (Gy/ka) |
| --- | --- | --- | --- | --- | --- | --- |
|  |  |  | Beta | Gamma | Cosmic |  |
| T1B-OSL5 | 0.95±0.1 | 5±2 | 0.36±0.02 | 0.32±0.02 | 0.20±0.02 | 0.88±0.04 |

The three populations of grains for SAU2 T1B layer 7 are provided in Supplementary Table 4: Age 1: 1.9±0.5 ka (7% of measured grains), Age 2: 10.3±1.1 ka (29% of measured grains), Age 3: 55.0±4.8 ka (64% of measured grains). The small component of younger material may be explained by the known termite bioturbation in the sequence. This is the deepest layer to include artefacts, but only has a low density so it likely represents the contact between the sterile sands and the archaeological sequence above. The degrading sandstone clasts in this layer, including large horizontally bedded pieces (Supplementary Figures 5 and 6), suggests it may have been a stable surface for some time. We infer that the 10.3 ka age represents the start of the archaeological sequence in T1B on the top of this stable surface.

Supplementary Table 4. Ages for sample T1b-OSL5.

| Grain population | Proportion (%) | De (Gy) | Age (ka) |
| --- | --- | --- | --- |
| Age 1 | 7 | 1.7±0.4 | 1.9±0.5 |
| Age 2 | 29 | 9.1±0.9 | 10.3±1.1 |
| Age 3 | 64 | 48.7±3.4 | 55.0±4.8 |

**Supplementary Note 3: Radiocarbon age estimates**

Radiocarbon samples were analysed at the Centre for Applied Isotope Studies (CAIS) at the University of Georgia. Charcoal samples were treated with 5% HCl at 80 °C for one hour, then they were washed with deionized water on a fibreglass filter and rinsed with diluted NaOH to remove potential contamination by humic acids. Samples were then treated with diluted HCl again, washed with deionized water and dried at 60 °C.

Bone samples were cleaned and washed, using an ultrasonic bath. After cleaning, the dried bone was gently crushed to small fragments. The crushed bone was treated with diluted 1N acetic acid to remove surface absorbed and secondary carbonates. Periodic evacuation ensured that evolved carbon dioxide was removed from the interior of the sample fragments, and that fresh acid was allowed to reach even the interior micro-surfaces. The chemically cleaned sample was then reacted under vacuum with 100% phosphoric acid to dissolve the bone mineral and release carbon dioxide from bioapatite. The resulting carbon dioxide was cryogenically purified from the other reaction products and catalytically converted to graphite using the method of Vogel et al.^23^. Graphite 14C/13C ratios were measured using the CAIS 0.5 MeV accelerator mass spectrometer. The sample ratios were compared to the ratio measured from the Oxalic Acid I (NBS SRM 4990). The sample 13C/12C ratios and 15N/14N were measured separately using EA-MS system and expressed as d13C with respect to PDB and d15N with respect to air with an error of less than 0.1‰.

The resulting carbon dioxide was cryogenically purified from the other reaction products and catalytically converted to graphite using the method of Vogel et al.^23^. Graphite 14C/13C ratios were measured using the CAIS 0.5 MeV accelerator mass spectrometer. The sample ratios were compared to the ratio measured from the Oxalic Acid I (NBS SRM 4990). The sample 13C/12C ratios were measured separately using a stable isotope ratio mass spectrometer and expressed as δ13C with respect to PDB, with an error of less than 0.1‰ (Supplementary Table 5).

*Supplementary Table 5. Radiocarbon age estimates for Sahout.* *Context numbers, and in some cases spits, from original excavation are listed to show stratigraphic relationship within each layer. *Denotes radiocarbon ages obtained during original test excavations .*

| **Locality** | **Trench** | **Layer** | **Context** | **Material** | **Lab code (UGAMS-)** | δ13C,‰ | **C14 age** | **Error** | **pMC** | **Calibrated age BP** |
| --- | --- | --- | --- | --- | --- | --- | --- | --- | --- | --- |
| SAU2 | T1A | 4 | Test trench | Bone | 60762* | -7.04 | 7880 | 30 | 37.5 ±0.12 | 8,972-8,590 |
| SAU2 | T1B | 2 | 5 | Charcoal | 65282 | -25.33 | 1640 | 50 | 81.58±0.24 | 1583-1412 |
| SAU2 | T1B | 5 | 14 | Charcoal | 65284 | -10.23 | 8230 | 30 | 35.88 ±0.13 | 9,398-9,028 |
| SAU2 | T1B | 6 | 16 | Charcoal | 65285 | -25.43 | 270 | 20 | 96.68 ±0.28 | 428-156 |
| SAU2 | T1B | 6 | 17 | Bone | 72767 | -10.34 | 8070 | 30 | 36.6 ±0.15 | 9,090-8,779 |
| SAU2 | T1B | 6 | 18 | Bone | 65290 | -13.93 | 8060 | 40 | 36.68 ±0.17 | 9,120-8,772 |
| SAU2 | T2A | 4 | Test trench | Bone | 60763* | -0.21 | 11530 | 30 | 23.81 ±0.09 | 13,465-13,319 |
| SAU2 | T2B | 2 | 3, spit 1 | Tooth | 69966 | 0.15 | 5650 | 30 | 49.46 ±0.18 | 6,495-6,317 |
| SAU2 | T2B | 2 | 3, Spit 2 | Bone | 65287 | -10.49 | 7880 | 30 | 37.5 ±0.15 | 8,972-8,590 |
| SAU2 | T2B | 4 | 9 | Bone | 65288 | -6.78 | 11620 | 35 | 23.55 ±0.11 | 13,556-13,363 |
| SAU2 | T2B | 5 | 15, Spit 1 | Bone | 72766 | -2.57 | 940 | 40 | 88.90±0.41 | 925-744 |
| SAU2 | T2B | 5 | 15, Spit 2 | Bone | 65289 | -10.47 | 7830 | 30 | 37.71 ±0.14 | 8,718-8,540 |
| SAU4_6 | N/A | 2 | N/A | Charcoal | 65286 | -14.84 | 5360 | 30 | 51.32 ±0.17 | 6277-6003 |

Radiocarbon ages were calibrated using the program OxCal v.4.4.4^24^ and the IntCal2020^25^ calibration curve (Supplementary Figure 10).


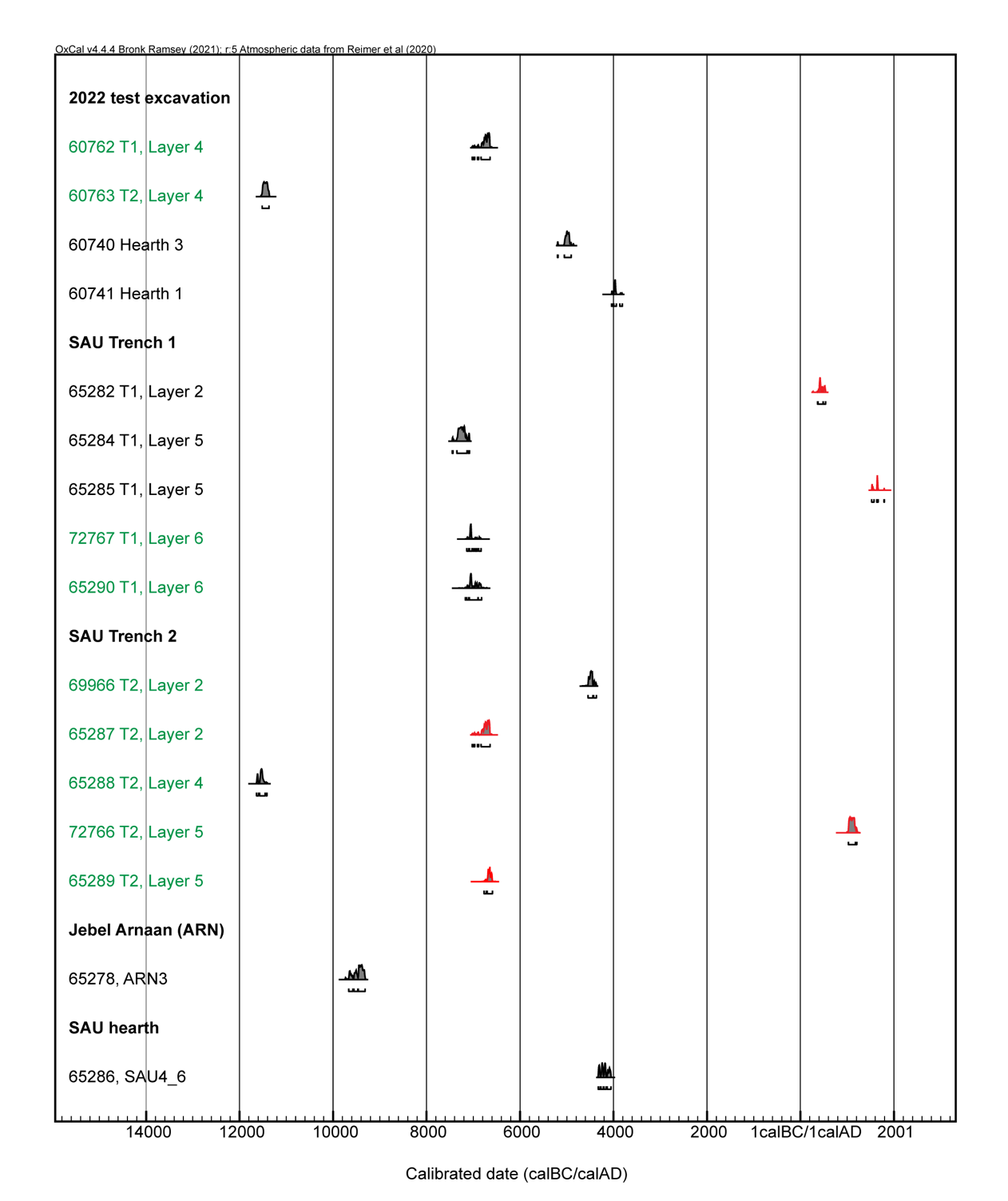


*Supplementary Figure 10. Calibrated radiocarbon dates from Sahout and Jebel Arnaan. Bioapatite ages in green font, charcoal ages in black font; intrusive ages outlined in red (3 of 12 samples from the excavations at SAU2 T1 and T2).*

Bioturbation, likely from termites (Supplementary Figure 4), is evident in both trenches at Sahout, with 3 of 12 radiocarbon samples providing historic ages, and two providing ages too young for their stratigraphic position (Fig. 3). This is the case for microcharcoal as well as bone fragments. However, two-thirds of samples provided ages consistent with their stratigraphic position, and consistent with diagnostic lithic artefacts.

The radiocarbon ages shown in Supplementary Figure 10 suggest there are three discontinuous phases of human occupation from 13.5 to 8.6 ka at Sahout and Jebel Arnaan. However, when we consider the OSL dates, the occupation of this region looks more continuous with ages of 12.2±1.4 and 12.8±1.1 ka from the Jebel Arnaan trench 1 excavation, 12.0±1.8 ka from the Jebel Misma locality 8 excavation^26^, and the 10.3±1.1 ka age described above for SAU2 T1B (Fig. 8).

The excavation of a stone-lined hearth at SAU4_6 revealed it was in use 6.1 ka, in the later Neolithic of Sahout, consistent with stone-lined hearths within the Nefud^27^.

**Supplementary Note 4: Faunal remains**

A total of 98 and 105 bones were recovered from Trenches 1 and 2 respectively (NSP=203) (Supplementary Table 6). Nine tooth and enamel fragments were recovered, including two belonging to *Bos* sp. (aurochs/cattle), two more likely to *Ovis/Capra* sp. (sheep/goat), three other medium-sized ungulate tooth fragments, and a single *Meriones* sp. (gerbil) molar (Supplementary Table 7; Supplementary Figure 11). Other ungulate remains include a gazelle mandible, two tibia fragments, one from a gazelle and another from a larger ungulate, and three metapodial fragments, including two unfused (juvenile) gazelle distal epiphyses. Additional microfauna remains include a gerbil maxilla, a bird proximal phalanx, and a varanid caudal vertebra. Both the *Bos* specimens are from the upper occupation phase 3.

*Supplementary Table 6.* *Skeletal part representation by phase.*

| **Skeletal element** | **Phase 1** | **Phase 2** | **Phase 3** | **Total** |
| --- | --- | --- | --- | --- |
| Mandible | 1 | 1 |  | 2 |
| Tooth fragment |  | 2 | 6 | 8 |
| Metapodial | 2 | 1 |  | 3 |
| Midshaft | 15 | 26 | 26 | 67 |
| Tibia | 1 |  | 1 | 2 |
| Proximal phalanx |  |  | 1 | 1 |
| Vertebra | 1 |  |  | 1 |
| Rib | 1 | 2 | 1 | 4 |
| Indeterminate | 43 | 53 | 19 | 115 |
| **Total** | **64** | **85** | **54** | **203** |

*Supplementary Table 7. Taxonomic representation by phase.*

| **Phase 1** | **Phase 2** | **Phase 3** |
| --- | --- | --- |
| *Gazella* sp. | *Gazella sp.* | *Bos* sp. |
| *Varanus* sp. | *Meriones* sp. | cf. *Ovis* / C*apra* sp. |
|  |  | *Gazella* sp. |
|  |  | Aves |


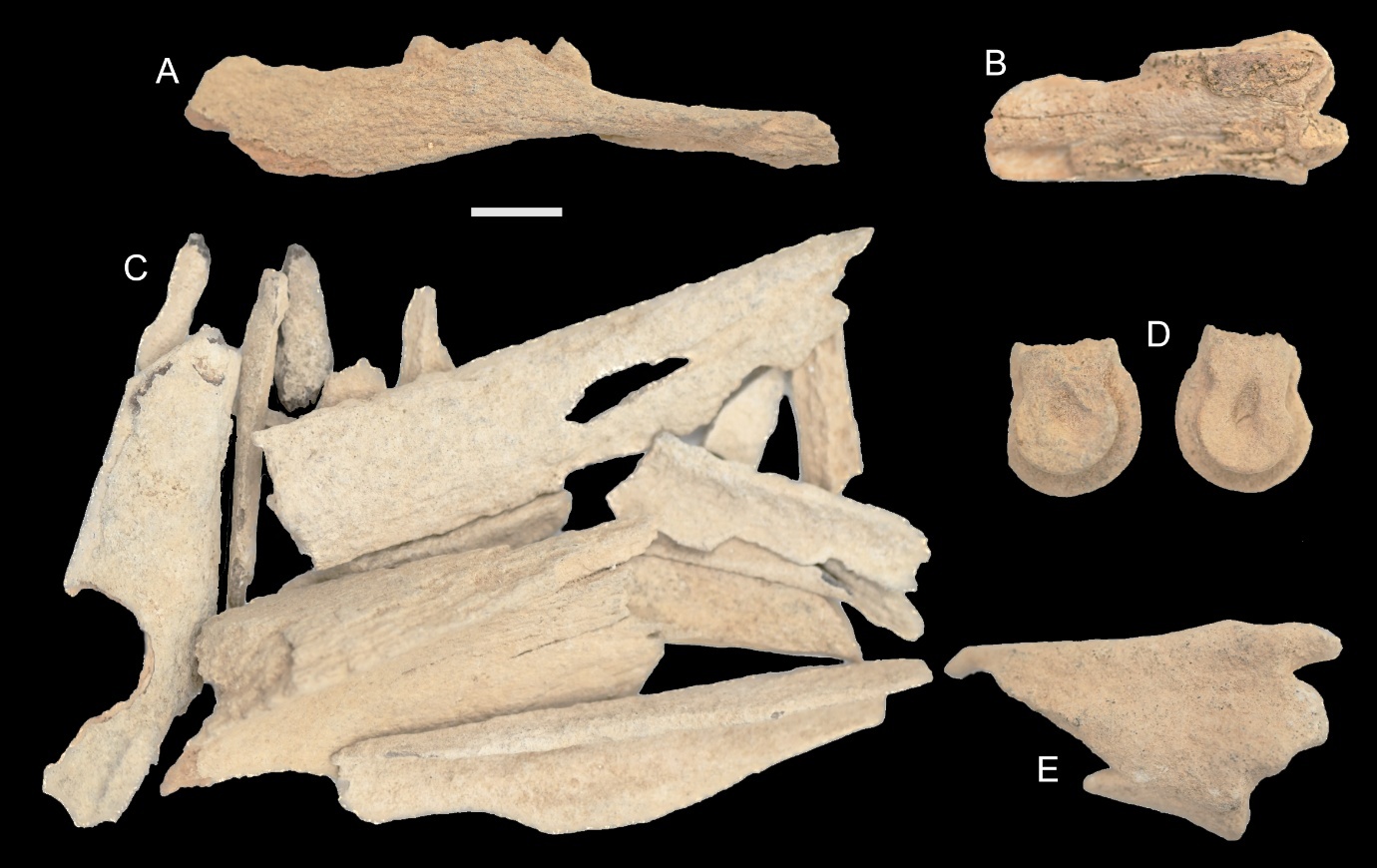


***Supplementary Figure 11****. Bones from the SAU excavations. A: gazelle mandible fragment; B:* Bos *sp. tooth enamel fragment; C: general condition of bones recovered from T2B; D: two unfused distal gazelle metapodials; E: gazelle right distal tibia. Scale bar is 1 cm.*

Overall, the assemblage is poorly preserved and fragmented, with a mean specimen length and width of 23 and 7 mm, respectively. Only 88 (43%) are identifiable to a specific skeletal element, roughly two-thirds of which are small midshaft fragments. Cortical readability is overall very poor since over half (53% of NSP) of the assemblage is covered in cemented sands, making the analysis of any bone surface modifications difficult. Despite this, it is notable that 16% (n=33) are charred, including a small number (n=4) of calcined bone indicating heating at high temperatures^28^. In addition, 20 midshafts exhibit green fracture patterns indicating breakage while the bone was fresh, likely related to marrow extraction. A small number of bones (n=10) exhibit some minor manganese staining, occurring as small dots on the cortical surface.

Breaking the assemblage down by phase reveals a similar picture throughout sequence (Supplementary Table 8). All phases are dominated by small indeterminate bone fragments, followed by midshaft fragments, and just a handful of axial and appendicular remains. Likewise, the preservation and condition of the remains is similar throughout the phases, with most specimens across all three phases being covered by cemented sands. Burning/charring is similar across the phases. The main noticeable difference, keeping in mind the extremely small sample sizes, is the presence of cattle and the likely presence of sheep/goat in phase 3.

Taken together, the assemblage appears to represent a butchered assemblage of small (e.g., gazelle) and large (e.g., aurochs/cattle) ungulates, as well as potentially smaller animals like birds and varanids, though the latter could also represent natural deaths at the site.

*Supplementary Table 8.* *Taphonomic attributes by phase (%NSP).*

|  | **Phase 1** | **Phase 2** | **Phase 3** |
| --- | --- | --- | --- |
| Adhering sediment | 33 (63%) | 57 (67%) | 33 (69%) |
| Manganese staining | 3 (6%) | 1 (1%) | 7 (14%) |
| Corrosion | 4 (8%) | - | 3 (6%) |
| Exfoliation | 1 (2%) | 1 (1%) | - |
| Rounding | - | 1 (1%) | 1 (2%) |
| Charred | 11 (21%) | 16 (19%) | 6 (8%) |

**Supplementary Note 5: Grinding stones**

In T1B, seventeen grinding stones were recovered from layer 4 (phase 2), with one from layer 6 (phase 2) and one from layer 3 (phase 3). In T2B, one grinding stone was from layer 4 (phase 1), three each were from layers 3 (phase 2) and 2 (phase 3), and one was from layer 1 (phase 3).

Grindstones (bottom stones) were mostly made of sandstone except for two pieces of ferruginous sandstone, while the mullers (top stones) were mostly made of the latter tougher material, except for one piece of quartzite in T1B layer 4 (phase 2). Two of the mullers were made on large flakes in T1B layer 4. Five grindstones, two from T1B layer 4 and one each from T2B layers 3 (phase 2) and 4 (phase 1), had their edges prepared through unifacial flaking with the rest unmodified natural slabs. The few sandstone flakes are concentrated in T1B layers 3 (phase 3) and 4 (phase 2) in accordance with this unifacial flaking preparation of grindstone edges. Grindstones were on average 28.21±15.18 mm thick, with the longest measuring 210 mm. Grinding surfaces on bottom stones were typically flat, with two concave pieces that were among the most well-used. The mullers all only had one grinding surface, which was convex, except for the piece from T1B layer 6 (phase 2) which had 4 grinding surfaces with the main surface being flat. A large flake was removed from the apex of the pyramidal form of this muller, perhaps to aid the grip. One of the bottom stones from T1B layer 4 (phase 2) is a flake removing part of the edge, perhaps because of a small lump protruding on the underside that may have made the grindstone unstable (Supplementary Figure 12). This piece has red ochre residue on it (Supplementary Figure 12) with red ochre found throughout the SAU2 sequences (e.g. Supplementary Figure 13) indicating that red paint production was one of the functions of these grindstones. However, the lack of such residue on other grindstones suggests ochre grinding was not the primary purpose of these tools, with plant and bone processing likely alternatives as indicated by the sickle blade from T1B layer 4 (phase 2) and the green fractured bone respectively. Ochre, plant, and bone processing are all attested on grinding stones at the later Neolithic site of Jebel Oraf 2 in the Nefud^29^. In addition to the grinding stones from the excavation, a series of grinding slicks on the sandstone bedrock was observed at SAU4 (Fig.1). Of the grindstones from the Sahout excavations, 74% were in the rubbly layers, with a glossed sickle blade from this phase suggesting cereal grinding was one of their functions. In the Tayma palaeolake record, 125 km to west of Sahout, grass pollen reaches a peak at 8.6 ka^30^. Grindstones are also concentrated in the clast rich layer at Jebel Misma 7 (6 out of 8 pieces)^26^.

***
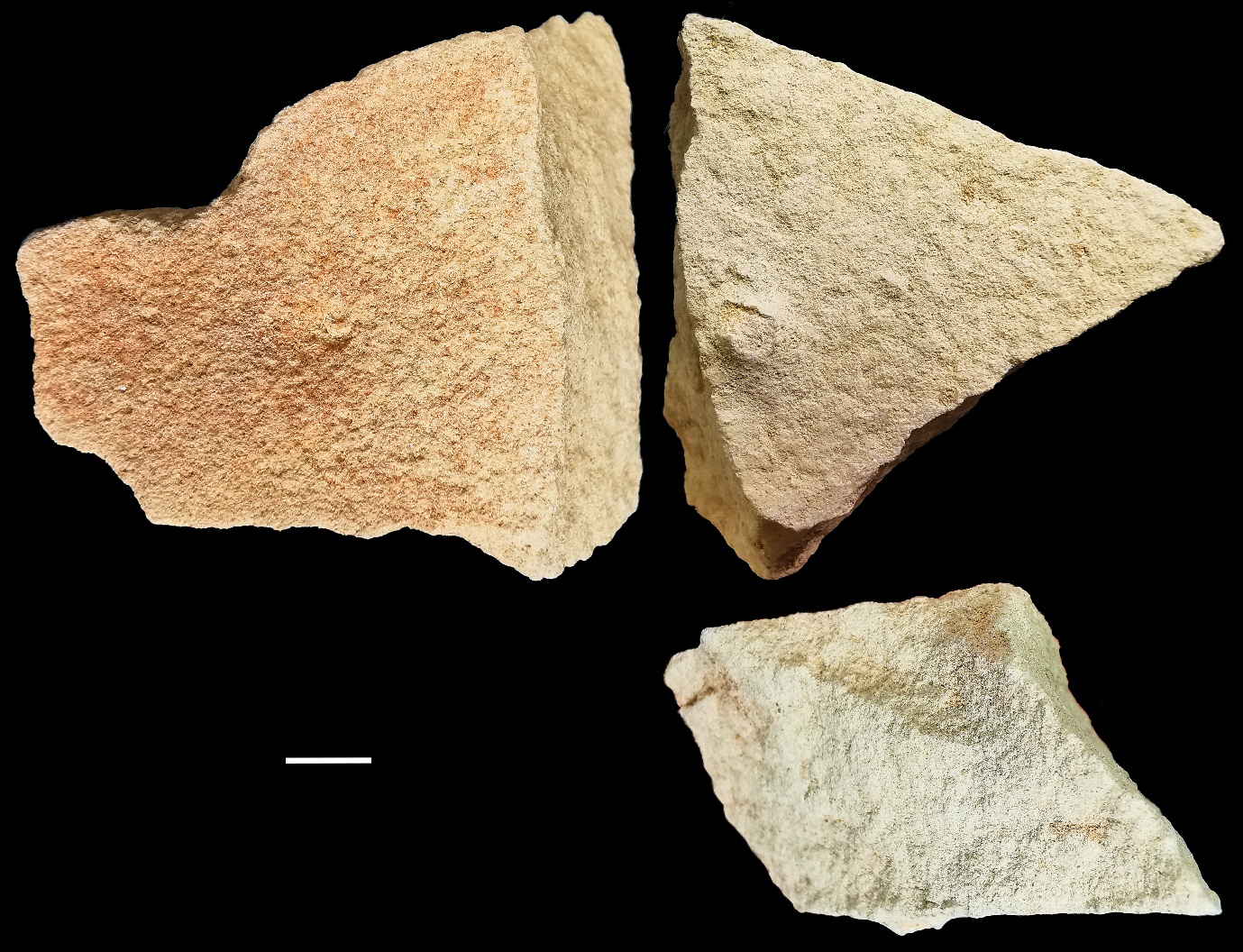
***

*Supplementary Figure 12. Flake from T1B layer 4 (phase 2) removing the edge of a sandstone bottom grindstone with ochre residue on its ground surface (left). Note the lump on the bottom lower surface of the grindstone (bottom right) that may have prevented the slab from lying flat. Scale is 1 cm.*

***
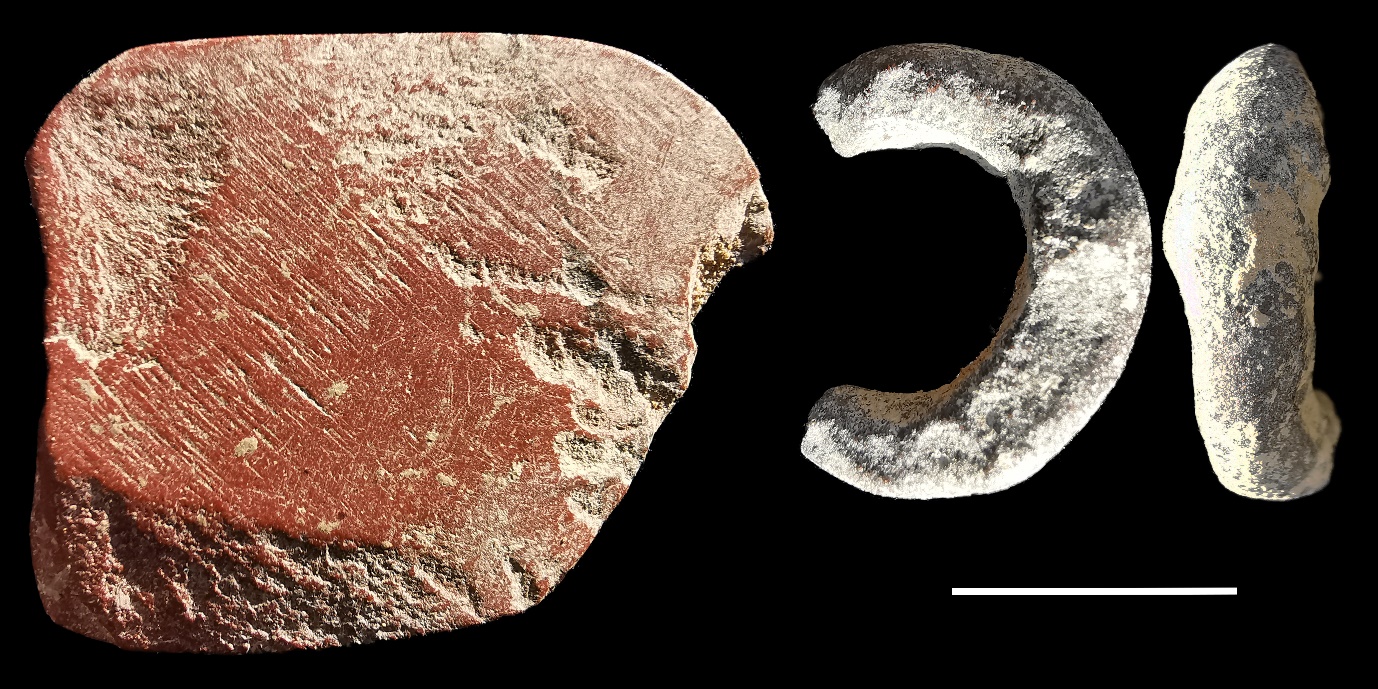
***

*Supplementary Figure 13. Red ochre crayon and stone bead from SAU2 T2B layer 4 (phase 1). Scale is 1 cm.*

***Supplementary Note 6: SAU2 Lithics***

A total of 1896 lithics were recovered from the SAU2 excavations (lithics from emptying the previous trenches were not included in these analyses). The ten lithics from T1B layer 7 were combined with layer 6 for the analyses below as these are presumed to belong to the same reoccupation phase of the site (Supplementary Note 2).

Nine different rock types were used for knapping (Supplementary Table 9). The peach-coloured silcrete locally available at SAU4, a red-yellow chert and a dark blueish variety whose sources are unknown, milky and crystal quartzes which are available at Jebel Misma, whiteish chalcedony whose source is unknown, purple ferruginous sandstone (orthoquartzite – hereafter referred to as ferruginous) which is widely available in the region including at Jebel Misma, a striped greenish grey exotic obsidian (referred to as fine-grained rhyolite in the test excavations^31^), and khaki sandstone which is locally available at Sahout. A chi-square test of the proportions of chert and silcrete by layer in T2B showed the switch from the former to the latter between layers 4 and 3 (phases 1 and 2) was highly significant (χ=40.76, p<0.00001). Similarly, a chi-square test of materials (combining the two cherts and excluding sandstone and obsidian) between T2B layers 4 and 5 (phase 1) versus T1B layers 5 and 6 (phase 2) showed the switch away from chert, crystal quartz, chalcedony, and ferruginous, to silcrete was highly significant (χ=162.51, p<0.00001).

A t-test comparing Shannon’s diversity index on all materials (excluding sandstone) between phases 1 (H=1.755) and the earlier layers of phase 2 (T1B layers 5 and 6) (0.944) showed significantly more material diversity in phase 1 (t=11.457, df=634, p<0.00001). There was no difference between the earlier part of phase 2 and the rocky layers (layer 4 in T1B and layer 3 in T2B, H=0.937) (t=0.092, df=1112, p=0.926), but there was a return to higher diversity in phase 3 in comparison to the rocky layers (H=1.297) (t=5.31, df=1042, p<0.00001). The same range of materials were used at Jebel Arnaan (ARN3), which sits intermediate in time between phases 1 and 2 at Sahout (Fig. 2)^26^. Shannon’s diversity index for ARN3 was 1.726, comparable to phase 1, with a t-test also showing it to be significantly more diverse than phase 2 at SAU2 (H=0.946) (t=16.735, df=1691, p<0.00001). To test if this pattern was being driven by the preference for silcrete in the later occupation phases and the proximity of the source to SAU2, we compared diversity at the more distal site of Jebel Misma (JMI8), where there is also a switch to silcrete in the upper part of the sequence^26^. (Note the two quartzes are lumped together for this analysis as the distinction between the two is less clear at JMI8.) The results showed significantly lower diversity in layers 1-3 of JMI8 (H=1.054) than either ARN3 (t=8.095, df=466, p<0.00001) or SAU2 T2B phase 1 (t=8.581, df=495, p<0.00001). This indicates the pattern of reduced diversity in the later occupation phases at SAU2 is partly independent of the switch to silcrete.

Twenty cores were recovered in the SAU2 excavations (1% of the total assemblage). The largest core was an assayed clast of silcrete weighing 210 g from T2B layer 4. Bifacial (discoidal and hierarchical disc) cores were the most common, with individual examples in T2B layers 2 and 4 and T1B layer 2, as well as four pieces in T1B layer 4. Five unifacial cores were found in T1B layer 4, with one in the equivalent rocky layer (3) in T2B (phase 2). T1B layer 4 also produced two bipolar cores and one multi-facial core, with a single multi-facial core from T1B layer 3. There were three laminar blade cores in the SAU sequences, a chert bidirectional disc from T2B layer 1 (phase 3), a chert single platform core-on-flake from T1B layer 4 (phase 2), and a silcrete naviform core from T1B layer 5 (phase 2) (Supplementary Figure 14). Silcrete naviform cores were also found on the surface at the silcrete workshop (Supplementary Figure 14). Within the systematic collection square at the silcrete workshop, there were four multi-facial cores; one unidirectional blade core, and three naviform cores (Supplementary Figure 15). Cresting blades were present at the silcrete workshop, indicating the naviform blade reduction sequence was initiated there with these technical knapping by-products that create two parallel elongate ridges to guide future blade removals (Supplementary Figure 16). Cresting blades and naviform cores were also used at Dumat al-Jandal on the other side of the Nefud^32^.

Blade and bladelet production on chert and obsidian often involved grinding platforms, with 12 examples of ground platform blades spanning all occupation phases: T1B layer 4 and T2B layers 1-4 (Supplementary Figure 17). Low platform angles on the two thin chert blade cores from SAU2 are matched by low angled lipped platforms on many of the blades (Supplementary Figure 18); this probably reflect a soft-hammer blade production strategy as has been documented at Al-Rabyah^33^. Larger blades on dark chert and occasionally obsidian, often with ground platforms, were also documented at Jebel Arnaan^26^.

Lithic numbers peaked in phases 2, accounting for 64% of the whole assemblage. A total of 45 retouched artefacts were recovered from the SAU2 excavations. A chi-square test showed they occurred disproportionately in T2B layers 4-5 (phase 1) (χ=15.305, p=0.0015) (Supplementary Table 10).

The dominant type of retouch was Helwan (Supplementary Table 10), a distinctive mode of invasive bifacial backing through pressure flaking, characteristic of the Natufian^34^. There were 12 examples of Helwan retouch; 11 Helwan bladelets and 1 Helwan lunate (Fig. 3, Supplementary Figure 19). Five of the Helwan bladelets were from T2B layer 4 (phase 1), with the Helwan lunate from layer 3 (phase 2), and a further Helwan bladelet from layer 2 (phase 3). In T1B there were two Helwan bladelets each in layers 4 and 5 (phase 2) and one in layer 2 (phase 3). Five other backed bladelets were found, but in each case the backing was on the ventral surface with a low angle between it and the dorsal so it could be described as pseudo-Helwan (Supplementary Figure 20), with such ventrally backed pieces common in Natufian assemblages^34^. These pieces were all from T1B, with three in layer 6 (phase 2) and one each in layer 4 (phase 2) and layer 1 (phase 3).

Six bifacial points were found in the excavations. T1B layers 4 and 5 (Phase 2) included PPN arrowhead types. In T1B layer 5 (phase 2) there was the broken proximal of an obsidian El Khiam point, alongside a complete silcrete arrowhead (cf. Byblos), and a complete silcrete Abu Salem point (Fig. 3, Supplementary Figure 21). Two silcrete Abu Salem points were also found in the systematic surface collection at the SAU4 silcrete source (Supplementary Figure 22). The Abu Salem point from Jebel Oraf in the Nefud was also made of silcrete^35^. In SAU2 T1B layer 4 (phase 3) there were two broken arrowhead tangs, one a unifacial piece on chert and the other a bifacial piece on silcrete, both likely made on blades and their size indicating they may be from Byblos points (Supplementary Figure 21). Byblos points are known from ORF207 in the Nefud Desert^35^. In T2B a complete Ha-Parsa later Neolithic point was found in layer 2 (phase 3) (Supplementary Figure 21). A bifacially retouched flake from T1B layer 4 (phase 2) may be the blank for a point. Tangs and notches on the arrowheads are too fine to have been made using freehand percussion and must have involved pressure flaking.

*Supplementary Table 9. Lithic materials by layer for the SAU2 excavations. Note that layer 6 also includes the 10 artefacts from layer 7.*

| **SAU2 T1B** | **Silcrete** | **Chert** | **Dark chert** | **Quartz** | **Crystal quartz** | **Chalcedony** | **Ferruginous** | **Obsidian** | **Sandstone** | **Total** |
| --- | --- | --- | --- | --- | --- | --- | --- | --- | --- | --- |
| **Layer 1** | 35 (54%) | 14 (22%) | 2 (3%) | 3 (5%) | 5 (8%) | 3 (5%) | 2 (3%) | 0 | 0 | 64 (100%) |
| **Layer 2** | 48 (58%) | 13 (16%) | 0 | 10 (12%) | 2 (2%) | 5 (6%) | 2 (2%) | 2 (2%) | 1 (1%) | 83 (100%) |
| **Layer 3** | 71 (54%) | 25 (19%) | 2 (2%) | 13 (10%) | 5 (4%) | 4 (3%) | 4 (3%) | 1 (<1%) | 7 (5%) | 132 (100%) |
| **Layer 4** | 511 (75%) | 83 (12%) | 9 (1%) | 29 (4%) | 11 (2%) | 10 (2%) | 21 (3%) | 3 (<1%) | 8 (1%) | 685 (100%) |
| **Layer 5** | 283 (75%) | 55 (15%) | 1 (>1%) | 16 (4%) | 4 (1%) | 9 (2%) | 5 (1%) | 3 (1%) | 0 | 376 (100%) |
| **Layer 6** | 82 (66%) | 23 (18%) | 1 (1%) | 7 (5%) | 6 (5%) | 6 (5%) | 0 | 0 | 0 | 125 (100%) |
| **Total** | **1029** | **214** | **15** | **78** | **33** | **37** | **34** | **9** | **16** | **1465** |
| **SAU2 T2B** |  |  |  |  |  |  |  |  |  |  |
| **Layer 1** | 24 (60%) | 10 (25%) | 0 | 2 (5%) | 1 (2.5%) | 2 (5%) | 1 (2.5%) | 0 | 0 | 40 (100%) |
| **Layer 2** | 84 (60%) | 31 (22%) | 0 | 7 (5%) | 1 (1%) | 6 (4%) | 7 (5%) | 2 (1.5%) | 2 (1.5%) | 140 (100%) |
| **Layer 3** | 29 (68%) | 7 (16%) | 1 (2%) | 3 (7%) | 2 (5%) | 0 | 0 | 1 (2%) | 0 | 43 (100%) |
| **Layer 4** | 41 (21%) | 64 (34%) | 26 (14%) | 8 (4%) | 22 (12%) | 19 (10%) | 9 (5%) | 1 (<1%) | 1 (<1%) | 191 (100%) |
| **Layer 5** | 6 (21%) | 13 (43%) | 1 (7%) | 3 (11%) | 0 | 3 (11%) | 1 (3.5%) | 1 (3.5%) | 0 | 28 (100%) |
| **Total** | **184** | **124** | **28** | **23** | **25** | **29** | **18** | **5** | **3** | **442** |

The continued presence of Helwan bladelets and the lunate in phases where they are associated with Neolithic point types is puzzling. They may have moved up the sequence through bioturbation or they may represent genuine local longevity of Helwan retouch.

Notches and scrapers constituted 10 of the retouched pieces, with two of the scrapers being end-scrapers with rounding wear on their distal retouched ends (Supplementary Figure 23). There were four marginally retouched blades from the SAU2 excavations (Supplementary Table 10), with one specimen from T1B layer 4 (phase 3) having sickle gloss on it (Supplementary Figure 24). Three drills accord with the recovery of stone beads from the excavation, perhaps due to on-site manufacture (Supplementary Figure 22). Two burins were found in T1B layer 4 (phase 3) (Supplementary Table 10). Two other miscellaneous chert retouched artefacts were an elongate bifacial piece from T2B layer 4 (phase 1) and a flake where the retouch did not alter the edge angle from T2B layer 1 (phase 3) (Supplementary Table 11).

*Supplementary Table 10. The proportion of retouched artefacts and cores by occupation phase at SAU. Debitage includes blades, flakes, and flaked pieces.*

| Phase | Retouched | Cores | Debitage | Total |
| --- | --- | --- | --- | --- |
| 3: T1B 3-1 & T2B 2-1 | 8 (1.7%) | 2 (0.4%) | 449 | 459 |
| 2 rocky: T1B 4 & T2B 3 | 15 (2.1%) | 15 (2.1%) | 698 | 728 |
| 2 lower: T1B 6-5 | 9 (1.8%) | 1 (0.2%) | 491 | 501 |
| 1: T2B 5-4 | 13 (6.4%) | 2 (1%) | 193 | 204 |
| Total | 45 (2.4%) | 20 (1.1%) | 1851 | 1896 |

*Supplementary Table 11. Retouched artefact types by layer in the SAU2 excavations.*

| Phase | T1B Layer | Helwan | Other Backed | Bifacial point | Notch | Burin | Drill | Scraper | Marginal blade | Misc. | Total |
| --- | --- | --- | --- | --- | --- | --- | --- | --- | --- | --- | --- |
| 3 | 1 |  | 1 |  | 1 |  |  |  | 1 |  | 3 |
| 3 | 2 | 1 |  |  |  |  |  |  |  |  | 1 |
| 3 | 3 |  |  |  |  |  |  |  |  |  | 0 |
| 2B | 4 | 2 | 1 | 3 | 2 | 2 | 1 | 1 | 1 |  | 13 |
| 2A | 5 | 2 |  | 3 |  |  | 1 |  |  |  | 6 |
| 2A | 6 |  | 3 |  |  |  |  |  |  |  | 3 |
|  | T2B layer |  |  |  |  |  |  |  |  |  |  |
| 3 | 1 |  |  |  |  |  |  |  |  | 1 | 1 |
| 3 | 2 | 1 |  | 1 |  |  | 1 |  |  |  | 3 |
| 2B | 3 | 1 |  |  | 1 |  |  |  |  |  | 2 |
| 1 | 4 | 5 |  |  |  |  |  | 2 | 1 | 1 | 9 |
| 1 | 5 |  |  |  | 2 |  |  | 1 | 1 |  | 4 |
|  | Total | 12 | 5 | 7 | 6 | 2 | 3 | 4 | 4 | 2 | 45 |

Aside from the excavations and the systematic surface collection, the other noteworthy artefacts found during fieldwork were a broken chert El Khiam point from the surface lithic scatter (Supplementary Figure 25) and a ferruginous Levallois core on the surface near SAU1 (Supplementary Figure 26).


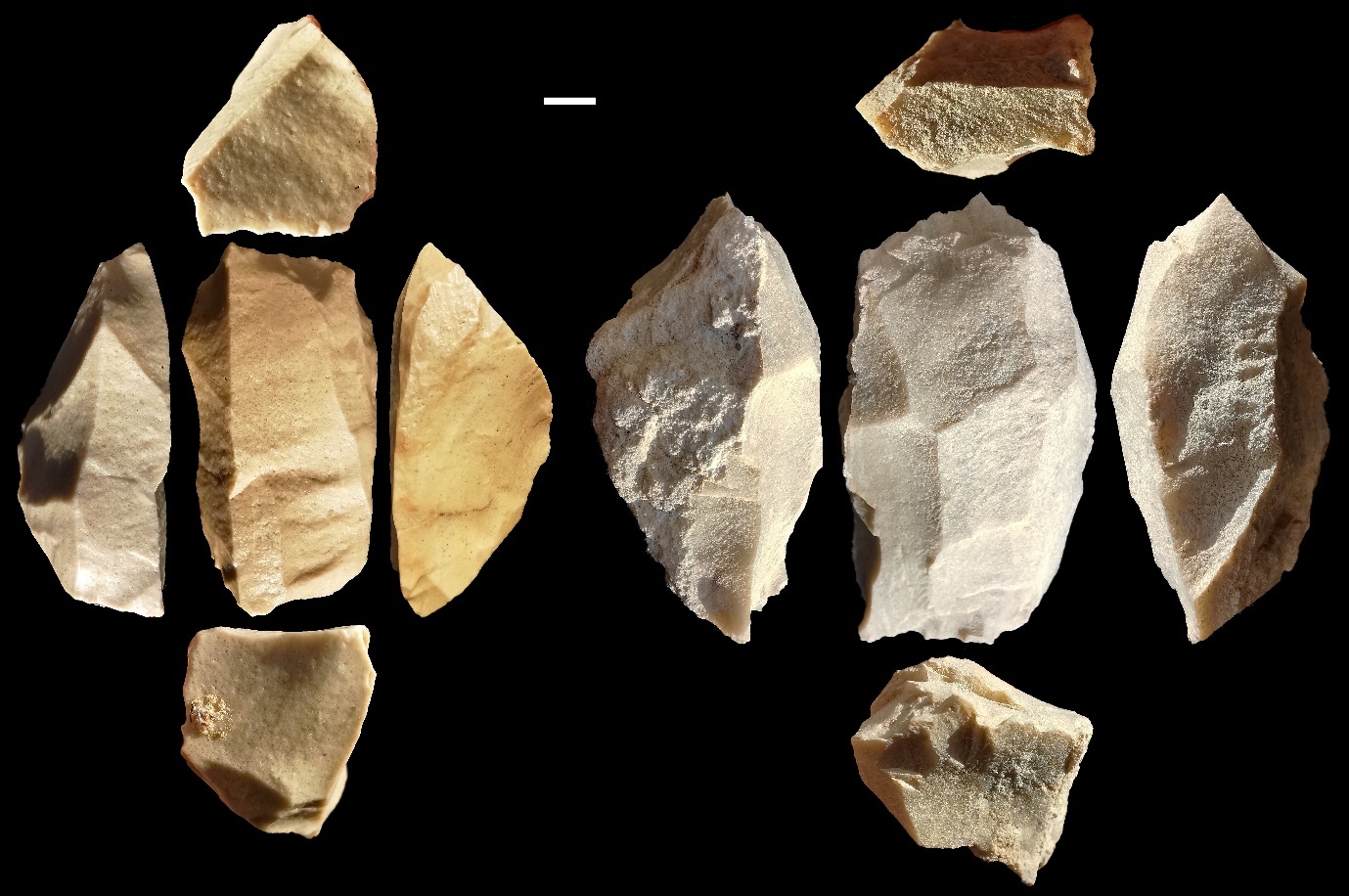


*Supplementary Figure 14. Silcrete naviform cores from Sahout. Left is from the dense surface scatter at the silcrete workshop, right is from the excavation of SAU2 T1B layer 5 (phase 2). Note the keel-shape in profile and the bidirectional blade scars on the main flaking surface. Scale is 1 cm.*

*
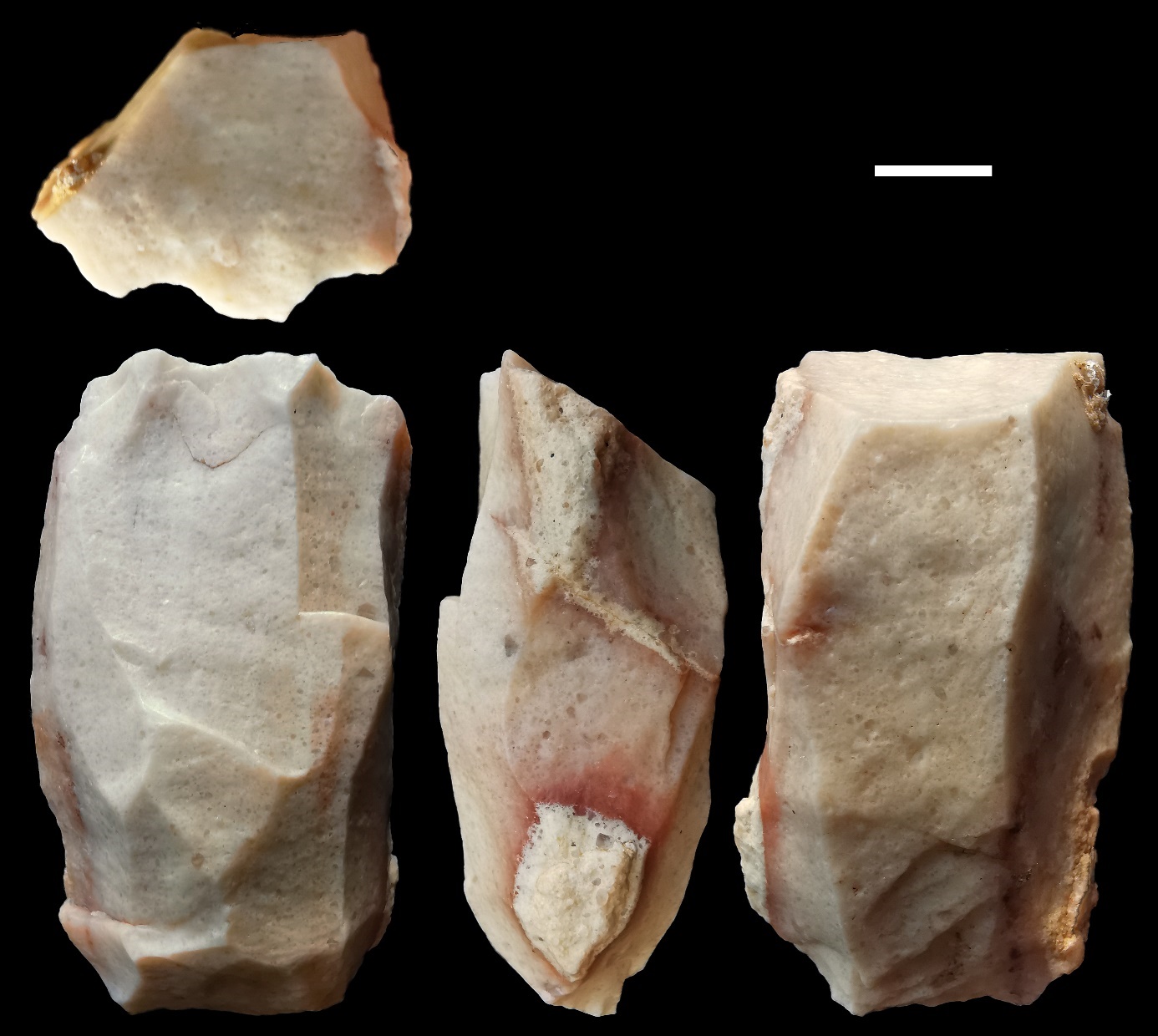
*

*Supplementary Figure 15. Silcrete unidirectional naviform core from the systematic surface collection at the silcrete workshop. Scale is 1 cm.*

*
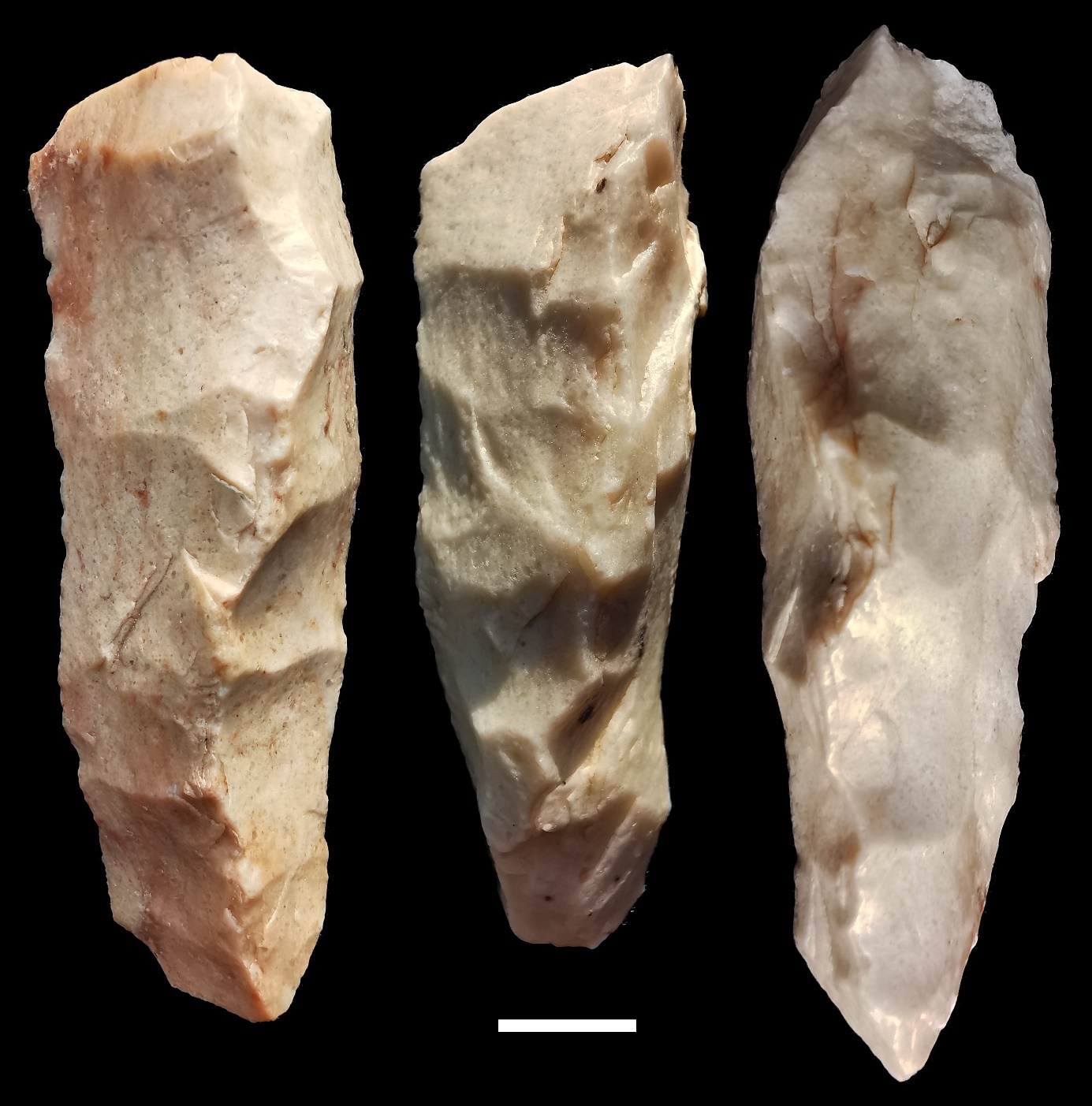
*

*Supplementary Figure 16. Cresting blades from the silcrete workshop at Sahout. The piece on the right is from the systematic surface collection. Scale is 1 cm.*

*
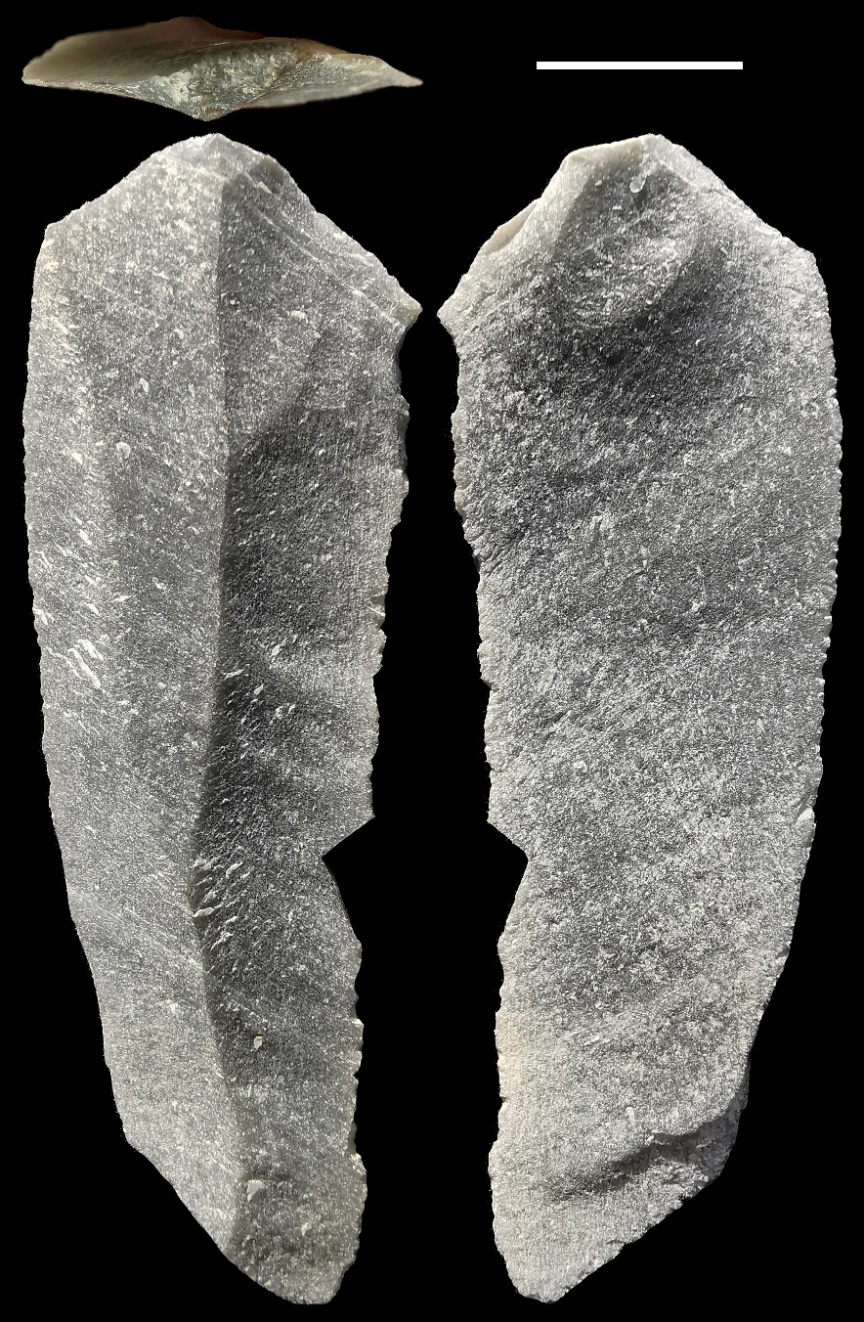
*

*Supplementary Figure 17. Obsidian blade from SAU2 T2B layer 4 (phase 1). Note the grinding on the platform. Scale is 1 cm.*


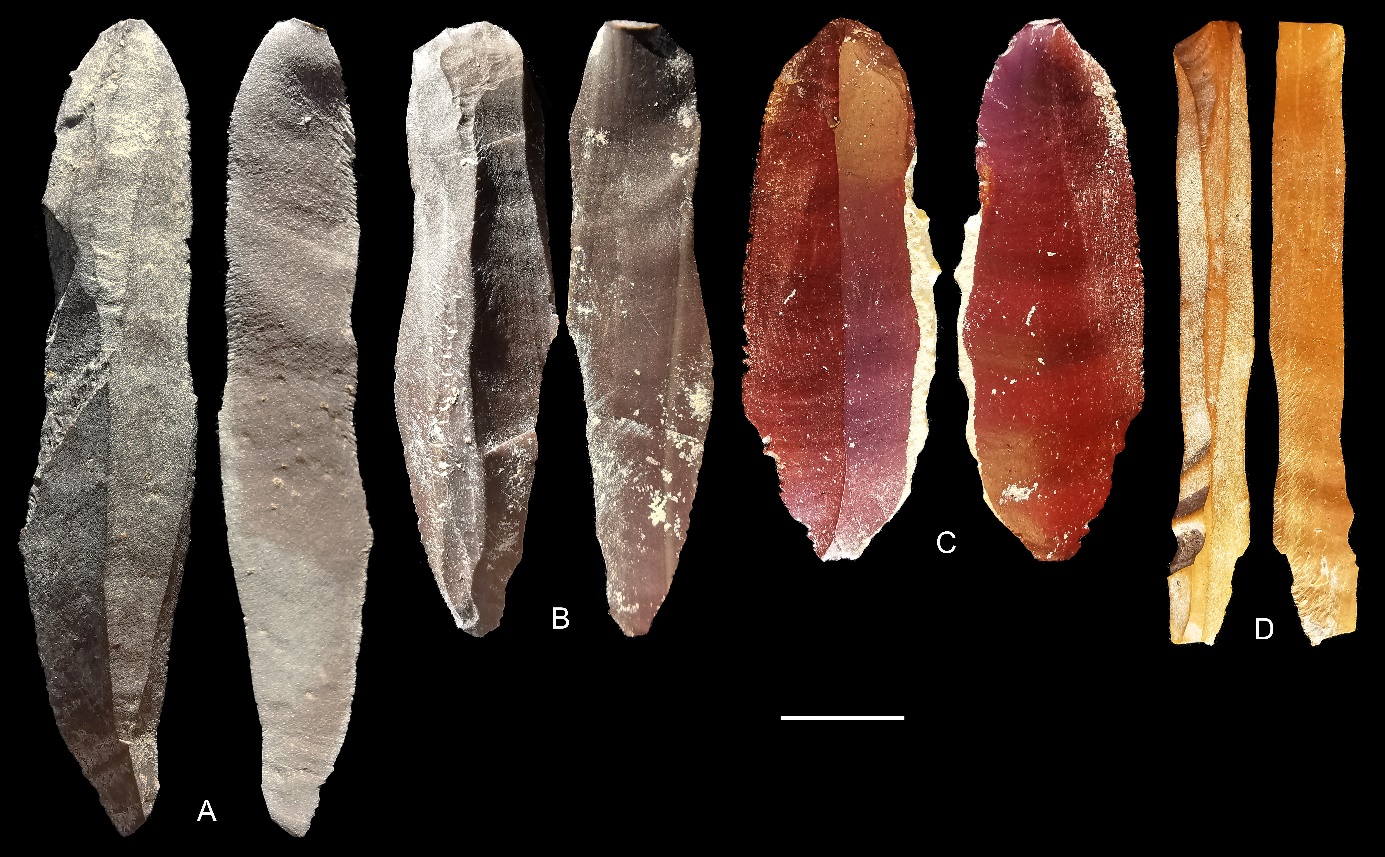


*Supplementary Figure 18. Chert bladelets from SAU2. A & B: from T2B layer 5 (phase 1), C: from T2B layer 4 (phase 1); D: from T2B layer 1 (phase 3). Note the platforms on B and C are low angled enough that they are visible in the ventral view (the platform on D has broken off). C has marginal retouch on the edge opposite the white cortex. Scale is 1 cm.*

*
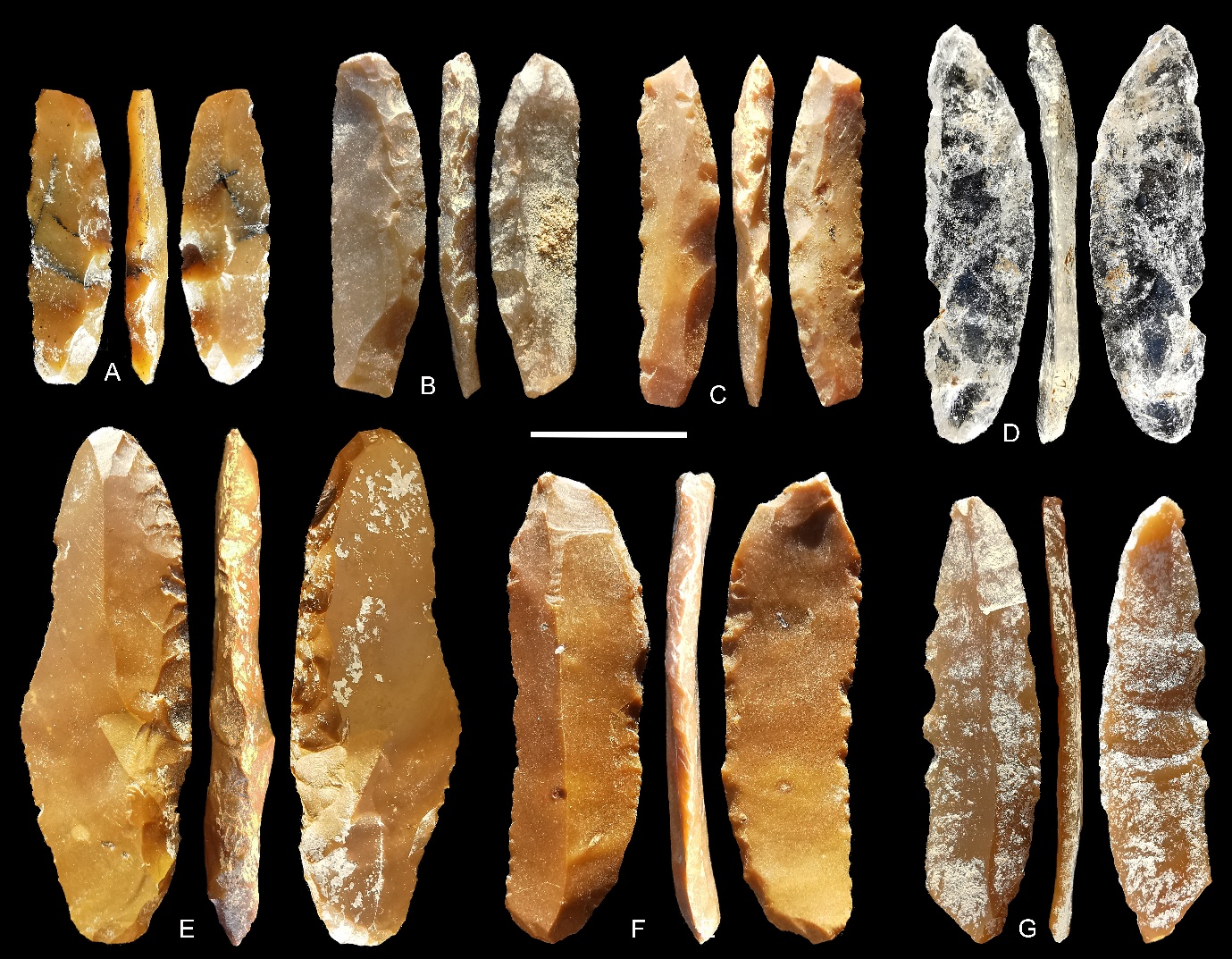
*

*Supplementary Figure 19. Helwan bladelets showing the distinctive invasive bifacial backing from the SAU2 excavations. A and E are chert pieces from T2B layer 4 (phase 1), B is a chert piece from T1B layer 2 (phase 3), C and F are chert pieces from T1B layer 1 (phase 3), D is a crystal quartz piece from T1B layer 5 (phase 2), and G is a chert piece from T1B layer 5 (phase 2). Scale is 1 cm.*

*
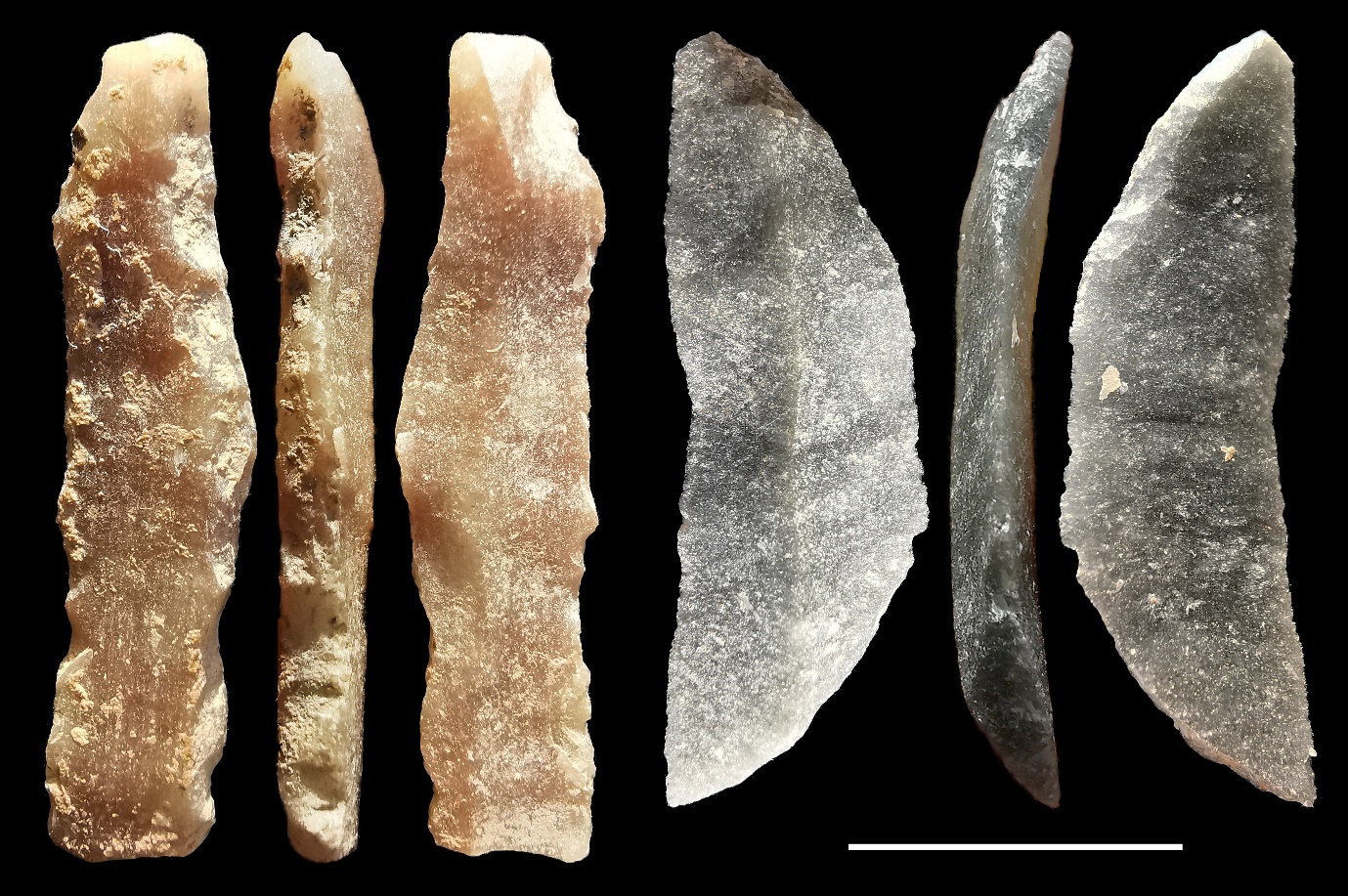
*

*Supplementary Figure 20. Backed artefacts from the SAU2 excavations. Left is a chert piece with pseudo-Helwan retouch (unifacial flaking adjacent to a low angled surface) from T1B layer 6 (phase 2); right is an obsidian Helwan lunate from T2B layer 3 (phase 2) (note the Helwan retouch at the distal end and the standard backing at the proximal). Scale is 1 cm.*

*
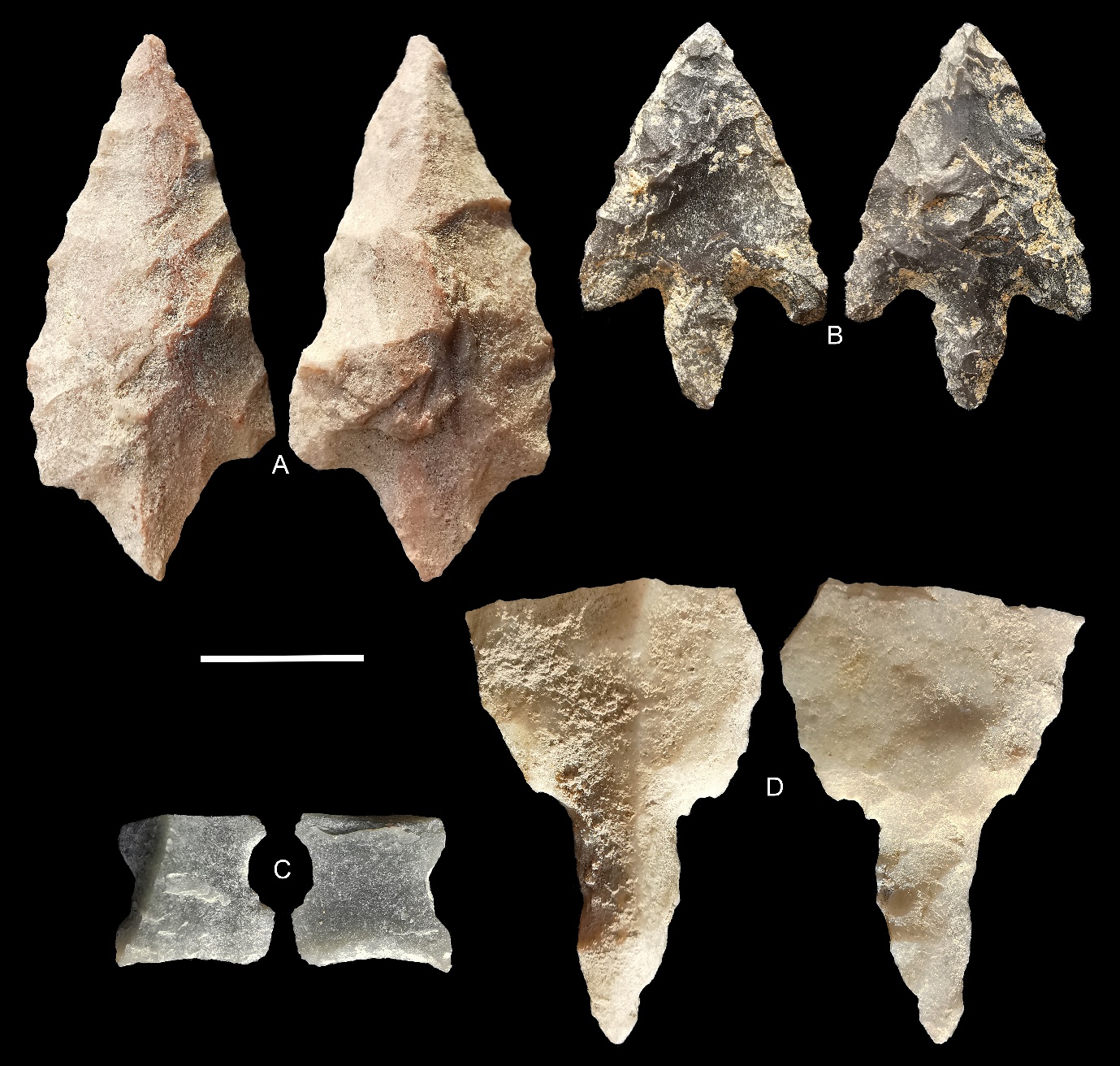
*

*Supplementary Figure 21. Bifacial point types from the SAU2 excavations. A: complete silcrete piece (cf. Byblos) from T1B layer 5 (phase 2); B complete chert Ha-Parsa arrowhead from T2B layer 2 (phase 3); C broken proximal of an obsidian El Khiam arrowhead from T1B layer 5 (phase 2); D broken proximal of a silcrete point (cf. Byblos) from T1B layer 4 (phase 2). Scale is 1 cm.*

*
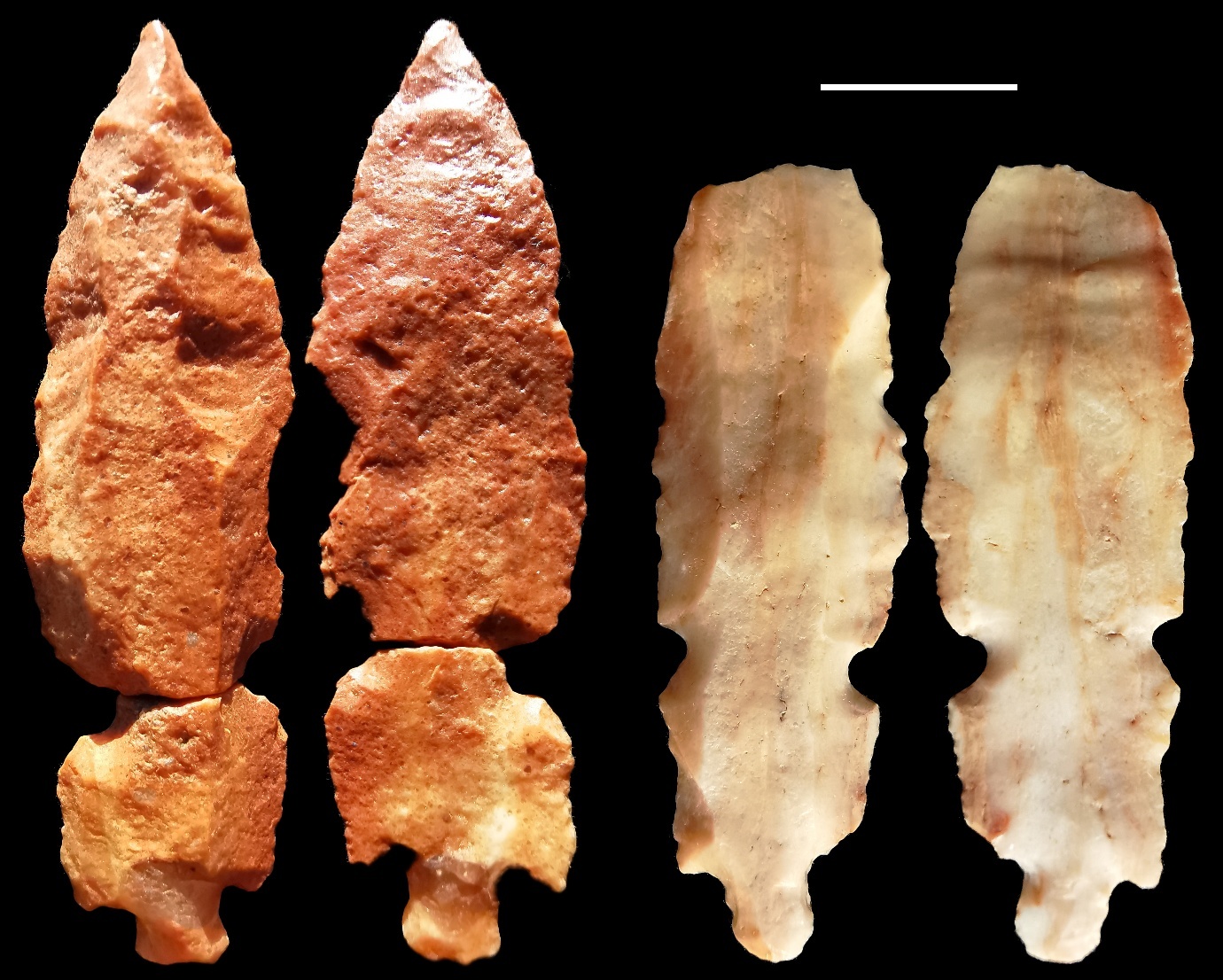
*

*Supplementary Figure 22. Silcrete Abu Salem points from SAU4 surface collection square. The piece on the left has an ancient break between the deep lateral notches which probably occurred during their creation resulting in its abandonment. The piece on the right was abandoned with the tip unfinished. Scale is 1 cm.*

*
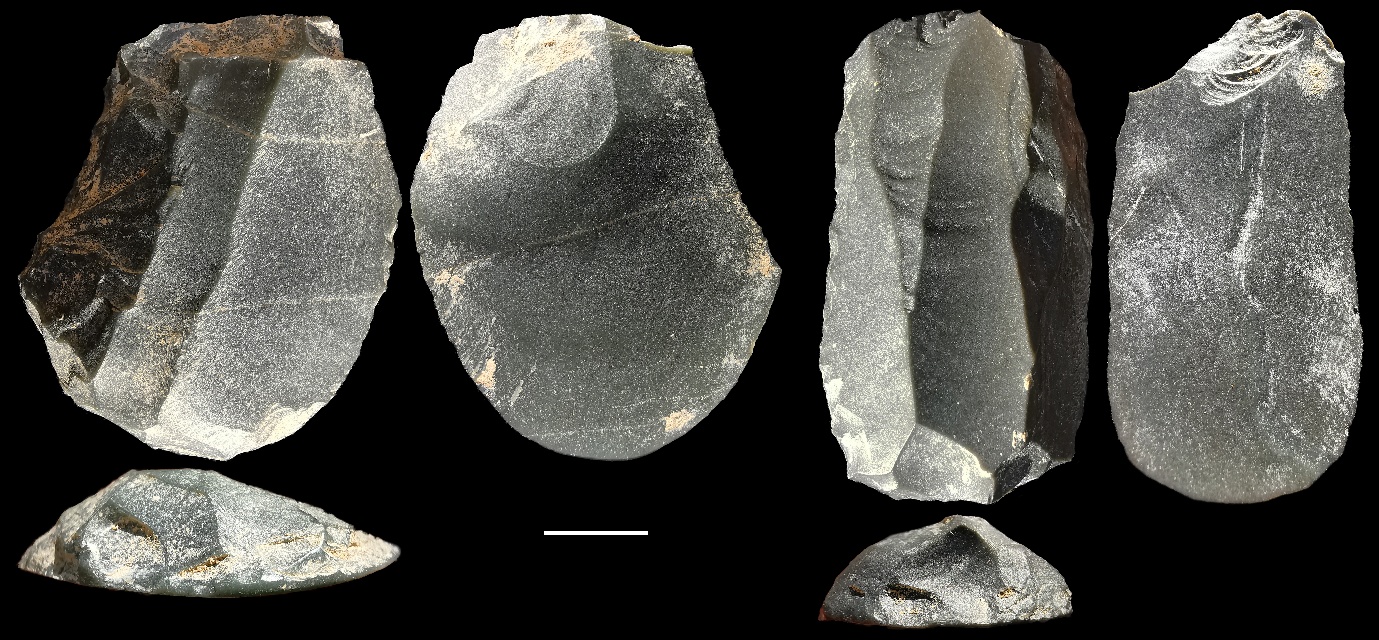
*

*Supplementary Figure 23. Chert end-scrapers with rounding wear on their retouched ends from SAU2 T2B layers 4 (left) and 5 (right) (phase 1). Scale is 1 cm.*

*
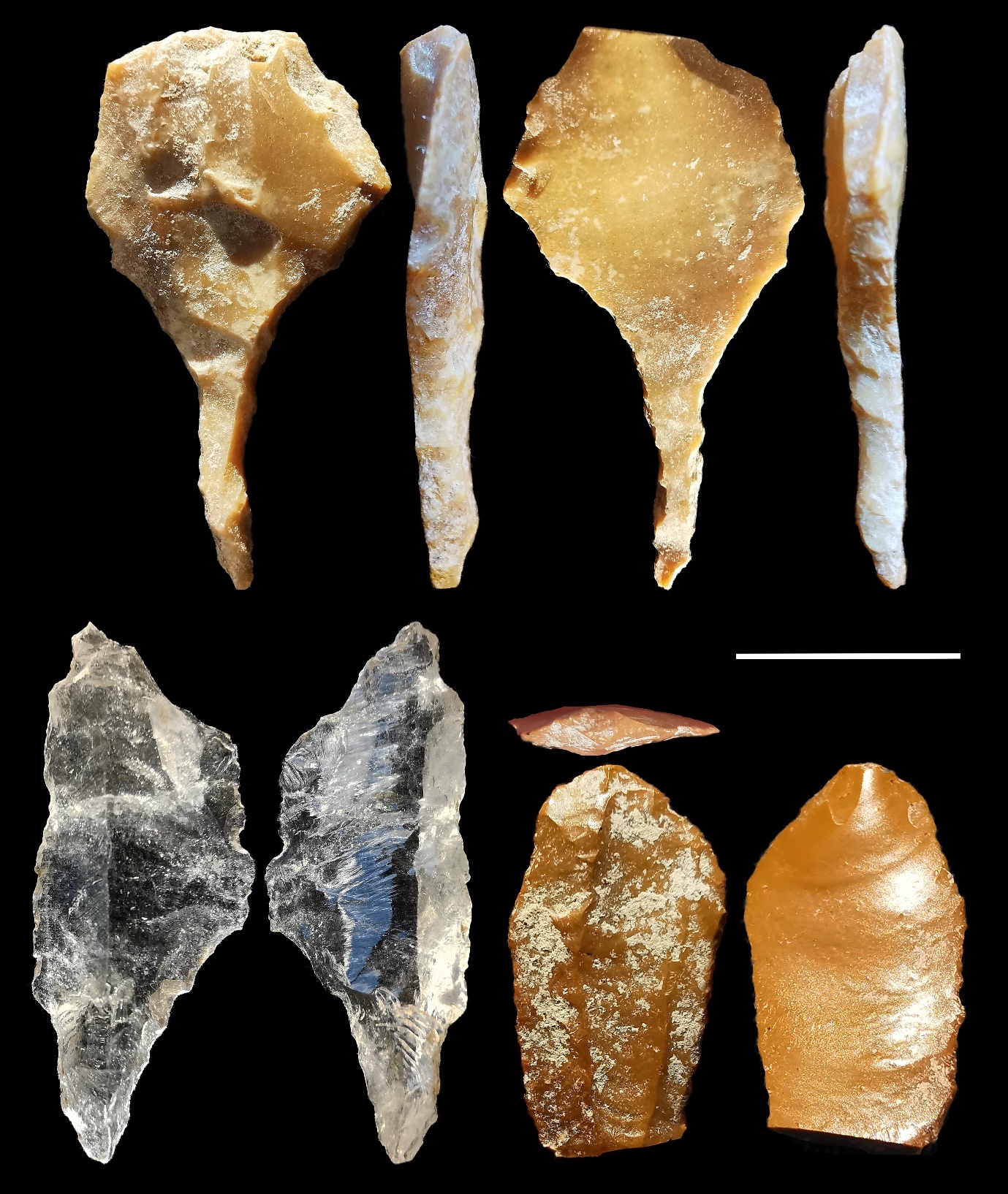
*

*Supplementary Figure 24. Drills and sickle blade from the SAU2 T1B excavations. Above is a chert drill from T1B layer 4 (phase 2); bottom left is a crystal quartz drill from T1B layer 5 (phase 2); bottom right is the proximal portion of a chert marginally retouched blade with a ground platform from T1B layer 4 (phase 2). Note the sickle gloss on the right edge next to the marginal retouch on the broken chert blade. Scale is 1 cm.*

*
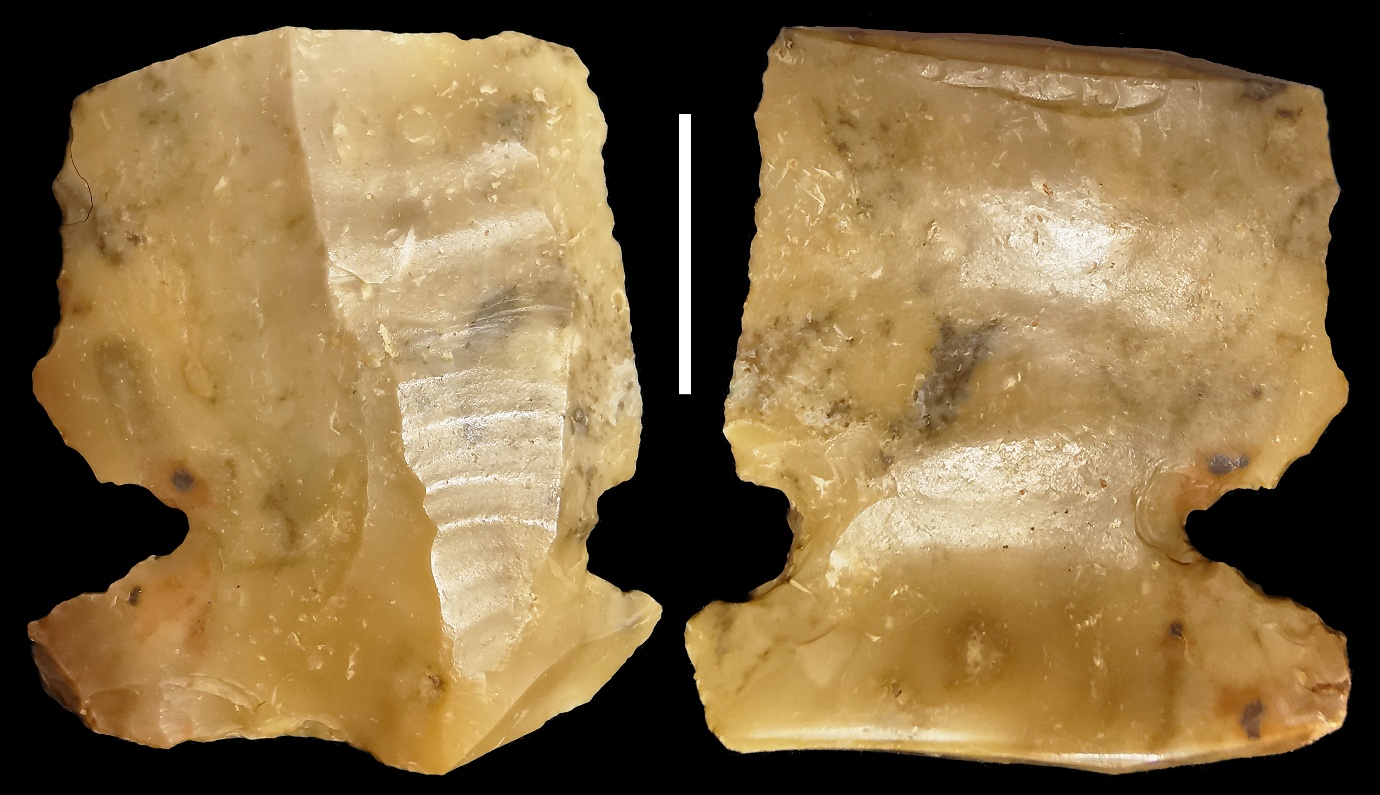
*

*Supplementary Figure 25. Broken proximal of a chert El Khiam point from the surface at SAU4 (Fig. 1). Scale is 1 cm.*

*
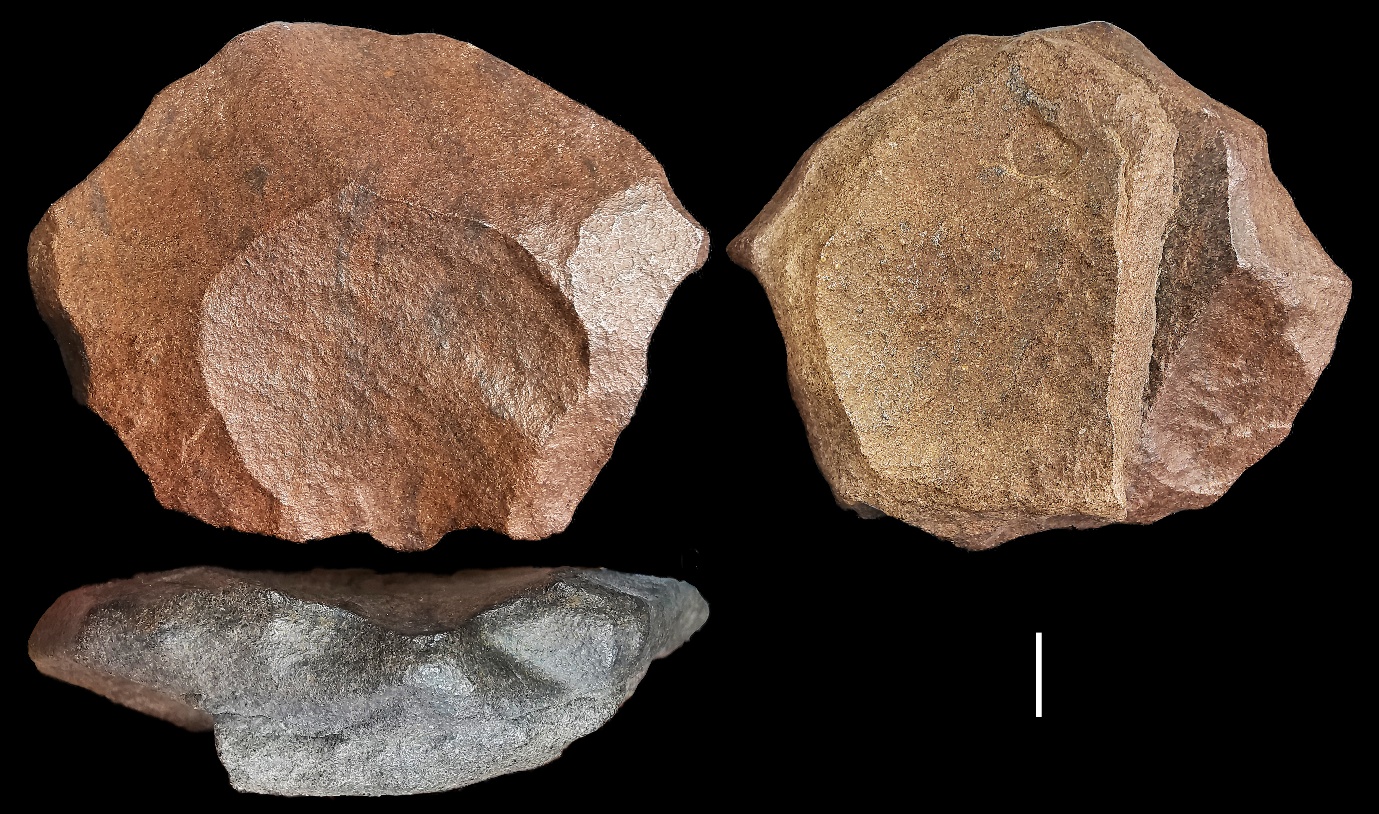
*

*Supplementary Figure 26. Ferruginous Levallois core from the surface near SAU1. Scale is 1 cm.*

**Supplementary Note 7: Obsidian Sourcing**

*Supplementary Table 12: Summary statistics and geochemical values of pXRF analysis and reference collection used for fingerprinting archaeological artefacts from Sahout, Jebel Arnaan, and Jebel Misma.*

| **Reading #** | **Protocol Name** | **Provenance** | **Ti** | **Mn** | **Fe** | **Zn** | **As** | **Rb** | **Sr** | **Y** | **Zr** | **Nb** | **Sn** | **Ba** | **Pb** | **Th** | **Group** | **Ref.** | **Method** |
| --- | --- | --- | --- | --- | --- | --- | --- | --- | --- | --- | --- | --- | --- | --- | --- | --- | --- | --- | --- |
| 5 | Geochem (3-Beam) | Archaeological  ARN3 T2 L5 | 161 | 311 | 19901 | 466 | 16 | 356 |  | 273 | 2548 | 208 |  | 94 | 58 | 39 |  |  |  |
| 6 | Geochem (3-Beam) | Archaeological  ARN3 T2 L5 | 152 | 297 | 18457 | 425 | 12 | 334 |  | 264 | 2474 | 205 | 17 | 85 | 53 | 35 |  |  |  |
| 7 | Geochem (3-Beam) | Archaeological  ARN3 T2 L6 | 164 | 326 | 20043 | 473 | 14 | 353 |  | 267 | 2529 | 203 | 14 | 96 | 59 | 39 |  |  |  |
| 8 | Geochem (3-Beam) | Archaeological  ARN3 T1 L1 | 147 | 251 | 16480 | 395 | 11 | 320 |  | 259 | 2466 | 206 | 19 | 88 | 52 | 26 |  |  |  |
| 9 | Geochem (3-Beam) | Archaeological  ARN3 T1 L5 | 167 | 271 | 18012 | 396 | 11 | 324 |  | 262 | 2485 | 207 | 22 | 74 | 54 | 28 |  |  |  |
| 11 | Geochem (3-Beam) | Archaeological  SAU2 T1B L4 | 153 | 294 | 17781 | 414 | 11 | 327 |  | 262 | 2483 | 207 | 16 | 68 | 58 | 34 |  |  |  |
| 12 | Geochem (3-Beam) | Archaeological  SAU2 T1B L5 | 153 | 270 | 17306 | 418 | 15 | 325 |  | 260 | 2468 | 208 | 22 | 64 | 52 | 30 |  |  |  |
| 13 | Geochem (3-Beam) | Archaeological  JMI8 T1 L1 | 145 | 277 | 16788 | 398 | 9 | 317 |  | 257 | 2441 | 207 | 34 | 91 | 55 | 29 |  |  |  |
| 14 | Geochem (3-Beam) | Archaeological  ARN3 T2 L5 | 138 | 270 | 17616 | 412 | 9 | 323 |  | 260 | 2480 | 206 | 22 | 66 | 55 | 31 |  |  |  |
| 15 | Geochem (3-Beam) | Archaeological  JMI8 T1 L3 | 118 | 273 | 16581 | 397 | 10 | 321 |  | 261 | 2453 | 205 | 28 | 95 | 54 | 35 |  |  |  |
| 16 | Geochem (3-Beam) | Archaeological  ARN3 T2 L5 | 133 | 274 | 18013 | 411 | 11 | 325 |  | 264 | 2488 | 207 | 22 | 77 | 54 | 28 |  |  |  |
| 17 | Geochem (3-Beam) | Archaeological SAU2 T1B L3 | 142 | 271 | 17017 | 406 | 12 | 319 |  | 255 | 2428 | 203 | 28 | 72 | 51 | 29 |  |  |  |
| 18 | Geochem (3-Beam) | Archaeological  ARN3 T2 L5 | 150 | 264 | 17380 | 386 | 12 | 320 |  | 262 | 2475 | 206 | 23 | 95 | 53 | 29 |  |  |  |
| 19 | Geochem (3-Beam) | Archaeological  SAU2 T2B L2 | 185 | 313 | 18481 | 434 | 11 | 337 |  | 265 | 2501 | 208 | 21 | 82 | 56 | 28 |  |  |  |
| 20 | Geochem (3-Beam) | Archaeological  ARN3 T2 L5 | 202 | 250 | 17911 | 398 | 13 | 322 |  | 259 | 2484 | 205 | 22 | 139 | 52 | 33 |  |  |  |
| 21 | Geochem (3-Beam) | Archaeological  ARN3 T2 L6 | 137 | 308 | 18346 | 436 | 15 | 334 |  | 265 | 2507 | 208 | 17 | 79 | 56 | 31 |  |  |  |
| 22 | Geochem (3-Beam) | Archaeological ARN3 T2 L6 | 177 | 329 | 21266 | 476 | 15 | 360 |  | 271 | 2566 | 207 |  | 73 | 62 | 41 |  |  |  |
| 31 | Geochem (3-Beam) | Archaeological  SAU2 T2B L4 | 131 | 268 | 16810 | 387 | 15 | 316 |  | 253 | 2418 | 204 | 37 | 52 | 49 | 45 |  |  |  |
| 34 | Geochem (3-Beam) | Archaeological  SAU2 T1B L2 | 176 | 281 | 16744 | 383 | 12 | 315 |  | 251 | 2425 | 204 | 41 |  | 53 | 39 |  |  |  |
| 36 | Geochem (3-Beam) | Archaeological  JMI8 T1 L6 | 140 | 235 | 16507 | 375 | 11 | 303 |  | 248 | 2389 | 204 | 44 |  | 50 | 29 |  |  |  |
| 37 | Geochem (3-Beam) | Archaeological  ARN3 T2 L7 | 136 | 285 | 16786 | 396 | 13 | 313 |  | 256 | 2431 | 206 | 55 |  | 52 | 40 |  |  |  |
| 38 | Geochem (3-Beam) | Archaeological  ARN3 T2 L7 | 164 | 267 | 18104 | 415 | 12 | 327 |  | 260 | 2473 | 205 | 19 | 86 | 54 | 40 |  |  |  |
| 39 | Geochem (3-Beam) | Archaeological  SAU2 T2B L3 | 141 | 258 | 15954 | 380 | 13 | 305 |  | 241 | 2293 | 190 | 17 | 66 | 48 | 39 |  |  |  |
| 48 | Geochem (3-Beam) | Archaeological  SAU2 T1B L4 | 152 | 273 | 16537 | 401 | 12 | 320 |  | 259 | 2472 | 210 | 32 | 73 | 53 | 28 |  |  |  |
| 4 | Geochem (3-Beam) | Jabal Al Abyad | 145 | 266 | 16211 | 367 | 11 | 302 |  | 244 | 2342 | 200 | 46 |  | 51 | 38 |  |  |  |
| 40 | Geochem (3-Beam) | Jabal Al Abyad | 136 | 230 | 15380 | 363 | 11 | 297 |  | 248 | 2350 | 200 | 37 | 81 | 50 | 34 |  |  |  |
| 41 | Geochem (3-Beam) | Jabal Al Abyad | 148 | 272 | 16886 | 405 | 16 | 322 |  | 257 | 2433 | 204 | 19 | 66 | 49 | 47 |  |  |  |
| 42 | Geochem (3-Beam) | Jabal Al Abyad | 150 | 274 | 16664 | 402 | 13 | 320 |  | 254 | 2438 | 201 | 22 | 66 | 52 | 48 |  |  |  |
| 43 | Geochem (3-Beam) | Jabal Al Abyad | 115 | 254 | 16254 | 389 | 11 | 314 |  | 253 | 2403 | 204 | 39 | 55 | 52 | 33 |  |  |  |
| 44 | Geochem (3-Beam) | Jabal Al Abyad | 135 | 256 | 16143 | 392 | 12 | 312 |  | 253 | 2399 | 205 | 41 | 62 | 51 | 37 |  |  |  |
| 45 | Geochem (3-Beam) | Jabal Al Abyad | 114 | 263 | 16648 | 396 | 13 | 319 |  | 259 | 2452 | 209 | 41 | 48 | 52 | 29 |  |  |  |
| 46 | Geochem (3-Beam) | Jabal Al Abyad | 169 | 255 | 14927 | 290 | 10 | 247 |  | 197 | 1912 | 160 | 55 | 56 | 38 | 34 |  |  |  |
| 47 | Geochem (3-Beam) | Jabal Al Abyad | 170 | 259 | 16450 | 390 | 12 | 314 |  | 257 | 2431 | 204 | 53 |  | 52 | 30 |  |  |  |
| 50 |  | Jabal Al Abyad 1 | 600 | 379 | 20913 |  |  | 278 | 2 | 207 | 2193 | 264 |  |  |  |  | 10 | ^36^ | EDX /  ICPMS |
| 51 |  | Jabal Al Abyad 2 | 600 | 379 | 21123 |  |  | 279 | 2 | 211 | 2202 | 268 |  |  |  |  | 11 | ^36^ | EDX  ICPMS |
| 60 |  | Bingöl B | 1353 | 370 | 15187 | 67 | 21 | 230 | 57 | 32 | 334 | 18 |  | 486 | 39 | 29 | 20 | ^37^ | XRF |
| 61 |  | Bingöl A | 1331 | 564 | 28760 | 214 | 36 | 259 | 5 | 129 | 1258 | 62 |  | 12 | 81 | 32 | 21 | ^37^ | XRF |
| 62 |  | Nemrut Dağ | 1230 | 529 | 24435 | 203 | 32 | 228 | 5 | 125 | 1290 | 70 |  | 21 | 66 | 27 | 22 | ^37^ | XRF |
| 63 |  | Mescrut Koavk | 806 | 457 | 12628 | 142 | 28 | 195 | 11 | 65 | 251 | 84 |  | 6 | 60 | 24 | 23 | ^37^ | XRF |
| 70 |  | Jebel Lisi (18) | 703 | 312 | 16955 |  |  | 573 | 0.4 | 175 | 1345 | 320 |  |  |  | 62 | 30 | ^38^ | ICPMS |
| 71 |  | Jebel Isbil 1 (15) | 1880 | 1589 | 46037 |  |  | 245 | 1.1 | 169 | 1770 | 228 |  |  |  | 32 | 31 | ^38^ | ICPMS |
| 72 |  | Jebel Isbil 2 (3) | 1418 | 1071 | 37617 |  |  | 271 | 0.2 | 152 | 1852 | 229 |  |  |  | 35 | 32 | ^38^ | ICPMS |
| 73 |  | Al-Gharga (9) | 683 | 283 | 16452 |  |  | 658 | 0.3 | 196 | 1397 | 365 |  |  |  | 70 | 33 | ^38^ | ICPMS |
| 74 |  | Jirab al Souf (20) | 557 | 217 | 14680 |  |  | 738 | 1 | 234 | 1377 | 314 |  |  |  | 72 | 34 | ^38^ | ICPMS |
| 75 |  | Hayd al Halal 1 (18) | 599 | 454 | 11281 |  |  | 164 | 4.3 | 55 | 274 | 94 |  |  |  | 15 | 35 | ^38^ | ICPMS |
| 76 |  | Hayd al Halal 2 (4) | 1015 | 486 | 11117 |  |  | 178 | 33 | 37 | 264 | 67 |  |  |  | 15 | 36 | ^38^ | ICPMS |
| 77 |  | Yafa’ Ridge 1 (31) | 1036 | 431 | 21578 |  |  | 286 | 0.2 | 97 | 1015 | 123 |  |  |  | 27 | 37 | ^38^ | ICPMS |
| 78 |  | Yafa’ Ridge 2 (4) | 1239 | 399 | 34277 |  |  | 172 | 0.4 | 66 | 682 | 91 |  |  |  | 19 | 38 | ^38^ | ICPMS |
| 79 |  | Yafa’ Ridge 3 (1) | 1493 | 507 | 34041 |  |  | 124 | 2.2 | 39 | 373 | 62 |  |  |  | 12 | 39 | ^38^ | ICPMS |
| 80 |  | Afar 1 (4) | 713 | 244 | 10200 |  |  | 115 | 6.8 | 60 | 281 | 63 |  |  |  | 10 | 40 | ^38^ | ICPMS |

***
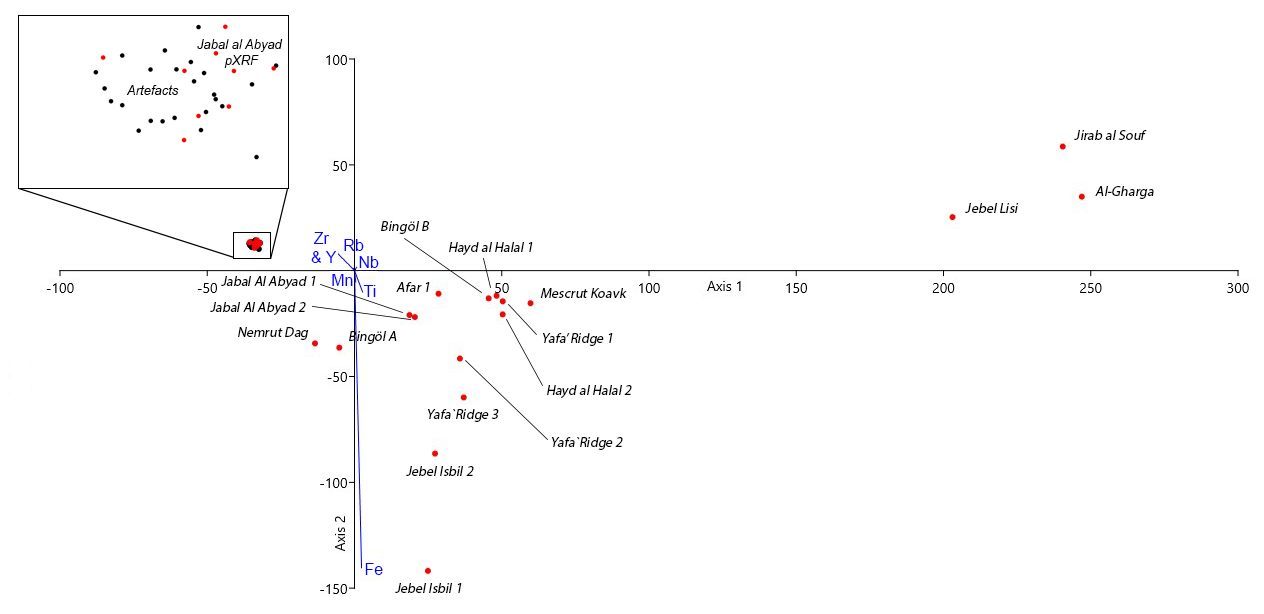
***

*Supplementary Figure 27. Discriminant Function Analysis of obsidian artefacts and reference collections. Black dots represent artefacts, red dots obsidian sources from the Arabian Peninsula and well-known sources from the Taurus Mountains, Anatolia^36-38^. Factor loadings are represented by blue lines.*

Slight variation between our samples and published data from Jebel Al-Abyad (particularly Ti and Fe) can be traced to the use of different geochemical techniques for the analysis of the geological samples, inductively coupled plasma mass spectrometry and energy dispersive X-ray spectrometry in the case of El-Gameel^36^ and our analysis utilising portable X-ray florescence.

**
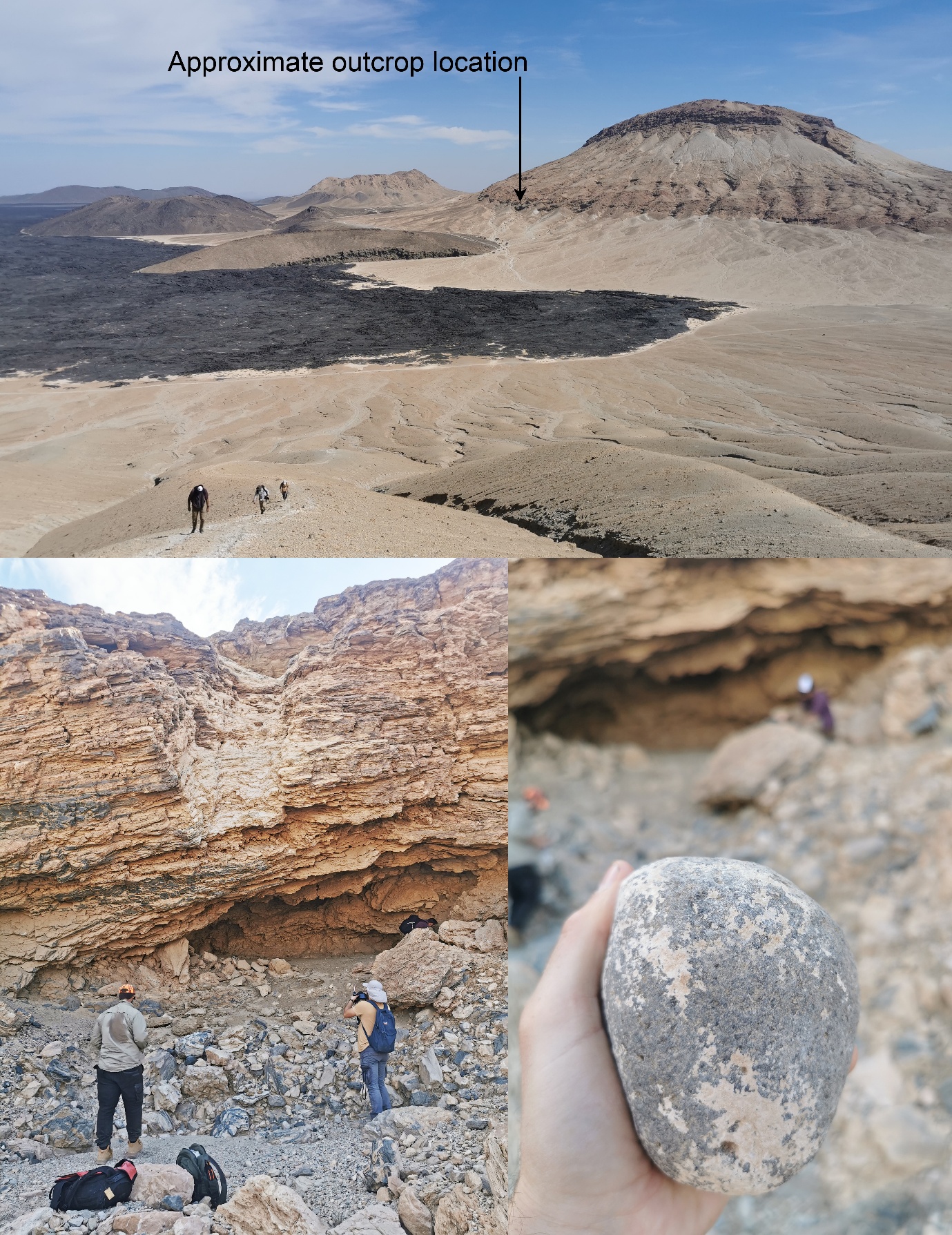
**

*Supplementary Figure 28. Jebel Al Abyad as viewed from the white volcano (above), and an obsidian outcrop (bottom left) where a hammerstone was observed (bottom right) that may have been used for quarrying. Note the greenish-grey obsidian clasts littering the ground around where the two figures are standing at the outcrop. Photographs by CS.*

**Supplementary Note 8: Rock art production**

The two wedge-shaped pecking stones recovered from Sahout exhibit flake scars on their distal ends, perhaps from battering damage during the creation of petroglyphs or as deliberate sharpening of the working ends to allow greater precision and penetration when pecking (Supplementary Figures 29 & 30). Unifacial scars were also observed on pecking stones from Jebel Arnaan^26^. Deep individual peck marks are visible on some of the Sahout engravings, including the image of a woman in profile (Supplementary Figure 30). In other engravings peck marks were subsequently smoothed to form continuous lines. The wedge-shaped morphology might have facilitated both the initial deep pecking and the subsequent smoothing process on the camel images.

**
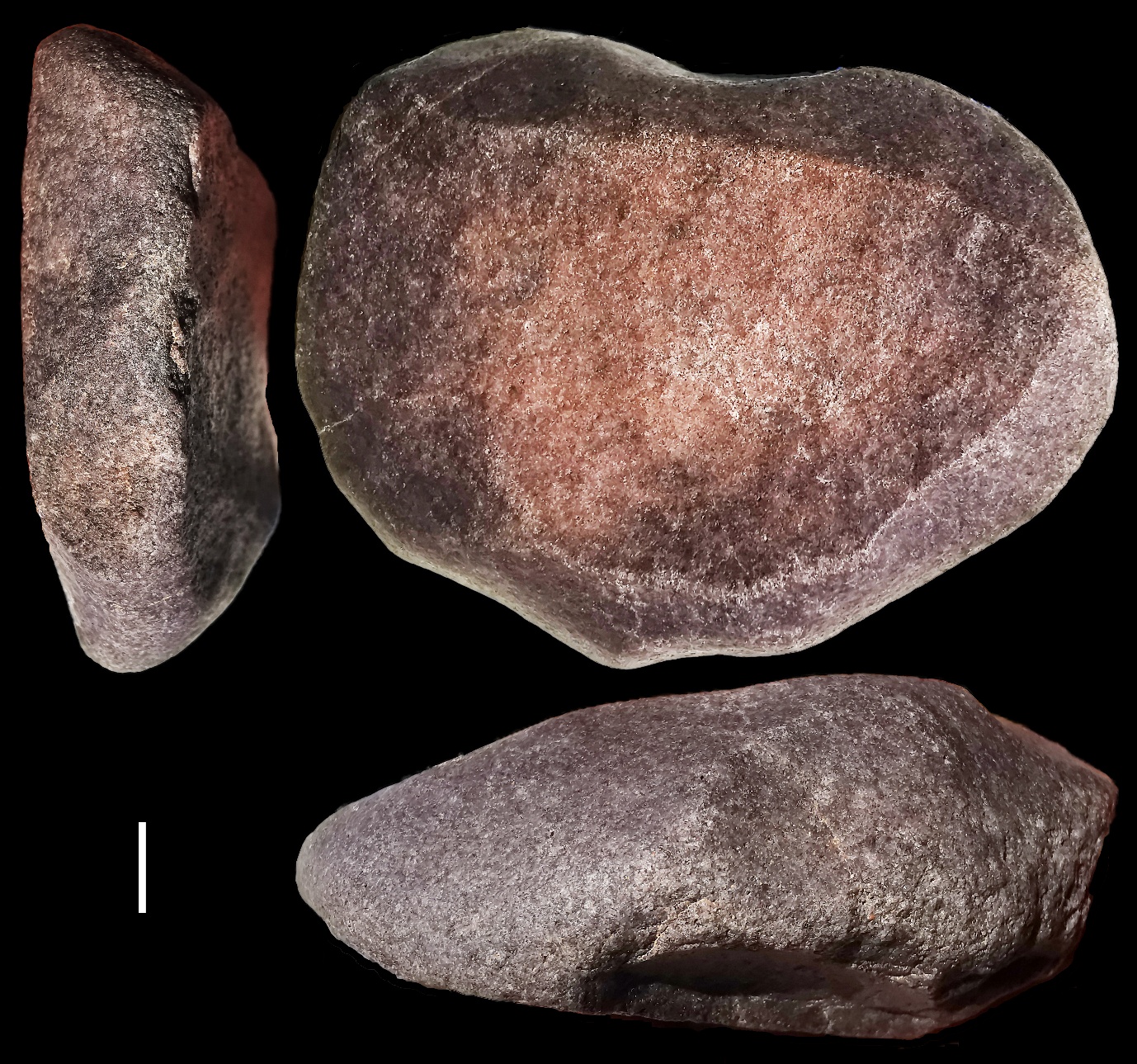
**

*Supplementary Figure 29. Ferruginous pecking stone from the surface at SAU1, found directly below panel SAU1_B^31^. Note the wedge-shaped profile of the piece and the battering on the narrow end (left). This specimen also has battering on its lateral edge shown in the profile view (bottom). Although it is heavily weathered there is a flake scar on the distal end of this piece. Scale is 1 cm.*

*
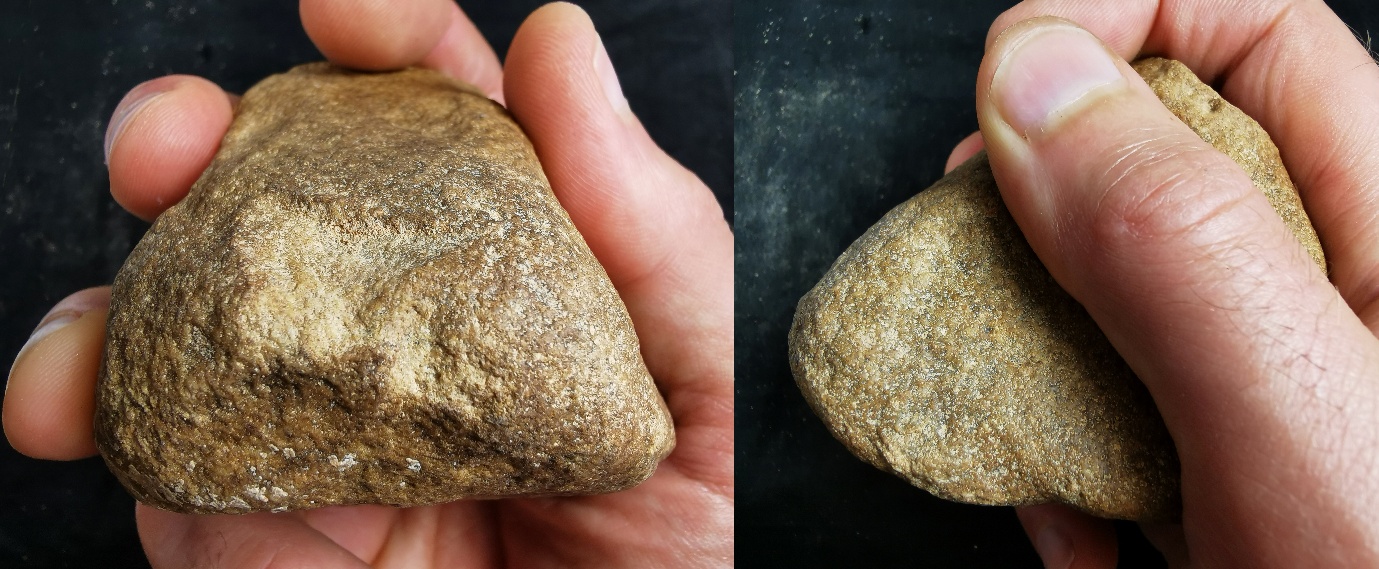
*

*Supplementary Figure 30. Quartzite pecking stone in the hand from SAU2 T1B layer 6 (phase 2). Note how the narrow end of the stone where the battering damage is located protrudes from the hand in a four-jaw chuck grip, with the broad end of the stone braced against the palm.*

The engraved rock art at Sahout covers various styles. In combination with dated archaeological deposits and superimpositions observed at other rock art localities in the region, particularly Jebel Arnaan and Jebel Misma^26^ a broad chronological sequence can be identified.

Superimpositions at Jebel Arnaan indicate that depictions of women in profile, such as the examples from Sahout in Supplementary Figures 31 and 32, are older than engravings of life-sized, naturalistic camels and other wildlife^26^. This latter period large animal depictions was split into two phases based on observable stylistic superimpositions^26^. Earlier life-sized animal engravings show extreme naturalism, with characteristics that potentially reflect individual animals, and are frequently shown in unusual poses, for example with the neck bent down, getting up or sitting down. Later life-sized engravings are also extremely detailed, but show more stylised, cartoonish features, particularly circular eyes and rounded muzzles, and in the case of ibex rounded horn ridges. These depictions appear to show generic animals rather than individuals, sometimes with several identical animals on the same panel^26^. In this phase, animals are also depicted in a more standardized pose, legs striding and neck/head held horizontal.

Panel SAU1_B shows two naturalistic camels superimposed over each other (Supplementary Figure 33), which highlights that the period of large naturalistic engravings spanned over a prolonged period, during which specific stylistic elements and artistic conventions were potentially maintained over several generations.

The rock art panel closest to the excavations at Sahout shows a highly stylised camel, life-sized but engraved in very simple lines, with a prominent jaw line, circular eye, and rounded muzzle (Supplementary Figure 34, left). On the other side of the same boulder, we recorded the most heavily stylised depiction of a camel at Sahout. The image is only partially preserved, showing the neck and head, with cartoonish circular eyes, rounded nose and lips, and a prominent jaw line (Supplementary Figure 34, right).

Archaeological deposits associated with life-sized animal engravings at Jebel Arnaan, Jebel Misma, and Sahout now suggest that this tradition corresponds with occupations dating to the PPNA and PPNB (Figure 8). Earlier engravings of stylised female figures may therefore relate to Natufian occupation recorded at Sahout.

Later Neolithic engravings recorded at Sahout are much simpler and smaller, and share similarities with well-known examples of Neolithic rock art from Shuwaymis^39^ and Jubbah^40^. These engravings are much smaller, with the body of the cattle engraving in Supplementary Figure 35 (top) measuring ca. 60 cm. The figure is depicted with a simple box-like body and very short, rudimentary legs. The head is completely feature-less and small. Similarly, the Neolithic depiction of a sheep (Supplementary Figure 35, bottom) shows stylised coat markings but lacks all other features with exception of a small tail, rudimentary ears, and backward curving horns.

*
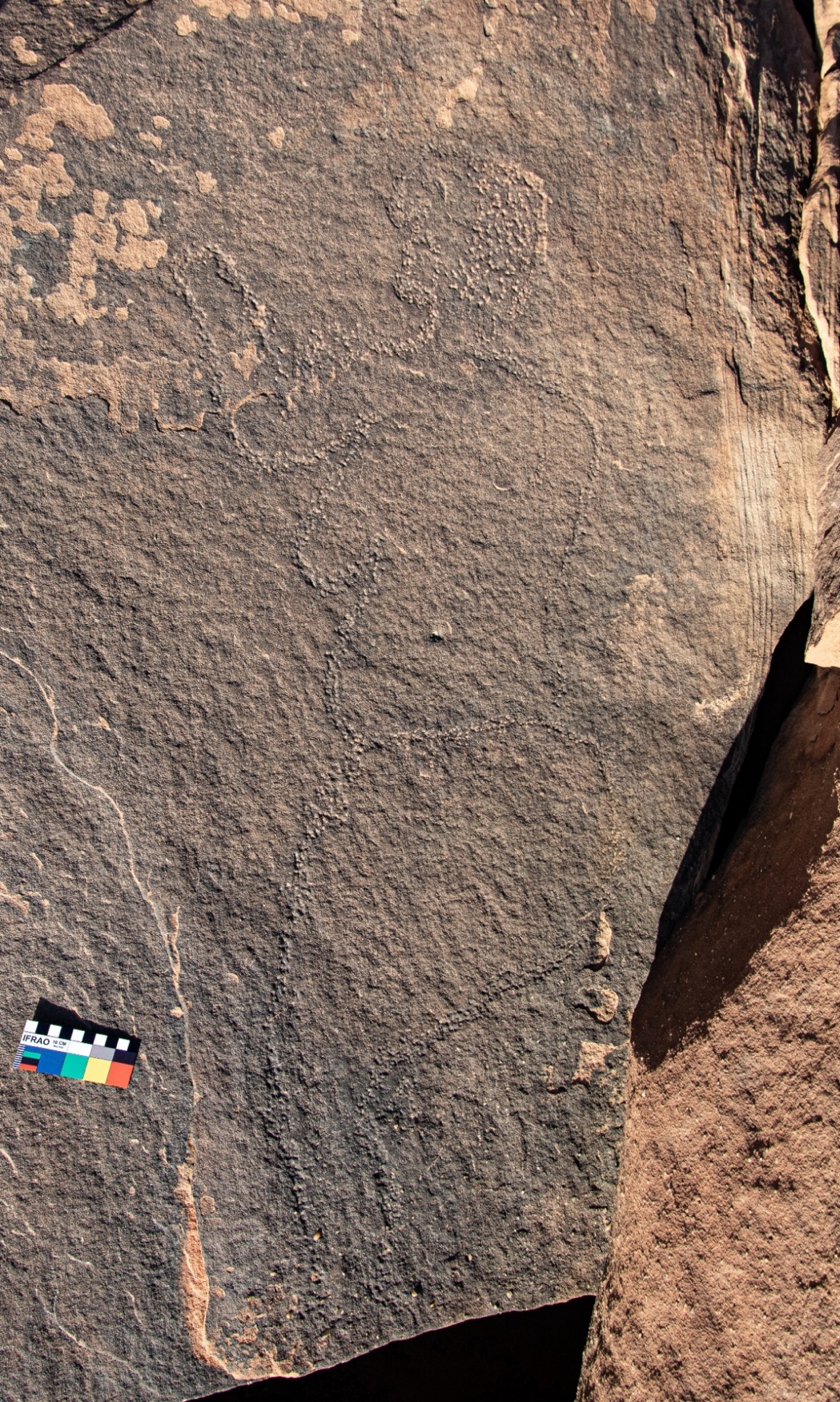
*

*Supplementary Figure 31. Depiction of a woman in profile on a horizontal sandstone outcrop at SAU4. Note the deep individual peck marks and the lack of a secondary process of smoothing to join the pecking into a continuous line. Scale is 10 cm.*

*
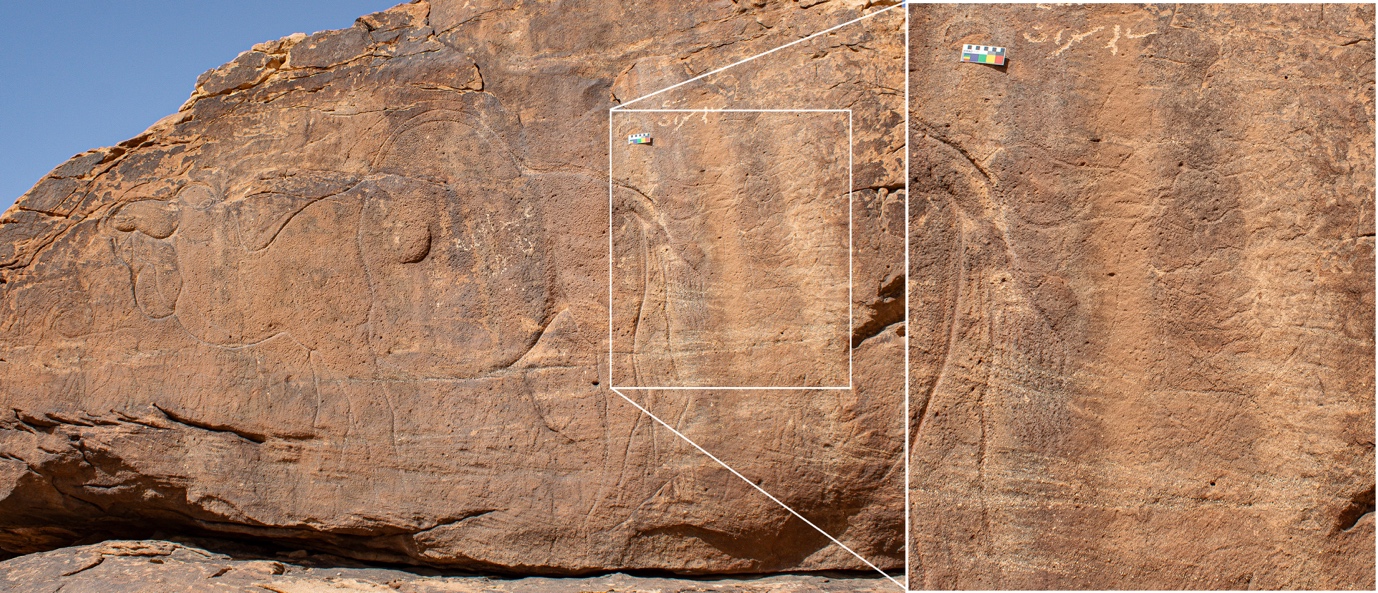
*

*Supplementary Figure 32. Panel SAU3 showing a large camel engraving and an earlier engraving of a female figure. Inset: detail photo of the female figure. Note the head of the female figure is superimposed with camel engravings and is no longer visible.*

*
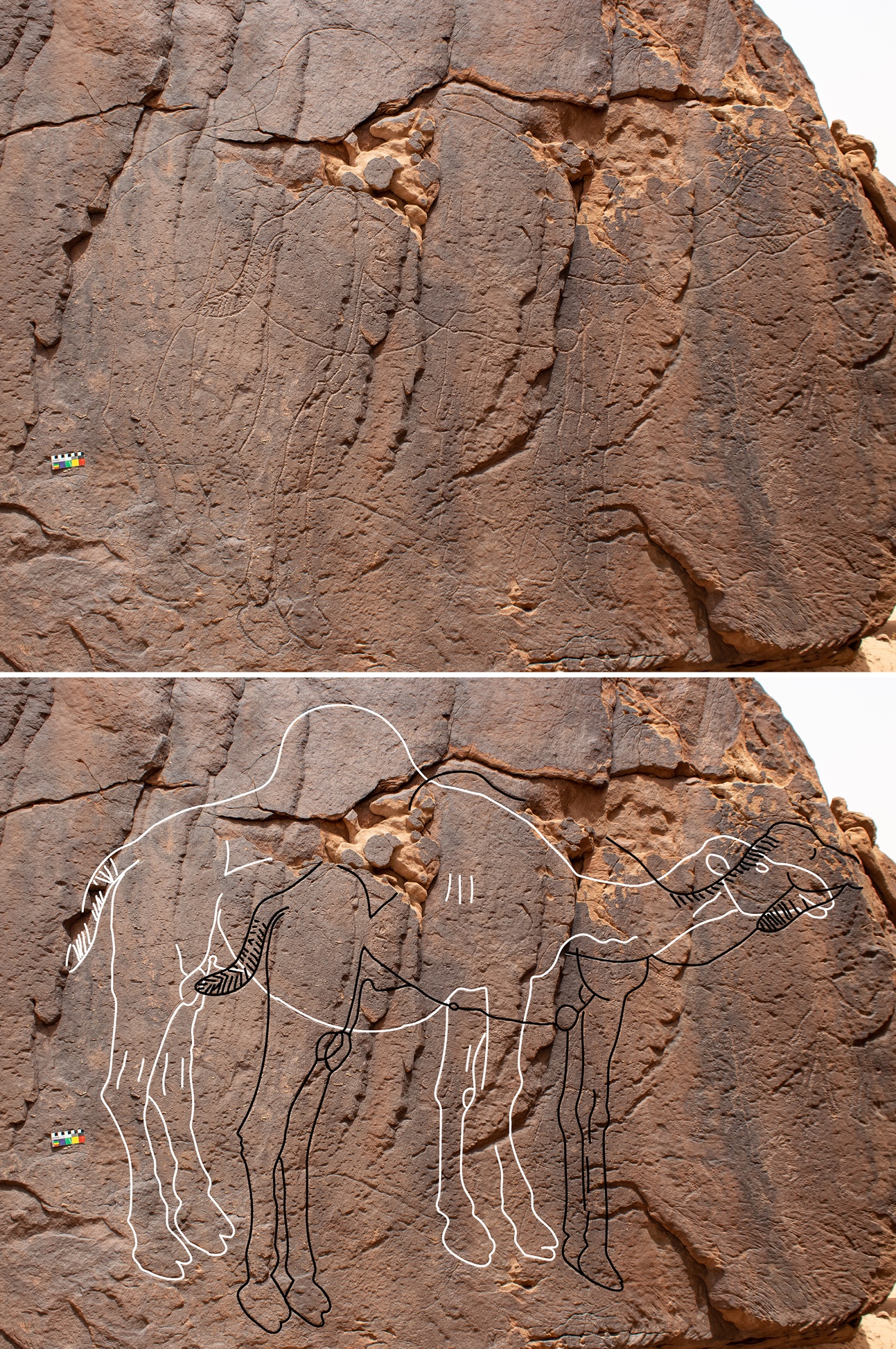
*

*Supplementary Figure 33. Extremely naturalistic camel depictions at Sahout. Panel SAU1_B, showing two naturalistic camels superimposed over each other.*

*
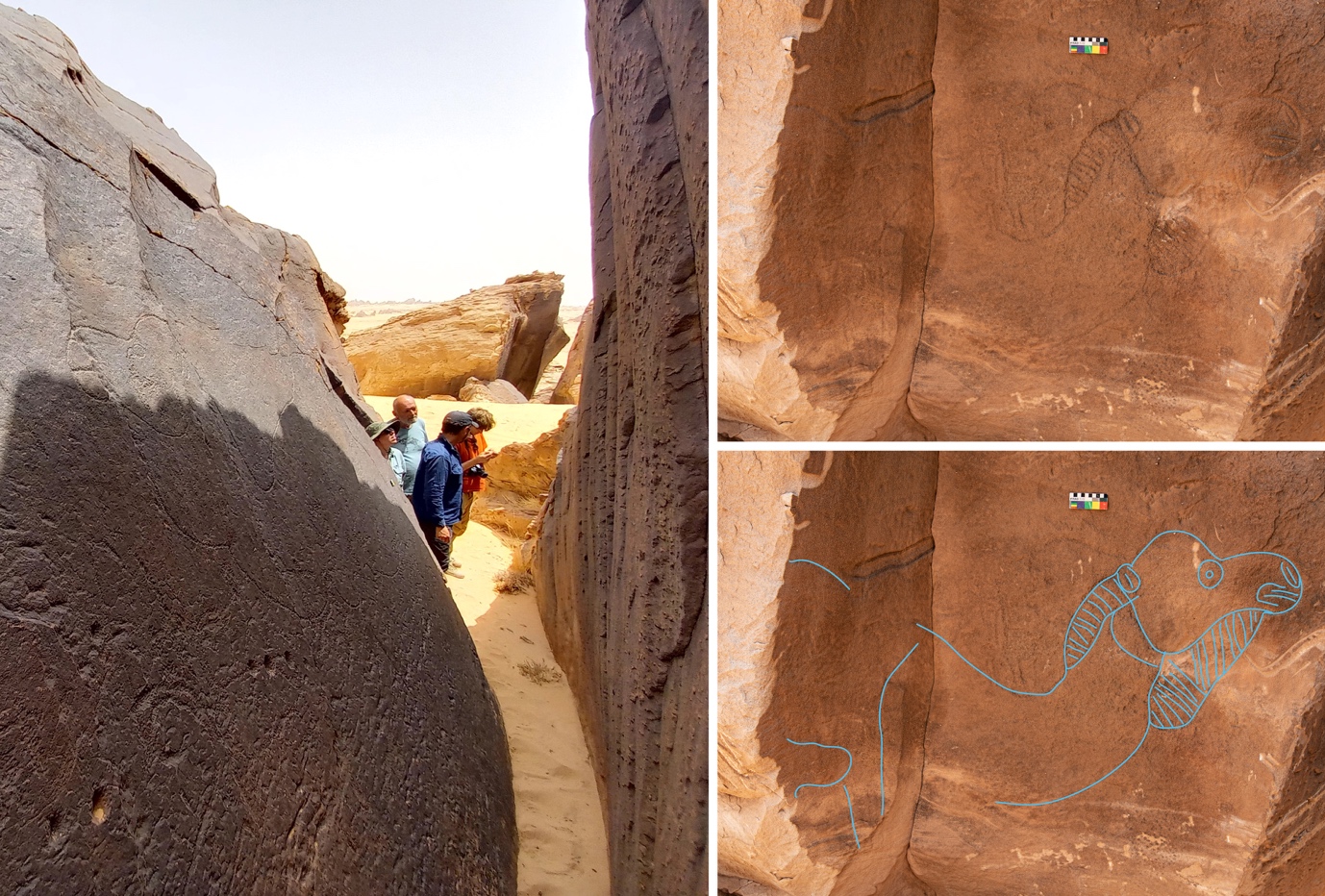
*

*Supplementary Figure 34. Stylised camel depictions with cartoonish features from Sahout. Left: Panel SAU2_A, the closest panel to the Sahout excavations, showing a stylised but life-sized camel. Right: Panel SAU2_D showing an extremely stylised life-sized camel with cartoonish features visible in the circular eye, rounded nose and lips, and typical prominent jaw line.*

**
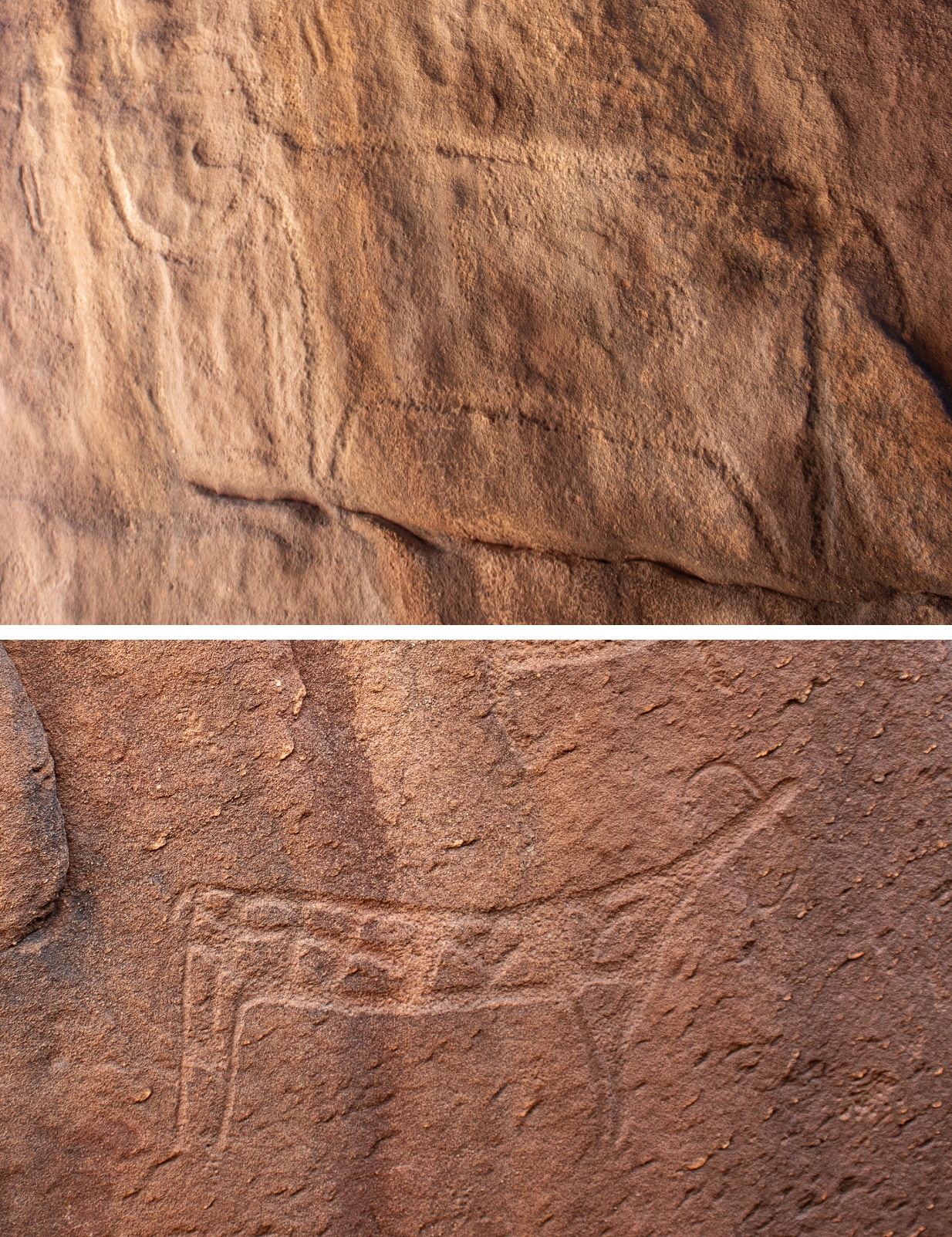
**

*Supplementary Figure 35. Cattle (above) and sheep (below) engraved in the Jubbah style. The cattle image is c. 60 cm long and the sheep image c. 20 cm.*

**Supplementary References**

1 Amin, M. T., Mahmoud, S. & Alazba, A. Observations, projections and impacts of climate change on water resources in Arabian Peninsula: current and future scenarios. *Environmental Earth Sciences* **75**, 864 (2016).

2 Taylor, G. & Eggleton, R. Silcrete: an Australian perspective. *Australian Journal of Earth Sciences* **64**, 987-1016 (2017).

3 Bøtter-Jensen, L., Andersen, C. E., Duller, G. A. T. & Murray, A. S. Developments in radiation, stimulation and observation facilities in luminescence measurements. *Radiation Measurements* **37**, 535-541 (2003). <https://doi.org:https://doi.org/10.1016/S1350-4487(03)00020-9>

4 Duller, G. A. T., Bøtter-Jensen, L., Murray, A. S. & Truscott, A. J. Single grain laser luminescence (SGLL) measurements using a novel automated reader. *Nuclear Instruments and Methods in Physics Research Section B: Beam Interactions with Materials and Atoms* **155**, 506-514 (1999). <https://doi.org:https://doi.org/10.1016/S0168-583X(99)00488-7>

5 Armitage, S. J. & Bailey, R. M. The measured dependence of laboratory beta dose rates on sample grain size. *Radiation Measurements* **39**, 123-127 (2005). <https://doi.org:https://doi.org/10.1016/j.radmeas.2004.06.008>

6 Ballarini, M., Wintle, A. G. & Wallinga, J. Spatial variation of dose rate from beta sources as measured using single grains. *Ancient TL* **24**, 1-7 (2006).

7 Armitage, S. J. *et al.* The Southern Route “Out of Africa”: Evidence for an Early Expansion of Modern Humans into Arabia. *Science* **331**, 453-456 (2011). <https://doi.org:doi:10.1126/science.1199113>

8 Murray, A. S. & Wintle, A. G. Luminescence dating of quartz using an improved single-aliquot regenerative-dose protocol. *Radiation Measurements* **32**, 57-73 (2000). <https://doi.org:https://doi.org/10.1016/S1350-4487(99)00253-X>

9 Murray, A. S. & Wintle, A. G. The single aliquot regenerative dose protocol: potential for improvements in reliability. *Radiation Measurements* **37**, 377-381 (2003). <https://doi.org:https://doi.org/10.1016/S1350-4487(03)00053-2>

10 Duller, G. A. T. Distinguishing quartz and feldspar in single grain luminescence measurements. *Radiation measurements* **37**, 161-165 (2003).

11 Peng, J., Dong, Z., Han, F., Long, H. & Xiangjun, L. R package numOSL: numeric routines for optically stimulated luminescence dating. *Ancient TL* **31**, 41-48 (2013).

12 Peng, J. & Li, B. Single-aliquot Regenerative-Dose (SAR) and Standardised Growth Curve (SGC) Equivalent Dose Determination in a Batch Model Using the R Package ‘numOSL’. *Ancient TL* **35** (2017).

13 Hansen, V., Murray, A., Buylaert, J.-P., Yeo, E.-Y. & Thomsen, K. A new irradiated quartz for beta source calibration. *Radiation Measurements* **81**, 123-127 (2015).

14 Galbraith, R. F. & Green, P. F. Estimating the Component Ages in a Finite Mixture. *Nuclear Tracks and Radiation Measurements* **17**, 197-206 (1990).

15 Galbraith, R. F. & Roberts, R. G. Statistical aspects of equivalent dose and error calculation and display in OSL dating: An overview and some recommendations. *Quaternary Geochronology* **11**, 1-27 (2012). <https://doi.org:https://doi.org/10.1016/j.quageo.2012.04.020>

16 Bøtter-Jensen, L. & Mejdahl, V. Assessment of beta dose-rate using a GM multicounter system. *International Journal of Radiation Applications and Instrumentation. Part D. Nuclear Tracks and Radiation Measurements* **14**, 187-191 (1988). <https://doi.org:https://doi.org/10.1016/1359-0189(88)90062-3>

17 De Corte, F. *et al.* Preparation and characterization of loess sediment for use as a reference material in the annual radiation dose determination for luminescence dating. *Journal of Radioanalytical and Nuclear Chemistry* **272**, 311-319 (2007). <https://doi.org:10.1007/s10967-007-0522-5>

18 Mercier, N. & Falguères, C. Field gamma dose-rate measurement with a NaI (Tl) detector: re-evaluation of the “threshold” technique. *Ancient TL* **25**, 1-4 (2007).

19 Duval, M. & Arnold, L. J. Field gamma dose-rate assessment in natural sedimentary contexts using LaBr3(Ce) and NaI(Tl) probes: A comparison between the “threshold” and “windows” techniques. *Applied Radiation and Isotopes* **74**, 36-45 (2013). <https://doi.org:https://doi.org/10.1016/j.apradiso.2012.12.006>

20 Bell, W. T. Attenuation factors for the absorbed radiation dose in quartz inclusions for thermoluminescence dating. *Ancient TL* **8**, 1-12 (1979).

21 Aitken, M. J. *Thermoluminescence Dating* (Academic Press, 1985).

22 Prescott, J. R. & Hutton, J. T. Cosmic ray contributions to dose rates for luminescence and ESR dating: Large depths and long-term time variations. *Radiation Measurements* **23**, 497-500 (1994). <https://doi.org:https://doi.org/10.1016/1350-4487(94)90086-8>

23 Vogel, J. S., Southon, J. R., Nelson, D. E. & Brown, T. A. Performance of catalytically condensed carbon for use in accelerator mass spectrometry. *Nuclear Instruments and Methods in Physics Research Section B: Beam Interactions with Materials and Atoms* **5**, 289-293 (1984).

24 OxCal v4.4.4 (2021).

25 Reimer, P. J. *et al.* The IntCal20 Northern Hemisphere radiocarbon age calibration curve (0–55 cal kBP). *Radiocarbon* **62**, 725-757 (2020).

26 Guagnin, M. *et al.* Monumental rock art illustrates that humans thrived in the Arabian Desert during the Pleistocene-Holocene transition. *Nature Communications* **16**, 8429 (2025).

27 Guagnin, M. *et al.* A tale of two hearth sites: Neolithic and intermittent mid to late Holocene occupations in the Jubbah oasis, northern Saudi Arabia. *Archaeological Research in Asia* **26**, 100278 (2021).

28 Shipman, P., Foster, G. & Schoeninger, M. Burnt bones and teeth: an experimental study of color, morphology, crystal structure and shrinkage. *Journal of archaeological science* **11**, 307-325 (1984).

29 Lucarini, G. *et al.* Plant, pigment, and bone processing in the Neolithic of northern Arabia–New evidence from Use-wear analysis of grinding tools at Jebel Oraf. *Plos one* **18**, e0291085 (2023).

30 Dinies, M., Plessen, B., Neef, R. & Kürschner, H. When the desert was green: Grassland expansion during the early Holocene in northwestern Arabia. *Quaternary International* **382**, 293-302 (2015).

31 Guagnin, M. *et al.* Before the Holocene humid period: Life-sized camel engravings and early occupations on the southern edge of the Nefud desert. *Archaeological Research in Asia* **36**, 100483 (2023).

32 Crassard, R. & Hilbert, Y. H. Bidirectional blade technology on naviform cores from northern Arabia: New evidence of Arabian‐Levantine interactions in the Neolithic. *Arabian archaeology and epigraphy* **31**, 93-104 (2020).

33 Hilbert, Y. H. *et al.* Epipalaeolithic occupation and palaeoenvironments of the southern Nefud desert, Saudi Arabia, during the Terminal Pleistocene and Early Holocene. *Journal of Archaeological Science* **50**, 460-474 (2014).

34 Edwards, P. C. A 14000 year-old hunter-gatherer's toolkit. *Antiquity* **81**, 865-876 (2007).

35 Guagnin, M. *et al.* The Holocene humid period in the Nefud Desert: Hunters and herders in the Jebel Oraf palaeolake basin, Saudi Arabia. *Journal of Arid Environments* **178**, 104146 (2020).

36 El-Gameel, K., Abdallah, S., Deevsalar, R. & Eliwa, H. New Insights into the Petrogenesis of Quaternary Peralkaline Volcanics, Jabal Al Abyad, Saudi Arabia. *Arabian Journal for Science and Engineering* **46**, 543-562 (2021).

37 Carter, T., Grant, S., Kartal, M., Coşkun, A. & Özkaya, V. Networks and neolithisation: sourcing obsidian from Körtik Tepe (SE Anatolia). *Journal of Archaeological Science* **40**, 556-569 (2013).

38 Khalidi, L. *et al.* Obsidian sources in highland Yemen and their relevance to archaeological research in the Red Sea region. *Journal of Archaeological Science* **37**, 2332-2345 (2010).

39 Guagnin, M. *et al.* Hunters and herders: Exploring the Neolithic transition in the rock art of Shuwaymis, Saudi Arabia. *Archaeological Research in Asia* **4**, 3-16 (2015).

40 Guagnin, M. *et al.* An illustrated prehistory of the Jubbah oasis: Reconstructing Holocene occupation patterns in north‐western Saudi Arabia from rock art and inscriptions. *Arabian Archaeology and Epigraphy* **28**, 138-152 (2017).
